# Kinome-Wide RNAi Screen Implicates at Least 5 Host Hepatocyte Kinases in *Plasmodium* Sporozoite Infection

**DOI:** 10.1371/journal.ppat.1000201

**Published:** 2008-11-07

**Authors:** Miguel Prudêncio, Cristina D. Rodrigues, Michael Hannus, Cécilie Martin, Eliana Real, Lígia A. Gonçalves, Céline Carret, Robert Dorkin, Ingo Röhl, Kerstin Jahn-Hoffmann, Adrian J. F. Luty, Robert Sauerwein, Christophe J. Echeverri, Maria M. Mota

**Affiliations:** 1 Unidade de Malária, Instituto de Medicina Molecular, Universidade de Lisboa, Lisboa, Portugal; 2 Instituto Gulbenkian de Ciência, Oeiras, Portugal; 3 Cenix BioScience GmbH, Dresden, Germany; 4 Alnylam Pharmaceuticals, Cambridge, Massachusetts, United States of America; 5 Roche Kulmbach GmbH, Kulmbach, Germany; 6 Department of Medical Microbiology, University Medical Centre, Nijmegen, The Netherlands; Albert Einstein College of Medicine, United States of America

## Abstract

*Plasmodium* sporozoites, the causative agent of malaria, are injected into their vertebrate host through the bite of an infected *Anopheles* mosquito, homing to the liver where they invade hepatocytes to proliferate and develop into merozoites that, upon reaching the bloodstream, give rise to the clinical phase of infection. To investigate how host cell signal transduction pathways affect hepatocyte infection, we used RNAi to systematically test the entire kinome and associated genes in human Huh7 hepatoma cells for their potential roles during infection by *P. berghei* sporozoites. The three-phase screen covered 727 genes, which were tested with a total of 2,307 individual siRNAs using an automated microscopy assay to quantify infection rates and qRT-PCR to assess silencing levels. Five protein kinases thereby emerged as top hits, all of which caused significant reductions in infection when silenced by RNAi. Follow-up validation experiments on one of these hits, PKCς (PKCzeta), confirmed the physiological relevance of our findings by reproducing the inhibitory effect on *P. berghei* infection in adult mice treated systemically with liposome-formulated PKCς-targeting siRNAs. Additional cell-based analyses using a pseudo-substrate inhibitor of PKCς added further RNAi-independent support, indicating a role for host PKCς on the invasion of hepatocytes by sporozoites. This study represents the first comprehensive, functional genomics-driven identification of novel host factors involved in *Plasmodium* sporozoite infection.

## Author Summary

During a mammalian malaria infection, *Plasmodium* sporozoites injected by an infected mosquito travel to the liver where they invade hepatocytes and multiply into thousands of new parasites. These newly formed merozoites are then released into the bloodstream where they infect red blood cells and cause the symptoms of the disease. Although asymptomatic, the liver stage of malaria is an obligatory step in the parasite's lifecycle and constitutes an appealing target for prophylatic intervention. The marked tropism of sporozoites for hepatocytes suggests the latter may provide the parasite with a molecular environment that it can exploit to its own benefit. The identification of host factors that influence hepatic infection can thus provide clues for potential anti-malarial strategies. To this end, we carried out an RNA interference screen of the entire human kinome and associated signaling molecules and assessed the effect of knockdown of their expression in the infection of a human hepatoma cell line by *Plasmodium*. This strategy identified at least 5 kinases whose down-regulation leads to a marked decrease in infection. Further characterisation of one of these proteins, PKCζ, confirmed that it plays a role in infection by influencing the parasite's invasion of the host liver cells.

## Introduction

Although malaria has long been a devastating killer for the most vulnerable populations in countries of sub-Saharan Africa and other developing nations, our understanding of the early host-parasite interactions underlying this infectious disease remains far from complete. In fact, the first stage of a malaria infection, which occurs in the liver once the *Plasmodium* parasite has been delivered through the bite of an infected female *Anopheles* mosquito, is still clearly under-studied today.

Once inside the mammalian host, *Plasmodium* sporozoites, the motile form that is delivered in the mosquito's saliva, display a marked tropism for hepatocytes, the cells that enable the remarkable replication process that will give rise to thousands of merozoites from each invading parasite (reviewed in [1]). As a first step towards infection, several hepatocytes are transiently traversed by the sporozoite before one cell is productively invaded, leading to the formation of a parasitophorous vacuole [2]. Within this cytosolic vacuole, the subsequent development and asexual replication of *Plasmodium*, constituting so-called exoerythrocytic forms (EEFs), achieve one of the fastest growth rates among all eukaryotic cells. The invaded hepatocyte eventually releases thousands of mature merozoites into the bloodstream [3], where these then invade erythrocytes, thereby initiating the so-called blood stage of infection and triggering the well-known symptoms of malaria. Both the strong tropism and obligate nature of the events that take place during liver infection suggest an essential requirement for hepatocyte-specific factors in enabling this complex lead-up to the blood stage. It is therefore of primary interest to identify and characterize the role of such host factors, as these may contribute to the design of rational interventional strategies for the development of novel prophylactic agents.

**Figure 1:**
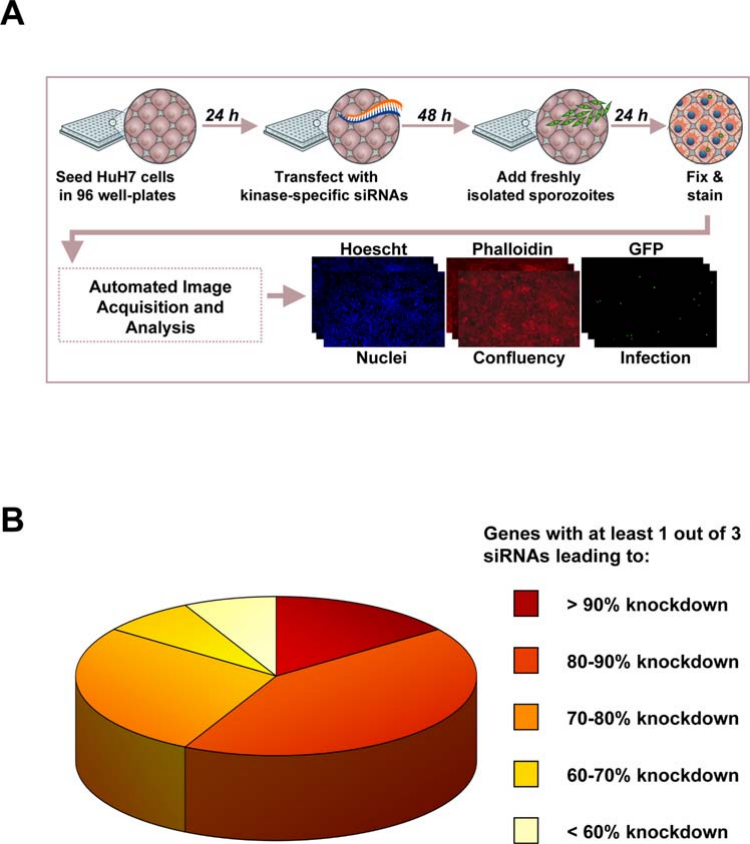
RNA interference screen strategy for identification of host factors affecting *Plasmodium* infection. (A) Experimental design of a high-throughput RNAi screen to identify host genes that influence *Plasmodium* sporozoite infection of host cells. (B) Validation of siRNA-mediated knock-down in Huh7 cells. Knock-down efficiency of 53 genes was evaluated by qRT-PCR following Huh7 cell transfection with 3 independent siRNAs per targeted gene.

To this end, we have used a cultured cell-based assay to study the process of liver infection by *Plasmodium* parasites at the cellular and molecular level. Using human Huh7 hepatoma cells and sporozoites of the rodent parasite *P. berghei* freshly isolated from infected *Anopheles* mosquitoes, we have established a high throughput assay system (Figure 1A) that, combined with high content readouts using automated microscopy, and quantitative RT-PCR (qRT-PCR), can be used for RNA interference (RNAi) and/or drug screening experiments. Intracellular phosphorylation and dephosphorylation events are enzymatically catalysed by kinases and phosphatases, respectively, and constitute the most important signalling mechanisms known in eukaryotic cells [4]. The phosphorylation state of a protein can determine its activity and, thereby, regulate the pathway(s) in which it is involved. Thus, the present study probed the potential role of key components of host hepatocyte signal transduction pathways, focusing on kinases as key regulators for a wide range of cellular functions.

## Results

### Kinome-wide RNAi screen implicates at least 5 host kinases in *Plasmodium* infection of human hepatoma cells

We have used systematic RNAi screening to selectively silence the expression of 727 genes encoding proteins with known or putative kinase activity, as well as kinase-interacting proteins, thereby covering the entire annotated kinome (Table S1). The effect of each gene-specific knock-down on the infection of Huh7 cells by *Plasmodium* sporozoites was then monitored using the high-throughput, high-content immunofluorescence microscopy-based assay mentioned above (Figure 1A). Briefly, short interfering RNA duplexes (siRNAs) targeting each of the chosen genes were transfected into Huh7 cells 24 h after seeding in 96-well plates. Forty-eight h later, cells were infected with *P. berghei* sporozoites. Cells were fixed 24 h after infection and immuno-stained to detect intracellular parasites (EEFs), as well as host cell nuclei and F-actin to estimate cell numbers and confluency, respectively. Following image acquisition, customized image analysis algorithms were used to automatically quantify infection rates, normalizing the number of EEFs against the cell confluency in each well. A plate-wise normalization was also used to facilitate comparisons between plates in the first pass of the screen, where the low rate of positive hits yields minimal expectation of variability in the mean infection values between different plates. To this end, the infection rate in each experimental well was calculated as a percentage of the mean infection rate from all experimental wells on that plate. In order to assess possible siRNA effects on cell proliferation, infection rate data were plotted against the number of nuclei, also expressed as a percentage of the mean number of nuclei for that plate.

The RNAi strategy employed was validated by targeting 53 randomly chosen genes with 3 siRNAs each and performing quantitative real-time PCR (qRT-PCR) analysis to determine the level of knock-down achieved in each case. For 13 of these genes either expression was too low to be correctly assessed or primer specificity was insufficient. Most importantly, for 85% of the genes whose expression could be determined, at least 1 of the siRNAs led to an expression knock-down greater than 70% (Figure 1B).

**Figure 2:**
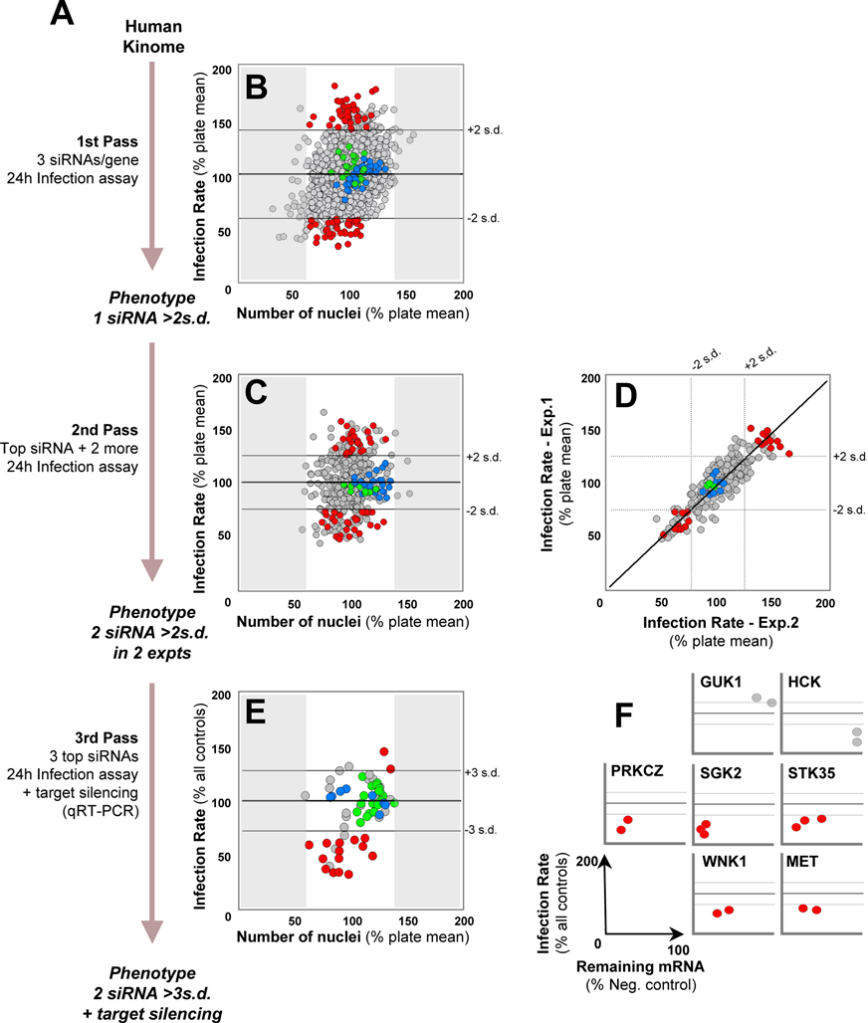
A kinome-wide RNAi screen identifies host genes that influence *P. berghei* sporozoite infection of Huh7 cells. (A) Schematic illustration of the three screening passes with increasing stringency criteria. (B) Plot of pass 1 of the RNAi screen representing the effect of 2181 siRNAs targeting 727 human genes on Huh7 cell infection by *P. berghei* sporozoites and cell nuclei count. Infection rates for each experimental condition were normalized against cell confluency. The horizontal lines represent 100%±2.0 s.d. of the average of all infection data in the assay. Each circle represents one siRNA (mean of triplicate values). Negative controls appear as blue and green circles. corresponding to untreated cells and cells transfected with a non-specific control siRNA. respectively. Red circles highlight the siRNAs targeting the 73 candidate genes selected to undergo a second screening pass. The shaded areas correspond to cell numbers outside the ±40% interval centred on the average number of nuclei for the whole dataset. (C) Plot of 2 independent runs of pass 2 of the RNAi screen representing the effect of 227 siRNAs targeting 73 human genes on Huh7 cell infection by *P. berghei* sporozoites and cell nuclei count. Shading and colour attributions are the same as in panel (B). with red circles representing the siRNAs targeting the 16 genes selected to undergo a third screening pass. The horizontal lines represent 100%±2.0 s.d. of the average of all the negative controls in the assay. (D) Plot comparison of the 2 runs of pass 2 of the RNAi screen. Colour attributions are the same as in panels (B. C). The comparison reveals a high correlation (R = 0.88) between the duplicate runs of pass 2 of the screen (diagonal line). The horizontal and vertical lines represent 100%±2.0 s.d. of the average of all the negative controls in the assay. (E) Plot of pass 3 of the RNAi screen representing the effect of 37 siRNAs targeting 16 human genes on Huh7 cell infection by *P. berghei* sporozoites and cell nuclei count. Remaining mRNA levels following RNAi were determined for each of these genes by qRT-PCR (see text and Figure 2F). Colour attributions and shading are the same as in (B. C. D). Red circles highlight siRNAs targeting the genes for which at least two independent siRNAs led to an infection increase or decrease above or below ±3.0 s.d. of the average of all the negative controls in the assay. respectively. The horizontal lines represent 100%±3.0 s.d. of the average of all the negative controls in the assay. (F) Effect of siRNA on infection rates *versus* remaining mRNA levels for the 7 genes targeted by the siRNAs highlighted in red in (E). Each circle represents one siRNA (mean of triplicate values). For all genes except GUK1 and HCK. represented in light grey. a positive correlation between infection rate and remaining gene-specific mRNA levels is observed. Shading attributions are the same as in (B. C. E). The horizontal lines represent the same as in E (100%±3.0 s.d. of the average of all the negative controls in the assay). The axes on the bottom left of the panel denote the scale of each of the plots in the panel.

Sporozoite infection assays inevitably have considerable levels of variation, a problem that cannot currently be overcome and which exacerbates difficulties generally associated with siRNA screens. In order to reduce the risk of reporting false positives, a multi step screening system was devised in which candidate genes were subjected to three screening passes with increasingly stringent selection criteria (Figure 2A). In the first pass, the 727 selected genes were screened by targeting each with three distinct siRNAs used individually (Table S1). In order to minimize the number of false negative results, candidate gene hits were selected for follow-up in pass 2 if any single one of the three siRNAs yielded an increase or decrease on infection greater than 2 standard deviations (s.d.) of the average of the infection of the whole data set, within a defined range of nuclei number (±40% of the average number of nuclei in each experimental plate) (Figure 2B). The latter precaution, while relatively inclusive, allowed us to exclude from further analysis those siRNAs yielding strong effects on cell proliferation or survival.

As a result, 73 genes were selected to undergo a second pass of confirmation screening, in which up to 2 additional siRNAs were included to maximize the detection sensitivity for those genes that had yielded only a single siRNA hit in pass 1 (Table S1). In this round of analysis, siRNAs were noted as “positive candidates” if they yielded infection rates more than 2 s.d. above or below the mean of all the negative controls in this pass. Negative controls replaced whole data set mean for s.d. calculation, since the selected subset of genes in this pass 2 was expected to have a significantly higher hit rate than in pass 1. To minimize the risk of false positives due to siRNA sequence-dependent off-target effects, the selection of candidate genes for follow-up beyond pass 2 required that at least two independent siRNAs targeting the same gene be “positive candidates” according to the above selection criteria (Figure 2C). Furthermore, genes for which different siRNAs yielded conflicting phenotypic results were also excluded from further analysis. In order to further minimize any bias due to experimental variability, all pass 2 siRNAs were assayed in two independent experiments, and were selected for follow-up only if the criteria were met in both experiments (Figure 2D). It is worthwhile noting that while in Pass 1 only 3.6% of the siRNAs met the selection threshold, 18.4% of the siRNAs tested for the first time in Pass 2 met similar criteria, while the distribution of infection levels in controls is not statistically different between pass 1 and pass 2 experiments, showing that a 5-fold enrichment has taken place from Pass 1 to Pass 2.

The 16 genes thus selected for further verification in pass 3 were targeted with the siRNAs yielding the strongest phenotypes in the second pass. This third pass was used to further restrict our selection to those genes showing clearest functionality, i.e. those with at least two siRNAs yielding infection rates more than 3 s.d. above or below the mean of all the negative controls in the assay, respectively (Figure 2E). Secondly, target mRNA knock-down levels attained for these genes were also assessed in this pass by qRT-PCR. This allowed the selection of positive hit candidates to be refined further yet by excluding genes for which a correlation between phenotypic severity and decreased mRNA levels could not be confirmed (Figure 2F, Table 1).

**Table 1:** List of genes in Pass 3 of the RNAi screen.

Gene name	NCBI Gene Accession Number	NCBI ID for Targeted Transcripts	Kinome Group	Main Described Functions	siRNA ID from Supplier^1^	Cell Proliferation^2^ (Pass3)	Infection Rate^3^ (Pass3)	Remaining mRNA^4^ (Pass3)	Infection Rate^5^ (Pass2)	Infection Rate^5^ (Pass3)
BRD3	8019	NM_007371	Atypical	Unknown	111249	80.7	119.2	16.0	141.5	115.5
					242412	79.8	111.8	21.3		
C9orf12	64768	NM_022755	Non-PK	Unknown	1186	93.7	68.2	34.3	135.3	92.1
					1281	135.7	101.3	77.9		
					242460	124.2	106.7	26.0		
CDC2L1	984	NM_001787, NM_033486/87/88/89/90/92/93	CMGC	Cell growth and survival; Progression through cell cycle; Transcription regulation	41656	121.8	103.6	43.3	124.1	92.9
					214537	130.9	90.3	68.3		
					214538	125.5	84.9	70.7		
CDKN1B	1027	NM_004064	Not kinase	Cell cycle progression; Proliferation; Control of actin cytoskeleton; Motility	118712	115.7	119.9	80.3	63.7	102.7
					242378	95.2	85.5	42.9		
EPHA3	2042	NM_005233, NM_182644	TK	Cell proliferation; Vesicle trafficking	103330	85.4	56.0	n.d.	55.6	65.8
					103414	93.4	75.7	n.d.		
GUK1	2987	NM_000858	Non-PK	Unknown	71	135.9	129.5	104.0	146.2	137.5
					72	129.8	145.5	86.3		
HCK	3055	NM_002110	TK	Apoptosis; Cell adhesion	205	88.8	53.9	n.d.	43.1	43.9
					207	83.6	33.9	n.d.		
MARK2	2011	NM_004954, NM_017490	CAMK	Cell polarity; Microtuble organisation	103359	80.1	39.8	n.d.	72.2	79.9
					103443	120.0	120.0	n.d.		
MET	4233	NM_000245	TK	Cell growth and proliferation	242542	61.2	59.3	44.1	64.3	60.2
					242543	77.5	61.1	27.4		
NJMU-R1	64149	NM_022344	Not kinase	Unknown	140706	57.5	104.9	18.5	147.5	96.9
					242458	95.4	88.9	17.4		
PRKCI	5584	NM_2740	AGC	Cell growth and survival; cytoskeleton organisation	311	116.5	124.1	15.4	139.0	125.9
					242360	89.2	127.8	20.7		
PRKCZ	5590	NM_002744	AGC	Cell growth and survival; cytoskeleton organisation	103575	88.6	34.4	18.0	76.9	50.0
					242362	112.6	65.5	28.8		
PRKWNK1	65125	NM_018979	Other Group	Regulation of salt transport; cell growth	1269	110.5	58.1	46.2	71.7	52.4
					242450	74.0	46.7	30.2		
SCGB2A1	4246	NM_002407	Not kinase	Unknown	143539	126.1	113.1	n.d.	140.8	113.6
					242352	97.6	131.6	n.d.		
					242353	108.6	96.2	n.d.		
SGK2	10110	NM_016276, NM_170693	AGC	Regulation of transport; apoptosis	1485	97.3	32.2	15.9	59.1	39.6
					1579	119.5	49.3	18.5		
					1669	76.4	37.3	12.7		
STK35	140901	NM_080363	Other Group	Regulation of actin stress fibers	1135	88.5	47.1	16.4	61.5	57.5
					103377	89.2	61.4	25.4		
					103461	102.8	64.0	48.6		

1 Ambion, Applied Biosystems.

2 Number of cell nuclei, shown as % of plate mean.

3 Number of EEFs normalised to confluency, shown as % of plate mean.

4 % relative to negative control.

5 Average of infection rates for the selected siRNAs.

n.d. – not determined.

Based on these data, the following 5 genes have emerged from our screen as the clearest and strongest positive hits, showing RNAi-induced loss-of-function phenotypes with specific, reproducible and marked effects on *P. berghei* infection rates in our Huh7-based assay: MET, PKCζ (PKCzeta), PRKWNK1, SGK2 and STK35. As illustrated in Figure S1, knock-down of the expression of these genes did not lead to any significant effects in terms of cell proliferation or morphology (see also Table 1). It should also be noted that the present data do not rule out the possible involvement of other genes among those tested here, since negative results in RNAi screens are generally inconclusive [5], and certain genes showing phenotypes with lower than 3 s.d. from mean levels in our assays may provide real, though perhaps more subtle, functionalities in this context. For this reason, the reader is referred to Table S1 for a comprehensive list of siRNAs employed throughout the screen and their corresponding z scores (which measure the number of standard deviations away from the mean for the whole normalized data set), obtained throughout the screen.

### Top screening hits classification

It may be noted that all five genes we identified as top hits encode protein kinases belonging to 3 different classes, according to the kinome classification [6]: “AGC”, “other” and “TK” (Figure S2A). While it is tempting to draw conclusions from this, we would advise against it since, as aforementioned, the experimental methods and prioritization strategies used here cannot conclusively rule out the involvement of other tested genes which did not make the final selection. The range of cellular processes implicated by the genes identified as top hits in the present screen include cell cycle control, cytoskeleton regulation, osmotic balance and stress/immune responses (Figure S2B). This is consistent with the broad range of cellular functions similarly implicated by Agaisse *et al.* [7] in the infection of *Drosophila* cells by intracellular bacterial pathogens. However, when we performed an hypergeometric test to identify which Gene Ontology (GO) terms were significantly enriched in the analysis (*p*<0.05), not all categories were equally represented in passes 2 and 3 with a major proportion of genes involved in cytoskeleton regulation. Interestingly, amongst the five final hits, we could observe a complete shift of representation, as a 50/50 segregation was observed for genes related to stress/immune responses and to cell cycle control (Figure S2C).

### PKCζ inhibition leads to a decrease in host cell infection by *Plasmodium* sporozoites

In order to further characterize the functionalities identified in our cell-based infection model and to validate their relevance both from a physiological point of view and in terms of human malaria, we have initiated detailed follow-up studies for all 5 of the top hits from our RNAi screen, and present herein results for PKCζ, the first of these to be prioritized due to its role in several liver pathological processes [8],[9]. PKCζ is part of the large family of PKCs, which has been implicated in a wide range of cellular processes. PKC isotypes include 10–15 members, divided into 4 groups [10]. One of these groups, known as the atypical PKCs (aPKCs) [11], comprises the PKCζ [12] and PKCλ/ι (PKC lambda/iota ) [13] isoforms. The aPKCs have been implicated in numerous processes, including cell growth and survival, regulation of NF-κB (NF-kappaB) activation and polarity (reviewed in [11],[14],[15]).

**Figure 3:**
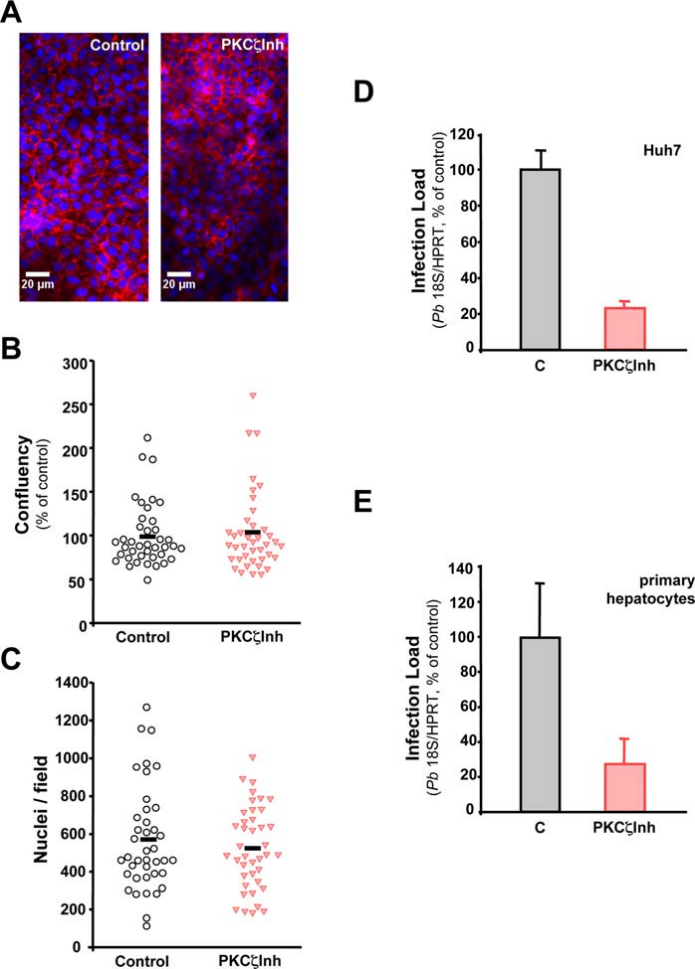
PKCζ inhibition by a pseudosubstrate decreases hepatocyte infection without affecting host cell viability. (A) Representative pictures of cells treated with the PKCζ pseudosubstrate inhibitor and a control peptide. The pictures depict nuclei (in blue) and actin (in red) and show that cells are not affected by the inhibitor peptide. (B. C) Quantification of cell confluency (B) and number of nuclei (C) in 40 microscope fields of cells treated with the PKCζ pseudosubstrate inhibitor and a control peptide. (D. E) Effect of PKCζInh (20 µM) on *P. berghei* load in Huh7 cells (D) and mouse primary hepatocytes (E). Parasite loads were measured by qRT-PCR 24 h or 48 h after sporozoite addition. respectively. Results are expressed as the mean±s.d. of triplicate samples. Cells treated with a myristoylated scrambled peptide were used as controls in each experiment. Infection loads are normalized to the corresponding control infection levels (100%).

All PKC isoenzymes have an autoinhibitory pseudosubstrate domain sequence that can bind to the substrate-binding cavity and prevent catalysis [16]. This inhibtory effect can be mimicked *in vitro* by addition of a corresponding synthetic peptide [17]. Thus, we used the cell-based assay described above for our screen to test the effects of a myristoylated PKCζ pseudosubstrate (myr-SIYRRGARRWRKLYRAN), previously characterized as a specific PKCζ inhibitor (PKCζInh) [18],[19], on *P. berghei* infection. Further data on the specificity of PKCζInh is shown in Figure S3. A scrambled myristolated peptide was used as control in all PKCζ inhibition experiments [18]. Treatment of cells with PKCζInh had no obvious effects on nuclear or cell morphology and as well as on the cell number and confluency (Figure 3A–C), as previously observed for cells transfected with siRNA oligonucleotides targeting PKCζ (Figure S1, Table 1). Still, treatment of cells with PKCζInh had a significant effect in the level of cell infection by *P. berghei* sporozoites, as quantified using qRT-PCR-based measurements of *Plasmodium* 18S rRNA levels found within Huh7 cells (Figure 3D) and mouse primary hepatocyte extracts (Figure 3E) harvested 24 and 48 h after *P. berghei* sporozoite addition, respectively. Our results show that a 20 µM concentration of PKCζInh leads to a ∼80% and ∼60% reduction in *P. berghei* infection rates in Huh7 hepatoma cells and primary hepatocytes, respectively (Figure 3D and E; *p*<0.01 and *p*<0.05), offering a RNAi-independent confirmation of our present findings on the role of PKCζ in *P. berghei* infection.

### Inhibition of PKCζ impairs invasion of host cells by *Plasmodium* sporozoites

**Figure 4:**
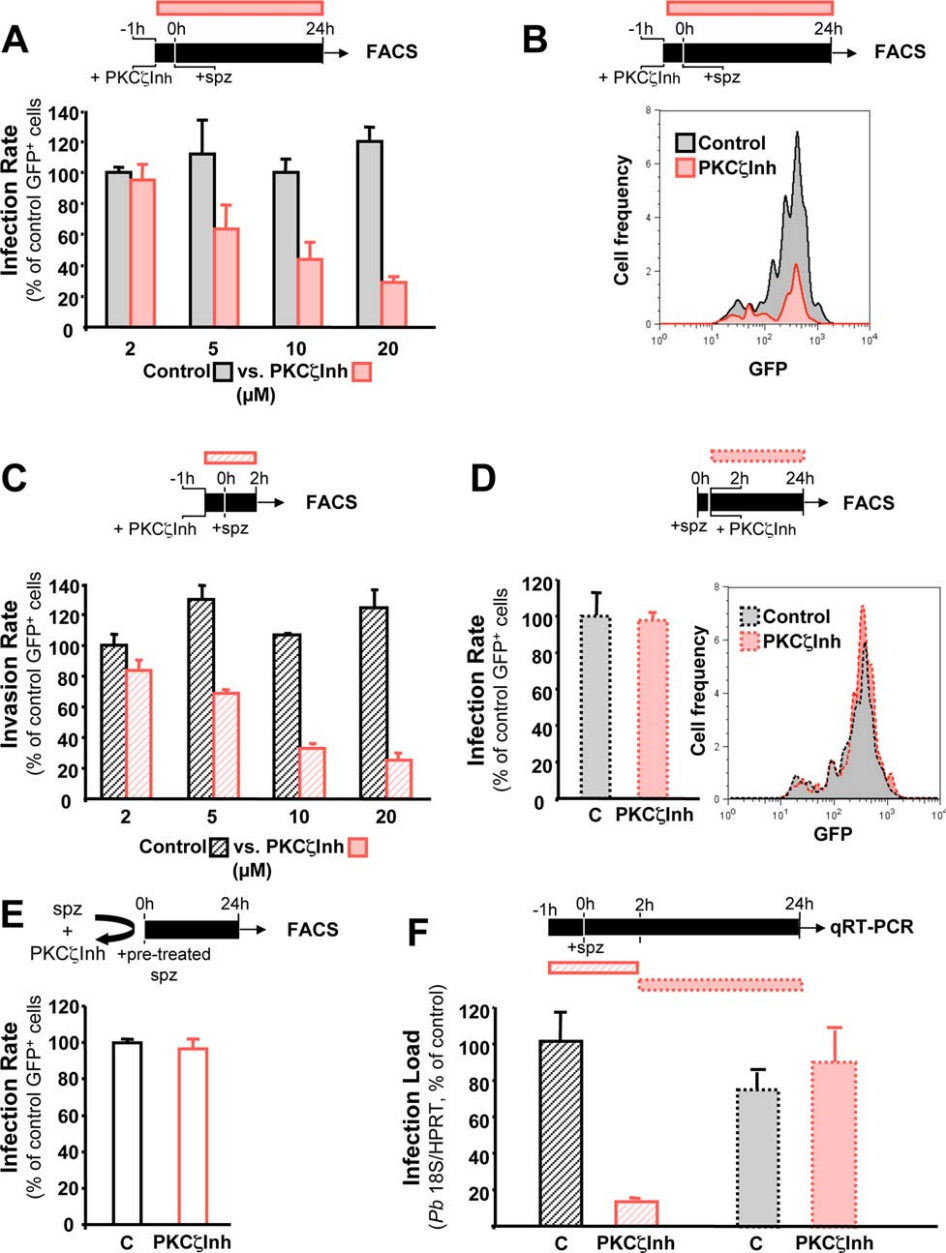
Inhibition of PKCζ impairs invasion of host cells by *Plasmodium* sporozoites. (A) PKCζ inhibition by PKCζInh decreases *P. berghei* sporozoite infection of Huh7 cells in a dose-dependent manner. PKCζInh was added to Huh7 cells 1 h before addition of GFP-expressing *P. berghei* sporozoites and infection rate was measured 24 h later by FACS. (B) PKCζ inhibition by PKCζInh does not affect EEF development. PKCζInh was added to Huh7 cells 1 h before addition of GFP-expressing *P. berghei* sporozoites and GFP intensity (proportional to EEF development) was measured 24 h later by FACS. (C) PKCζ inhibition by PKCζInh decreases *P. berghei* sporozoite invasion of Huh7 cells in a dose-dependent manner. PKCζInh was added to Huh7 cells 1 h before addition of GFP-expressing *P. berghei* sporozoites and infection rate was measured 2 h later. by FACS. (D) PKCζ inhibition does not affect infection after invasion has occurred. PKCζInh was added to Huh7 cells 2 h after addition of GFP-expressing *P. berghei* sporozoites and infection rate was measured 24 h later. by FACS. (E) PKCζInh does not affect infection by acting on sporozoites directly. Sporozoites were pre-treated with PKCζInh for 1 hour before addition to the cells and infection rate was measured 24 h later by FACS. All results are expressed as the mean±s.d. of GFP^+^ cells (%) in 3 independent infections. (F) PKCζ inhibition during the period of cell invasion by sporozoites. but not during their intracellular development period. leads to a decrease in infection. The infection rate was determined by qRT-PCR in Huh7 cells incubated with PKCζInh throughout different periods of the infection process. namely −1 to 2 h and 2 h to 24 h relative to sporozoite addition.

In order to gain a better insight on the possible role of PKCζ in the infection process, the effects of PKCζInh on different periods of hepatocyte infection were examined by flourescence activated cell sorting (FACS) analysis of host cells infected with GFP-expressing *P. berghei* parasites, measuring the proportion of GFP^+^ cells [20]. Indeed, FACS analysis of cells infected with GFP-expressing parasites enables discerning whether the observed effect on infection is due to a decrease in the number of infected cells or to an impairment of *Plasmodium* development inside host cells [20]. Treatment of Huh7 cells with PKCζInh 1 h prior to addition of GFP-expressing *P. berghei* sporozoites led to a marked, dose-dependent decrease in infection rate, as measured by the proportion of infected cells relative to control samples 24 h after sporozoite addition (Figure 4A; *p*<0.05 for PKCζInh≥5 µM). Treatment with PKCζInh did not affect *Plasmodium* development, as shown by the similar GFP intensities of treated and control cells (Figure 4B). Next, we sought to determine whether the decrease in the number of infected cells observed at 24 h after sporozoite addition was due to a decrease in invasion rate or to the disappearance of infected cells throughout infection. Since, in the infection assay employed, >95% of invasion events are known to take place within the first 2 h after sporozoite addition [20], any effects on invasion can be quantified by analyzing cells at this timepoint. As shown in Figure 4C, the effect of PKCζInh in cells analyzed 2 h after sporozoite addition is closely comparable to that seen with the full 24 h treatment, indicating that PKCζ likely plays a role during host cell invasion by *P. berghei* sporozoites (*p*<0.05 for PKCζInh≥5 µM). In addition, when PKCζInh was added 2 h after sporozoite addition, no significant effect was observed in infection rate measured at 24 h (Figure 4D), not only showing that the effect observed on the early steps of infection is not due to PKCζInh toxicity to host cells but also strengthening the notion that PKCζ influences *Plasmodium* infection by playing a role during cell invasion. Importantly, infection rates were not affected by pre-incubation of *Plasmodium* sporozoites with PKCζInh for 1 hour prior to their addition to hepatoma cells, showing that PKCζInh has no direct effect on sporozoite viability (Figure 4E). Further confirmation of the involvement of PKCζ in sporozoite invasion of Huh7 cells, but not on the parasite's intracellular development was obtained by employing qRT-PCR to quantify infection 24 h after infection of cells incubated with PKCζInh either during the invasion or the development periods, exclusively (Figure 4F). These results show that a marked decrease in intracellular parasite numbers is observed when cells are incubated with the inhibitor during the first 2 hours after sporozoite addition (*p*<0.001), whereas no effect is observed when the compound is added after invasion is completed. Together, these data confirm the physiological relevance of PKCζ, identified in the RNAi screen, and suggest that the latter plays a role during the invasion of hepatoma cells by *P. berghei* sporozoites.

### PKCζ knock-down in mouse livers confirms the physiological relevance of PKCζ role in malaria infection *in vivo*

**Figure 5:**
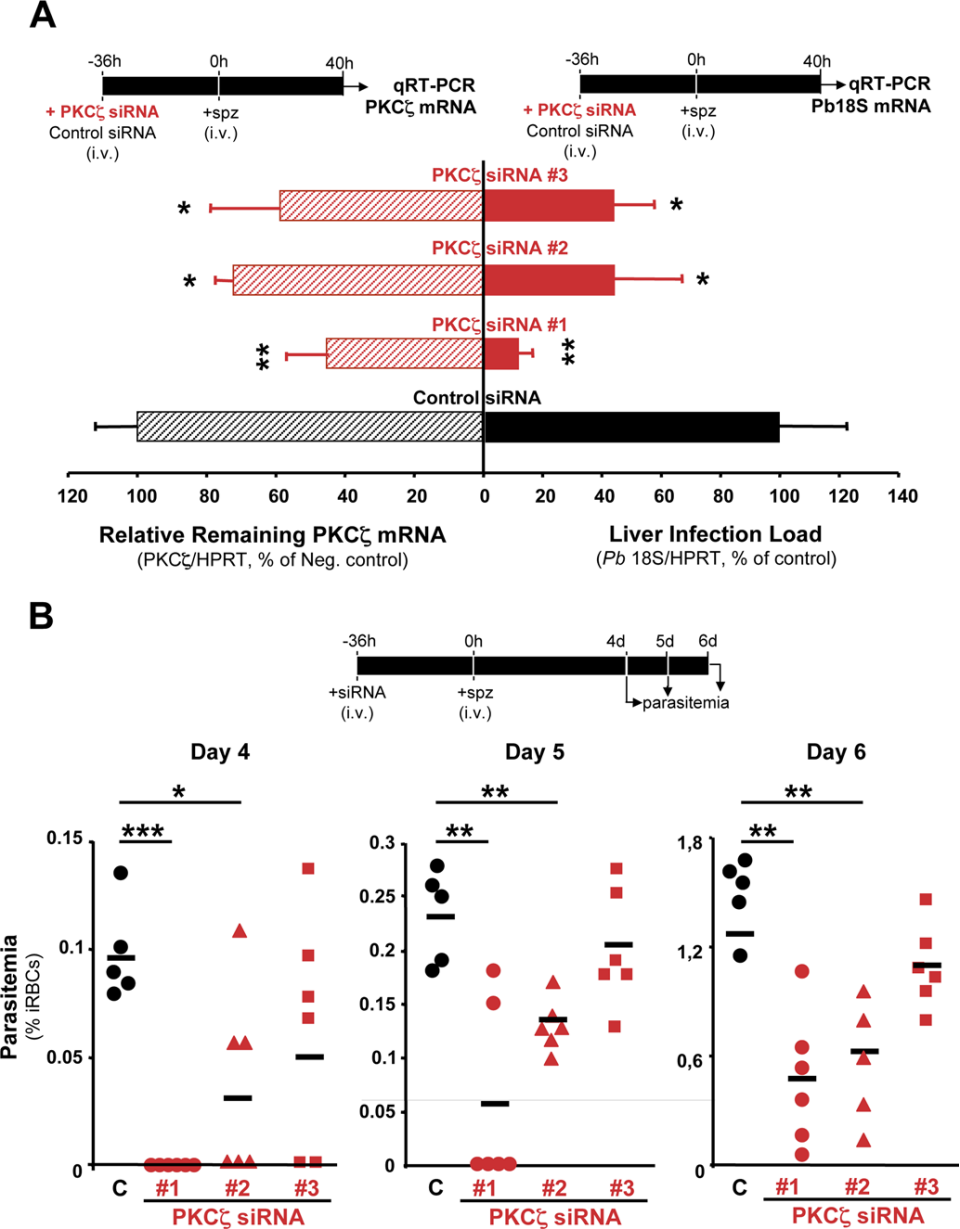
*In vivo* PKCζ down-modulation reduces liver infection by *Plasmodium* sporozoites confirming the physiological relevance of RNAi screen results. (A) Effect of siRNA-mediated *in vivo* silencing of *PKCζ* on mouse liver infection by *P. berghei* (solid bars) and on PKCζ mRNA levels (dashed bars). measured by qRT-PCR analysis of liver extracts taken 40 h after sporozoite i.v. injection. Mice were infected 36 h after RNAi treatment. Results are plotted as the percentage of the mean of negative control samples. “C”. The remaining mRNA levels for PKCζ were measured by qRT-PCR in the same liver samples. Results are expressed as the mean±s.d. of all mice in each group. Black bars represent the negative control (5 mice treated with luciferase-targeting siRNA). Red bars represent mice treated with the 3 independent siRNAs targeting the *PKCζ* gene (6 mice per siRNA). (B) Knock-down of PKCζ expression by RNAi delays the onset of blood stage infection. as measured by parasitemia (percentage of infected red blood cells. iRBC) quantification using flow cytometry. Each symbol represents one mouse. Black circles represent the negative controls (5 mice treated with luciferase-targeting siRNA). Red symbols represent the 6 mice treated with the 3 independent siRNAs targeting the *PKCζ* gene (6 mice per siRNA).

Finally, we tested the *in vivo* physiological relevance of our cell-based findings more thoroughly by using systemically-delivered, liposome-formulated siRNAs designed to specifically silence PKCζ expression in adult mice, and infecting these with *P. berghei* sporozoites. *In vivo* RNAi treatments using the same systemic administration of siRNAs including the same formulation used here have previously been shown to yield potent gene-specific knock-downs in adult mice without major toxicity, nor any detectable disruption of the endogenous microRNA pathway [21]–[23]. In our present experiments, mice from the same litter were given an initial intravenous (i.v.) injection of either test or control siRNAs and, infection was initiated 36 h later by i.v. injection of freshly isolated *P. berghei* sporozoites. Mice were sacrificed 40 h after infection to permit parallel analyses of gene silencing and infection load. In order to address the risk of sequence-dependent off-target effects, three distinct siRNA sequences targeting PKCζ were tested individually, while a siRNA targeting luciferase, a transcript known to be absent in these mice, was used to address sequence-independent off-target effects that may arise from these treatments. Under these conditions, no toxicity was observed (Figure S4) and PKCζ expression was reduced in adult mouse livers when using each of the 3 distinct PKCζ-specific siRNAs, yielding an average of ∼56–73% remaining PKCζ mRNA, as measured by qRT-PCR of liver extracts taken 76 h after siRNA treatment, relative to the controls (Figure 5A; *p*<0.05). This silencing was accompanied, for all three PKCζ-specific siRNAs, by significant reductions in liver infection, yielding an average per siRNA of ∼9–40% of control infection loads, as measured by qRT-PCR of *P. berghei* 18S rRNA in liver extracts taken 76 h after siRNA treatment, as described above (Figure 5A, *p*<0.05). The reductions in liver infection load showed a broad correlation with the level of PKCζ silencing achieved by the siRNAs with siRNA #1, which leads to the most significant reduction in PKCζ, showing the most striking difference in infection (Figure 5A; *p*<0.01). In a further, parallel experiment, semi-quantitative Western blotting analysis of liver extracts taken 76 h after siRNA treatment, from mice injected with the PKCζ siRNA yielding the strongest reduction in liver infection, confirmed that PKCζ expression was significantly reduced at the protein level in these mice (∼55%; Figure S5, *p*<0.01). Additionally, another 3 independent groups of mice treated with the same 3 distinct PKCζ siRNAs showed a decrease in blood parasitaemia (percentage of infected erythrocytes), relative to control mice (Figure 5B). In fact, while by day 4 after sporozoite injection all 5 mice in the control group were positive for blood stages, none of the 6 mice in the group pre-treated with the strongest PKCζ-specific siRNA were (Figure 5B; *p*<0.001). Although less striking, both other siRNAs also led to a delay in the appearance of parasites in the blood and siRNA #2 led to a significant reduction in average blood parasitaemia (Figure 5B; *p*<0.05). Together, these data strongly support the conclusion that PKCζ is a physiologically important host factor needed for the liver stage of *Plasmodium* infection both in cultured cells *in vitro* and in animals *in vivo*.

## Discussion

The approach described here constitutes, to our knowledge, the first report of a genome-scale RNAi screen for key host factors for infection of human cells by a parasite. *Plasmodium* sporozoite infection *in vitro* assays are fraught with specific biological variability issues that enhance the difficulties inherent to any high-throughput RNAi screening assay. Thus, in order to minimize the chance of excluding false negative and identifying false positive results, we have devised a multi-step strategy employing increasingly stringent selection criteria throughout the screen. Nevertheless, we cannot definitively rule out the possibility of, in this process, having discarded candidates that may indeed play a role during infection, but which did not “survive” the criteria employed throughout the three stages of selection. For this reason, we encourage the reader to consult Table S1, where details of the screening process are presented and potentially relevant genes can be identified.

Our systematic analysis of the human kinome in the context of the liver stage of malaria infection has directly implicated at least five host kinases in this process: MET, PRKWNK1, SGK2, STK35 and PKCζ. By doing so through direct functional tests for each of the genes assayed, this dataset establishes clear causal roles in the processes examined and reveals novel key host molecules in these pathways that significantly affect *Plasmodium*'s success in infecting hepatocytes.

Among our top hits, the MET gene, which encodes the hepatocyte growth factor (HGF) receptor, is the only one to have been previously shown to influence *Plasmodium* infection of hepatocytes [24],[25], an effect that has been proposed to occur through inhibition of apoptosis [25]. It has been demonstrated that transfection of hepatoma cells with a dominant-negative form of MET leads to a reduction in *Plasmodium* infection while transfection with a constitutively active form results in an infection increase [24]. Thus, the emergence of MET as one of our top hits whose knock-down consistently led to a decrease in infection represents a validation of the screening methodology used here, and strengthens the predictive value of the other hits. Among those, both SGK2 and PRKWNK1 are serine/threonine kinases that have been implicated in osmotic control through the regulation of Na^+^ and K^+^ transport channels [26]–[29]. Down-modulation of both of these osmotic and oxidative stress-responsive proteins also led to a reduced infection in the present screen. Although their role in *Plasmodium* infection remains unclear, the present data may be highlighting the importance of maintaining an optimal osmotic balance in the host cell to permit successful infection. In addition, it has recently been shown that exposure of sporozoites to the intracellular K^+^ concentration enhances sporozoite infectivity [30]. Whether or not SGK2 or PRKWNK1 act on infection through the control of K^+^ concentration will require further investigation. Concerning STK35, it is known to interact with CLP-36, a PDZ-LIM protein, and re-localize from the nucleus to actin stress fibres [31]. This has led to the suggestion that STK35 may act as a regulator of the actin-myosin cytoskeleton in non-muscle cells [31]. Indeed, there are indications that the reorganization of the host cell actin cytoskeleton may be important for *Plasmodium* infection [24]. Thus, it is appealing to consider the hypothesis that recruitment of STK35 may influence *Plasmodium* infection by playing a role in this process.

Finally, the gene which we have characterized in most detail here, PKCζ, is part of the large family of PKCs that has been implicated in numerous cellular processes. PKC isotypes include 10–15 members, divided into 4 groups [10],[16]. One of these groups, known as the atypical PKCs (aPKCs) [11], comprises the PKCζ [12] and PKCλ/ι (PKCiota/lambda) [13] isoforms. The aPKCs have been implicated in numerous processes, including cell growth and survival, regulation of NF-κB activation and polarity (reviewed in [11],[14],[15]). In the present study, loss of PKCζ function both *in vitro* and *in vivo*, whether by RNAi silencing or by pseudo-substrate inhibition, led to decreased infection rates. This unequivocally establishes a key role of PKCζ in host liver malarial infection, thereby, giving confidence that the other genes identified in the RNAi screen also play a relevant role during infection. Furthermore, as we gain additional understanding of the pathway defined by PKCζ in this context, its potential biomedical value may develop not merely as one, but rather also as the founding member in an entirely novel class of anti-malarial drug target. In addition, our data reveals a role for PKCζ signaling in host cell invasion by *Plasmodium* sporozoites. To our knowledge, this is the first host cell signaling molecule to be identified as an important player in *Plasmodium* invasion and paves the way to a better understanding of this essential host-*Plasmodium* interaction in the establishment of a malaria infection.

The interaction of a pathogen with its host cell activates intracellular signaling cascades that regulate innate immune responses and govern the outcome of infection (see [32] for a recent review on this topic). These signaling pathways may also be exploited by the pathogen to its own benefit as was recently suggested for the interaction of hepatitis C virus, another major liver pathogen, with its target cells [33]. While our present study does not demonstrate any active exploitation or modulation of host signal transduction pathways by *Plasmodium*, it does reveal novel key host molecules in these pathways that significantly affect the parasite's success in infecting hepatocytes. Our efforts are thus starting to yield crucial molecular details needed to build a coherent picture of the key cellular events taking place during the liver phase of malaria infection.

Altogether, these results contribute toward a better understanding of host-pathogen interactions, which may help in accelerating the rational design of prophylactic, therapeutic and/or diagnostic strategies aimed to control malaria.

## Methods

### Cells, Mice and Parasites

Huh7 cells, a human hepatoma cell line, were cultured in RPMI medium supplemented with 10% fetal calf serum (FCS, Gibco/Invitrogen), 1% non-essential amino acid (Gibco/Invitrogen), 1% penicillin/streptomycin (pen/strep, Gibco/Invitrogen), 1% glutamine (Gibco/Invitrogen) and 1% HEPES, pH 7 (Gibco/Invitrogen) and maintained at 37°C with 5% CO_2_.

Mouse primary hepatocytes were obtained as previously described [34]. Briefly, they were isolated by perfusion of mouse liver lobule with liver perfusion medium (Gibco/Invitrogen) and purified using a 1.12 g/ml; 1.08 g/ml and 1.06 g/ml Percoll gradient. Cells were cultured in William's E medium containing 4% FCS, 1% pen/strep, 50 mg/ml epidermal growth factor (EGF), 10 µg/ml transferrin, 1 µg/ml insulin and 3.5 µM hydrocortisone in 24 well plates coated with 0,2% Gelatine in PBS. Cells were maintained in culture at 37°C and 5% CO_2_.

C57BL/6 mice, were bred in the pathogen-free facilities of the Instituto de Gulbenkian de Ciência (IGC) and housed in the pathogen-free facilities of the Instituto de Medicina Molecular (IMM). All protocols were approved by the Animal Care Committees of both Institutes.

Green fluorescent protein (GFP) expressing *P. berghei* (parasite line 259cl2) sporozoites [35] were obtained from dissection of infected female *Anopheles stephensi* mosquito salivary glands.

### siRNA design, siRNA library and screening controls

All siRNAs were purchased from Ambion's *Silencer* genome wide library (Ambion/Applied Biosystems, Austin USA). Each gene was targeted by using distinct siRNAs used individually in all cases. Negative control samples included untransfected cells, and cells transfected with a negative control siRNA not targeting any annotated genes in the human genome. A full list of gene names, siRNA ID numbers and sequences, and associated screening data are shown in Table S1.

### High-throughput siRNA screening of *Plasmodium* infection

Huh7 cells (4500 per well) were seeded in 100 µl complete RPMI medium in optical 96-well plates (Costar) and incubated at 37°C in 5% CO_2_. Twenty-four h after seeding, cells were transfected with individual siRNAs in a final concentration of 100 nM per lipofection. Each siRNA was transfected in triplicate. Briefly, for each well, cell supernatant was replaced by 80 µl of serum-free culture medium without antibiotics. One µl of 10 µM siRNA diluted in 16 µl of Opti-MEM (Invitrogen) was complexed with 0.4 µl Oligofectamine (Invitrogen) diluted with 2.6 µl Opti-MEM and added onto the cells following the manufacturer′s protocol. Four h after addition of the complex, 50 µl of fresh RPMI medium, supplemented with 30% FCS, 3% pen/strep, 3% non-essential amino acid, 3% glutamine and 3% HEPES were added to the cells. Two d after siRNA transfection, cells were infected with 10^4^ *P. berghei* sporozoites/well. Twenty-four h after infection, cells were fixed with 4% paraformaldehyde (PFA) in PBS and permeabilized with 0.2% saponin in PBS. Cell nuclei were stained with Hoechst-33342 (Molecular Probes/Invitrogen), filamentous actin was stained with Phalloidin AlexaFluor488 (Molecular Probes/Invitrogen), EEFs were detected using the mouse monoclonal antibody 2E6 and an AlexaFluor555 labeled goat anti-mouse secondary antibody (Molecular Probes/Invitrogen).

### Automated image acquisition and analysis

Plates were acquired with a Discovery1 automated fluorescence microscope (Molecular Devices Corporation, CA, USA) using a 10× lens. In each well, cell nuclei, actin and EEFs were imaged in 9 fields covering a total area of 2.7×2.0 mm. Image data was analyzed using a custom MetaMorph (Molecular Devices Corporation, CA, USA) based algorithm extracting the following values for each imaged field: cell proliferation as measured by the number of nuclei per imaged field (Hoechst staining), cell confluency as measured by the percentage of the imaged field covered by actin staining and number of EEFs as number of compact, high contrast objects in a size range from 16 to 150 µm^2^. Within each field, the number of EEFs was normalized to the cell confluency. Normalized EEF numbers and number of nuclei were averaged between the 9 imaged fields within each well. Mean and standard deviations were calculated for each experimental triplicate.

### Gene-specific expression and infection quantification by qRT-PCR

For gene-specific expression *in vitro*, total RNA was isolated from Huh7 cells 48 h post-transfection (Invitek Invisorb 96-well plate kit) and converted into cDNA (ABI's HighCapacity cDNA reagents) with random hexamers, following the manufacturer recommendations. qRT-PCR used the SybrGreen method with Quantace qPCR mastermix at 11 µl total reaction volume, containing 500 nM of the target-specific primers, and primers that were designed to specifically amplify a fragment of the selected genes. Real-time PCR reactions were performed on an ABI Prism 7900HT system. Relative amounts of remaining mRNA levels of RNAi targets were calculated against the level of RPL13A or 18S rRNA, as housekeeping genes. Remaining mRNA levels of RNAi-treated samples were compared with those of samples transfected with Negative unspecific siRNA. RPL13A-specific primer sequences were: 5′-CCT GGA GGA GAA GAG GAA AGA GA-3′ and 5′-TTG AGG ACC TCT GTG TAT TTG TCA A-3′. 18S rRNA-specific primer sequences were: 5′-CGG CTT AAT TTG ACT CAA CAC G-3′ and 5′-TTA GCA TGC CAG AGT CTC GTT C-3′.

For infection determination *in vivo* or *ex vivo*, total RNA was isolated from livers or primary hepatocytes using Qiagen's RNeasy Mini or Micro kits, respectively, following the manufacturer's instructions. The determination of liver parasite load *in vivo*, was performed according to the method developed for *P. yoelii* infections [36]. Livers were collected and homogenized in denaturing solution (4 M guanidine thiocyanate; 25 mM sodium citrate pH 7, 0.5% sarcosyl and 0.7% β-Mercaptoethanol in DEPC-treated water), 40 h after sporozoite injection. Total RNA was extracted using Qiagen's RNeasy Mini kit, following the manufacturer's instructions. RNA for infection measurements was converted into cDNA using Roche's Transcriptor First Strand cDNA Synthesis kit, according to the manufacturer's protocol. The qRT-PCR reactions used Applied Biosystems' Power SYBR Green PCR Master Mix and were performed according to the maunufacturer's instructions on an ABI Prism 7000 system (Applied Biosystems). Amplification reactions were carried out in a total reaction volume of 25 µl, containing 0,8 pmoles/µl or 0,16 pmoles/µl of the PbA 18 S- or housekeeping gene-specific primers, respectively. Relative amounts of PbA mRNA were calculated against the Hypoxanthine Guanine Phosphoribosyltransferase (HPRT) housekeeping gene. PbA 18 S-, mouse and human HPRT-specific primer sequences were 5′- AAG CAT TAA ATA AAG CGA ATA CAT CCT TAC – 3′ and 5′ - GGA GAT TGG TTT TGA CGT TTA TGT G – 3′ and 5′ – TGC TCG AGA TGT GAT GAA GG – 3′ and 5′ – TCC CCT GTT GAC TGG TCA TT – 3′ and 5′ – TGC TCG AGA TGT GAT GAA GG – 3′ and 5′ – TCC CCT GTT GAC TGG TCA TT – 3′, respectively. For PKCζ mRNA level determination by qRT-PCT, PKCζ-specific primers were used (RT2 qPCR Primer Assay for Mouse Prkcz, SuperArray Bioscience Corporation).

### Pseudosubstrate inhibition of PKCζ

Inhibition of PKCζ was carried out by incubation of the cells with a myristoylated PKCζ peptide (myr-SIYRRGARRWRKLYRAN), whose sequence corresponds to that of a pseudosubstrate inhibitor of the enzyme. A myristoylated scrambled peptide (myr- RLRYRNKRIWRSAYAGR) was used as a control in these experiments.

In order to determine the specificity of the pseudosubstrate inhibitor, Huh7 cells were incubated overnight with either scrambled or pseudosubstrate peptides and then harvested in modified RIPA buffer (150 mM NaCl; 50 mM Tris, pH 7.5; 1% Triton X100; 50 mM NaF; 1 mM Na_3_VO_4_; complete EDTA-free protease inhibitor cocktail). After migration on a 10% Tris-glycine gel, proteins were transferred to a nitrocellulose membrane (BIO-RAD), which was probed with anti-phospho-PKC (pan) (βII Ser660) (Cell Signaling Technology) or anti-phospho-aPKC (Thr555/PKCι; Thr560/PKCζ) (Upstate) plus HRP-conjugated anti-rabbit (Amersham). The membrane was developed with the SuperSignal West Pico Chemiluminescent Subtrate (Pierce).

To further evaluate the specificity of the pseudosubstrate inhibitor towards PKCζ *versus* PKCι, Huh7 cells were transfected (Lipofectamine 2000, Invitrogen) with plasmids encoding GFP-PKCζ or GST-PKCι . Forty eight hours after transfection the cells were incubated with either scrambled or pseudosubstrate peptides for 1 hour and then harvested as before. The relative expression levels of GFP-PKCζ and GST-PKCι were determined by probing the membrane with anti-aPKCζ (C20, Santa Cruz Biotechnology), which recognizes the two isoenzymes. The % of inhibition of PKCζ *versus* PKCι was calculated from the anti-phospho-aPKC signals. All signals were normalized to those of actin.

### Fluorescence Activated Cell Sorting (FACS) analysis

FACS analysis at 2 and 24 after sporozoite addition was performed to determine the percentage of parasite-containing cells and parasite-GFP intensity within infected cells. For infection level measurement at 2 h, 1 mg/ml Dextran tetramethylrhodamine 10,000 MW, lysine fixable (fluoro-ruby) (Molecular Probes/ Invitrogen) was added to the cells immediately prior to sporozoite addition. Cell samples for FACS analysis were processed as previously described [20].

### *In vivo* RNAi

C57Bl/6 mice (male, 6–8 weeks) were treated with a single intravenous (i.v.) administration of 5 mg/kg of siRNA formulated in liposomal nanoparticles (Alnylam). Three different modified siRNAs targeting PKCζ were used: siRNA#1 – 5′-GGGAcAGcAAcAAcuGcuudTsdT-3′; siRNA#2 – 5′-GGccucAcAcGucuuAAAAdTsdT-3′; siRNA#3 – 5′-cccuuAAcuAcAGcAuAuGdTsdT-3. A modified siRNA targeting luciferase was used as control (5′- cuuAcGcuGAGuAcuucGATsT-3′). Lower case letters represent 2'OMe nucleotides and “s” represents phosphorothioate linkage. Thirty-six h after siRNA administration mice were infected by i.v. injection of 2×10^4^ *P. berghei* sporozoites. Remaining PKCζ mRNA levels, parasite load in the livers of infected mice were determined by qRT-PCR 40 h after sporozoite injection, 76 h after siRNA administration. Infection of mice treated with one PKCζ siRNA was allowed to proceed onto the blood stage and parasitemia (% of infected red blood cells) was measured daily. The PKCζ protein level in the liver of siRNA-treated mice was determined by Western blot.

### Quantification of host PKCζ protein expression in the liver

PKCζ protein level in the liver of mice treated with a PKCζ siRNA was quantified by Western blot using the primary antibody (rabbit anti-PKCζ (C20): sc-216, Santa Cruz Biotechnology) and normalised against actin level detected using rabbit anti-actin (A2066, Sigma). Anti-rabbit horseradish peroxidase-conjugated (NA934V, GE Healthcare, UK Ltd.) was used as secondary antibody. The membrane was developed using the ECL Western Blotting Analysis System, according to the manufacturer's instructions (Amersham Bioscience, Germany). Signal quantification was performed using the ImageJ software package (NIH, USA).

### Statistical analysis

For samples in which n>5, statistical analyses were performed using unpaired Student *t* or ANOVA parametric tests. Normal distributions were confirmed using the Kolmogorov-Smirnov test. For samples in which n<5, statistical analyses were performed using Kruskall-Wallis or Wilcoxon non-parametric tests. *p*<0.05 was considered significant, *p*<0.001 was considered highly significant.

## Supporting Information

Table S1 List of siRNAs used throughout the RNAi screen. siRNAs that led to an increase or a decrease in infection are marked in red or in green, respectively. CN denotes the normalised number of nuclei in each condition. Genes in italics are those for which one siRNA met the selection criteria but all three siRNAs used in Pass 1 gave a statistically significant (p<0,05) difference in terms of infection rate. For these genes, the three siRNAs used in Pass 1 were used again in Pass 2.
(0.07 MB PDF)

Figure S1 Infected cells following knock-down of hit genes identified in the RNAi screen. Representative pictures of cells transfected with siRNAs targeting MET, PRKWNK1, SGK2, STK35, PKCζ and a Negative control siRNA, 24 hours after infection with *P. berghei* sporozoites. The pictures depict nuclei (in blue), actin (in red) and EEFs (in green) and show that cell confluency and morphology are not affected by gene knock-down whereas infection is decreased in all cases.
(0.68 MB PDF)

Figure S2 RNAi screen data analysed in terms of gene classification and gene ontology. (A) Data analysis according to distribution through kinase and kinase-related families. (B) Data analysis according to gene ontology and molecular function. (C) Gene ontology enrichment analysis of 3 RNAi screens showing only significantly enriched kinases (p<0.05). Spheres on Pass 3 pie plots denote the 5 kinases identified in the RNAi screen.
(0.13 MB PDF)

Figure S3 Specificity of inhibition by PKCzInh. (A) Specificity of inhibition of atypical PKCs versus conventional and novel PKCs by PKCzInh. Cells treated with PKCzInh or with an equivalent amount of a scrambled peptide control, were probed with an antibody that specifically recognises the autophosphorilated form of atypical PKCs (Thr560 on PKCζ; Thr555 on PKCι) (top). In parallel, treated cells were incubated with phospho-PKC (pan), an antibody that specifically recognises the autophosphorilated form of conventional and novel PKCs (Ser660 on the hidrophobic site) (bottom). When at least 10 µM PKCzInh were used, a clear decrease in the intensity of the band corresponding to the atypical PKCs is observed while no difference is observed in the bands corresponding to the conventional and novel PKCs, showing that PKCzInh specifically inhibits atypical PKCs and has no effect on other PKCs. (B) Specificity of inhibition between atypical PKCs (PKCζ versus PKCι) by PKCzInh. To distinguish between PKCζ and PKCι, Huh7 cells were transfected with plasmids expressing either a tagged version of PKCζ or a tagged version of PKCι. The tag in either construct enables the overexpressed protein to be distinguished from the endogenous ones on SDS-PAGE, and the degree of autophosphorilation of each of the atypical PKCs to be analysed independently. The expression level of PKCζ is approximately 5-fold higher than that of PKCι, as shown by quantification of signal intensity of the two proteins following probing with an antibody that recognises both isoforms (left). Inhibition on those cells was only observed when 60 µM PKCzInh was used. Inhibition levels of PKCζ (middle) and PKCι (right) were assessed by probing with an antibody that specifically recognises the autophosphorilated form of atypical PKCs. The relative specificities of PKCzInh for the two isoforms were determined by calculating the percentage of inhibition of autophosphorilation of each of them and correcting for the difference in their expression levels. Our results show that the inhibitory effect on PKZζ is approximately 2-fold that observed for PKCι.
(0.16 MB PDF)

Figure S4 PKCζ in vivo knock-down does not cause any detectable or significant signs of Interferon or toxicity response on mouse livers. Effect of siRNA-mediated in vivo knock-down of PKCζ on expression of interferon or toxicity-related genes measured by qRT-PCR. Each individual graph shows expression data for one gene indicated on top. For each siRNA, RNA extracts from liver samples of 3 different mice were tested in duplicate. The same set of samples, taken 48 h after sporozoite i.v. infection was used for the entire data set. Average and SD were normalized to the expression level of Rpl13a as housekeeper. Interferon response markers: Ifna1,interferon alpha1; ifnb1, interferon beta1; Ifi44, interferon-induced protein 44; Ifit1 and 2, interferon-induced protein(s) with tetratricopeptide repeats 1 and 2; Irf7, interferon regulatory factor 7; Mx1, myxovirus resistance 1; Oas2, oligoadenylate synthetase 2; Stat1, signal transducer and activator of transcription 1. Toxicity response markers: Bax, Bcl2-associated X protein; Bcl2l11, BCL2-like 11; Fos, FBJ osteosarcoma oncogene; Fosl1; fos-like antigen 1; Fyb, FYN binding protein; Gadd45a, growth arrest and DNA-damage-inducible 45 alpha; Gapdh, glyceraldehyde-3-phosphate dehydrogenase; Hspa5, heat shock protein 5; Il18, interleukine 18; Jun, Jun oncogene; Mapk3, mitogen activated protein kinase 3; Myc, myelocytomatosis oncogene.
(0.07 MB PDF)

Figure S5 Effect of siRNA-mediated in vivo silencing of PKCζ on PKCζ protein levels. The PKCζ protein levels were measured by Western blot analysis of liver extracts collected 40 h after sporozoite i.v. injection. Mice were infected 36 h after RNAi treatment with siRNA #1. The plot shows the quantification of the amounts of PKCζ normalised to those of actin (used as a housekeeping control protein) in liver samples of mice treated with the siRNA targeting the PKCζ gene, relative to the normalised amounts of PKCζ in control samples.
(0.03 MB PDF)
