# Supplementary material for: Kinome-Wide RNAi Screen Implicates at Least 5 Host Hepatocyte Kinases in Plasmodium Sporozoite Infection
Source: PLoS Pathog. 2008 Nov 7;4(11):e1000201. doi: 10.1371/journal.ppat.1000201 (PMC2574010; doi:10.1371/journal.ppat.1000201)
Supplement: Table S1 — List of siRNAs used throughout the RNAi screen. siRNAs that led to an increase or a decrease in infection are marked in red or in green, respectively. CN denotes the normalised number of nuclei in each condition. Genes in italics are those for which one siRNA met the selection criteria but all three siRNAs used in Pass 1 gave a statistically significant (p<0,05) difference in terms of infection rate. For these genes, the three siRNAs used in Pass 1 were used again in Pass 2. (0.07 MB PDF) [file ppat.1000201.s001.pdf]

Prudencio *et al.* , Supplementary Table 1. List of siRNAs used throughout the RNAi screen.

siRNAs that led to an increase or a decrease in infection are marked in red or in green, respectively.
CN denotes the normalised number of nuclei in each condition.
Genes in *italics* are those for which one siRNA met the selection criteria but all three siRNAs used in Pass 1 gave a statistically significant (p<0,05) difference in terms of infection rate. For these genes, the three siRNAs used in Pass 1 were used again in Pass 2.

			Pass 1		Pass 2		Pass 3	
**Gene Name**	**LocusLink ID**	**siRNA ID**	**CN**	**z-score**	**CN**	**z-score**	**CN**	**z-score**
AAK1	22848	103351	87,7	0,11				
AAK1	22848	103435	125,3	1,61				
AAK1	22848	103515	103,6	1,97				
ABL1	25	1241	93,7	-0,36				
ABL1	25	1336	102,9	0,44				
ABL1	25	1431	102,2	0,50				
ABL2	27	1478	87,2	1,56				
ABL2	27	1662	102,8	0,14				
ABL2	27	103346	95,7	2,41	86,9	4,15		
ABL2	27	242409			79,4	-3,01		
ABL2	27	242410			106,0	-0,14		
ACK1	10188	721	72,9	0,93				
ACK1	10188	103335	113,5	-1,10				
ACK1	10188	103419	100,0	-0,64				
ACVR1	90	76	105,1	-2,21	86,5	-4,45		
ACVR1	90	77	111,6	-0,11				
ACVR1	90	78	102,1	-0,24				
ACVR1	90	242344			112,0	3,50		
ACVR1	90	242345			102,7	1,38		
ACVR1B	91	1331	112,3	0,34				
ACVR1B	91	103589	91,0	-0,05				
ACVR1B	91	111240	133,6	-0,05				
ACVR1C	130399	1521	95,8	0,99				
ACVR1C	130399	1612	86,4	-0,37				
ACVR1C	130399	1701	104,3	-0,38				
ACVR2	92	143	106,4	-0,94				
ACVR2	92	144	110,5	1,08				
ACVR2	92	103568	99,6	-0,16				
ACVR2B	93	79	103,1	0,37				
ACVR2B	93	80	99,8	-0,30				
ACVR2B	93	81	98,1	-0,25				
ACVRL1	94	11	98,1	0,30				
ACVRL1	94	12	108,8	0,89				
ACVRL1	94	13	120,5	-0,49				
ADCK1	57143	1176	83,2	0,37				
ADCK1	57143	1271	128,2	1,46				
ADCK1	57143	103609	101,5	0,57				
ADCK2	90956	1125	121,2	0,02				
ADCK2	90956	103614	93,0	0,22				
ADCK2	90956	103666	90,4	-0,17				
ADCK4	79934	1068	79,3	-0,17				
ADCK4	79934	1069	105,9	-0,29				
ADCK4	79934	1070	102,1	-0,83				
ADCK5	203054	43144	83,5	-0,25				
ADCK5	203054	103781	92,9	-0,25				
ADCK5	203054	103783	92,0	-0,55				
ADK	132	1474	76,3	-1,61				
ADK	132	1568	99,4	-0,65				
ADK	132	1658	97,6	-0,45				
ADRBK1	156	147	97,4	-0,53				
ADRBK1	156	103304	92,1	0,49				
ADRBK1	156	103391	118,0	-1,03				
ADRBK2	157	628	97,2	0,07				
ADRBK2	157	629	102,0	0,42				
ADRBK2	157	630	61,7	-2,40				
AK1	203	65	109,0	1,45				
AK1	203	66	104,8	0,58				
AK1	203	110741	112,7	-0,31				
AK2	204	41652	109,7	-0,46				
AK2	204	41815	105,2	0,75				
AK2	204	41815	107,7	1,16				
AK2	204	147152	107,0	-0,04				
AK3	205	915	89,4	-0,14				
AK3	205	916	105,9	3,52	85,1	4,91		
AK3	205	917	107,3	-0,43				
AK3	205	242490			93,7	3,47		
AK3	205	242491			88,2	-0,24		
AK3L1	50808	1002	95,2	0,14				
AK3L1	50808	1004	99,6	-1,33				
AK3L1	50808	103356	92,7	0,60				
AK5	26289	872	98,8	0,50				
AK5	26289	873	96,7	-0,33				
AK5	26289	874	97,6	1,01				

Gene Name	LocusLink ID	siRNA ID	Pass 1 CN	Pass 1 z-score	Pass 2 CN	Pass 2 z-score	Pass 3 CN	Pass 3 z-score
AK7	122481	129427	88,2	-0,52				
AK7	122481	129428	89,7	0,36				
AK7	122481	129429	95,5	0,94				
AKAP1	8165	139096	99,2	-0,47				
AKAP1	8165	139097	91,0	-0,59				
AKAP1	8165	139098	97,7	0,13				
AKAP10	11216	136307	110,8	0,11				
AKAP10	11216	136308	99,4	0,18				
AKAP10	11216	136309	111,0	0,39				
AKAP11	11215	136937	113,0	-1,17				
AKAP11	11215	136938	102,1	-0,04				
AKAP11	11215	136939	94,7	-0,52				
AKAP12	9590	137856	99,6	-0,67				
AKAP12	9590	137857	108,8	0,82				
AKAP12	9590	137858	106,9	-0,87				
AKAP2	11217	202406	104,4	0,07				
AKAP2	11217	202407	95,6	-0,35				
AKAP2	11217	202408	114,8	0,52				
AKAP28	158798	128414	82,7	2,49	83,9	6,74		
AKAP28	158798	202311	96,5	0,64				
AKAP28	158798	202312	86,0	0,66				
AKAP28	158798	242340			97,5	1,26		
AKAP28	158798	242341			127,1	-0,88		
AKAP3	10566	135788	66,6	-1,58				
AKAP3	10566	135789	116,3	1,59				
AKAP3	10566	135790	92,4	-1,72				
AKAP4	8852	137315	117,2	2,23				
AKAP4	8852	137316	103,1	-0,37				
AKAP4	8852	137317	87,8	-1,54				
AKAP5	9495	15339	104,2	0,13				
AKAP5	9495	137774	104,3	-0,35				
AKAP5	9495	137775	95,3	-0,01				
AKAP6	9472	137533	94,9	0,23				
AKAP6	9472	137534	94,6	0,25				
AKAP6	9472	137535	106,5	-0,48				
AKAP7	9465	138632	110,0	0,20				
AKAP7	9465	138633	96,4	0,30				
AKAP7	9465	138634	94,6	-1,13				
AKAP8	10270	135514	131,0	1,40				
AKAP8	10270	135515	94,6	0,07				
AKAP8	10270	135516	85,8	-0,79				
AKAP8L	26993	21328	83,1	-1,42				
AKAP8L	26993	21422	102,8	-0,01				
AKAP8L	26993	147522	96,1	-0,79				
AKAP9	10142	138647	110,9	-0,98				
AKAP9	10142	138648	113,5	-0,18				
AKAP9	10142	138649	52,7	-1,47				
AKIP	54998	25730	138,4	-0,19				
AKIP	54998	45342	103,3	0,56				
AKIP	54998	123005	95,5	-1,13				
AKT1	207	632	101,1	-0,42				
AKT1	207	42811	140,4	1,25				
AKT1	207	118270	81,3	-1,12				
AKT2	208	149	104,1	-0,58				
AKT2	208	150	76,2	-0,84				
AKT2	208	103305	96,0	-0,67				
AKT3	10000	695	48,5	-1,29				
AKT3	10000	697	100,0	0,23				
AKT3	10000	110901	107,3	-0,23				
ALDH18A1	5832	345	97,4	1,14				
ALDH18A1	5832	346	103,7	0,53				
ALDH18A1	5832	347	102,7	-0,25				
ALK	238	499	104,9	-0,64				
ALK	238	500	87,5	-0,19				
ALK	238	501	97,6	-0,11				
ALS2CR2	55437	1171	102,0	0,26				
ALS2CR2	55437	1266	96,9	-0,75				
ALS2CR2	55437	1361	96,1	-0,56				
ALS2CR7	65061	1140	85,9	0,45				
ALS2CR7	65061	1141	109,0	0,90				
ALS2CR7	65061	1142	106,9	-0,13				

Gene Name	LocusLink ID	siRNA ID	Pass 1 CN	Pass 1 z-score	Pass 2 CN	Pass 2 z-score	Pass 3 CN	Pass 3 z-score
AMHR2	269	1044	101,7	1,79				
AMHR2	269	1045	102,6	1,31				
AMHR2	269	1046	99,4	-0,05				
ANKK1	255239	103771	96,3	-0,47				
ANKK1	255239	103774	109,5	0,93				
ANKK1	255239	103777	87,2	-0,83				
APEG1	10290	42121	100,8	0,27				
APEG1	10290	42210	95,6	-1,08				
APEG1	10290	42274	101,8	-0,19				
ARAF1	369	151	83,8	-0,28				
ARAF1	369	152	110,8	0,51				
ARAF1	369	153	94,3	2,60				
ARK5	9891	964	103,9	0,40				
ARK5	9891	103350	106,5	0,11				
ARK5	9891	103843	59,9	-1,80				
ASB10	136371	1513	79,5	-2,19	103,6	-3,24		
ASB10	136371	45582	86,3	0,26				
ASB10	136371	45672	92,6	1,85				
ASB10	136371	242476			106,9	2,39		
ASB10	136371	242477			106,6	0,71		
ASK	10926	126686	104,4	-0,95				
ASK	10926	126687	100,3	-0,06				
ASK	10926	126688	103,0	-0,11				
ATM	472	111194	116,9	1,21				
ATM	472	118231	62,1	0,38				
ATM	472	118232	114,1	-0,37				
ATR	545	82	74,0	-1,51				
ATR	545	83	109,0	0,49				
ATR	545	103302	84,5	0,07				
AURKB	9212	494	48,8	0,56				
AURKB	9212	495	89,8	-0,23				
AURKB	9212	103587	101,5	0,13				
AURKC	6795	378	84,8	1,48				
AURKC	6795	379	116,3	0,66				
AURKC	6795	111219	108,1	0,52				
AXL	558	1218	97,9	-1,77				
AXL	558	1313	100,6	0,47				
AXL	558	1408	97,8	-0,34				
BCKDK	10295	729	102,7	-0,64				
BCKDK	10295	730	95,7	0,40				
BCKDK	10295	110905	139,8	1,34				
BCR	613	1237	96,7	1,26				
BCR	613	1332	82,6	0,92				
BCR	613	110844	112,2	-0,07				
BLK	640	154	98,5	1,23				
BLK	640	155	87,5	-0,59				
BLK	640	156	81,2	-1,64				
BMP2K	55589	1489	99,0	0,20				
BMP2K	55589	1583	116,8	0,99				
BMP2K	55589	111088	108,4	-2,75	115,1	-4,23		
BMP2K	55589	242488			104,1	1,99		
BMP2K	55589	242489			89,2	-1,94		
BMPR1A	657	502	94,6	0,38				
BMPR1A	657	503	113,2	1,17				
BMPR1A	657	504	97,1	-0,34				
BMPR1B	658	85	101,0	0,04				
BMPR1B	658	87	108,9	1,59				
BMPR1B	658	103565	107,8	0,20				
BMPR2	659	1438	100,5	0,56				
BMPR2	659	1623	97,6	-1,02				
BMPR2	659	110763	100,5	-1,53				
BMX	660	157	96,5	1,43				
BMX	660	158	133,5	1,16				
BMX	660	159	99,4	-0,38				
BRAF	673	506	88,3	-0,34				
BRAF	673	507	103,8	0,21				
BRAF	673	110846	68,6	-2,16				
BRD2	6046	118266	99,7	-0,42				
BRD2	6046	118267	77,3	0,20				
BRD2	6046	118268	97,5	-0,64				
BRD3	8019	869	83,7	0,26				
BRD3	8019	111249	92,0	3,29	100,6	7,98	80,7	1,71
BRD3	8019	111251	113,9	0,18				
BRD3	8019	242411			110,0	1,01		
BRD3	8019	242412			92,8	4,54	79,8	0,99

Gene Name	LocusLink ID	siRNA ID	Pass 1 CN	Pass 1 z-score	Pass 2 CN	Pass 2 z-score	Pass 3 CN	Pass 3 z-score
BRD4	23476	1512	122,6	0,73				
BRD4	23476	1605	64,4	0,03				
BRD4	23476	1694	97,8	-0,31				
BRDT	676	160	92,4	-0,46				
BRDT	676	161	111,4	0,74				
BRDT	676	162	102,7	-0,18				
BTK	695	14	109,8	-0,05				
BTK	695	15	109,9	0,45				
BTK	695	16	78,9	0,31				
BUB1	699	508	80,6	0,34				
BUB1	699	509	118,1	-1,70				
BUB1	699	510	101,6	-1,09				
BUB1B	701	88	74,1	-0,12				
BUB1B	701	89	84,7	-0,69				
BUB1B	701	90	67,0	-0,18				
C14orf20	283629	41401	93,6	-1,71				
C14orf20	283629	41491	101,8	0,08				
C14orf20	283629	41571	100,0	0,81				
C6orf199	221264	1209	102,0	-0,34				
C6orf199	221264	1304	106,9	-0,33				
C6orf199	221264	1399	97,8	0,24				
C9orf12	64768	1186	86,0	0,55				
C9orf12	64768	1186	90,7	0,52				
C9orf12	64768	1186	93,7	-3,29				
C9orf12	64768	1281	92,0	2,58	88,4	7,92	135,7	-0,05
C9orf12	64768	1376	98,5	-0,14				
C9orf12	64768	242460			108,6	5,95	124,2	0,48
C9orf12	64768	242461			65,0	-0,12		
C9orf98	158067	1525	110,4	0,26				
C9orf98	158067	1615	111,6	-0,75				
C9orf98	158067	111057	96,2	1,01				
CABC1	56997	1032	119,9	0,24				
CABC1	56997	110979	108,1	-0,05				
CALM1	801	146695	116,8	-0,39				
CALM1	801	146696	107,2	-0,15				
CALM1	801	146697	100,0	-0,84				
CALM2	805	145935	114,4	-0,21				
CALM2	805	145936	105,5	-0,05				
CALM2	805	145937	95,0	2,06				
CALM3	808	634	104,3	0,53				
CALM3	808	636	96,0	0,56				
CALM3	808	111245	93,3	-0,33				
CAMK1	8536	437	106,6	0,37				
CAMK1	8536	438	97,8	0,25				
CAMK1	8536	439	106,3	-0,18				
CAMK1D	57118	1035	104,6	0,65				
CAMK1D	57118	1036	94,9	-0,45				
CAMK1D	57118	1037	92,5	0,24				
CAMK1G	57172	103363	106,0	1,28				
CAMK1G	57172	103447	97,6	-0,60				
CAMK1G	57172	103527	98,6	-0,46				
CAMK2A	815	1484	110,0	-0,76				
CAMK2A	815	1578	104,8	0,01				
CAMK2A	815	1668	94,3	-1,22				
CAMK2B	816	9477	102,9	-0,57				
CAMK2B	816	9571	101,6	0,69				
CAMK2B	816	9662	101,7	-0,34				
CAMK2D	817	91	99,8	0,35				
CAMK2D	817	93	98,7	1,15				
CAMK2D	817	118256	110,8	0,74				
CAMK2G	818	40536	103,7	-0,02				
CAMK2G	818	40631	103,2	0,39				
CAMK2G	818	40719	103,8	0,62				
CAMK4	814	163	102,9	-0,11				
CAMK4	814	164	102,4	-0,31				
CAMK4	814	165	97,2	1,26				
CaMKIINalpha	55450	123137	117,6	0,92				
CaMKIINalpha	55450	202303	107,6	0,79				
CaMKIINalpha	55450	202322	85,0	-1,27				
CAMKK1	84254	103383	74,2	0,23				
CAMKK1	84254	103466	102,2	-0,26				
CAMKK1	84254	103544	103,4	-0,65				
CAMKK2	10645	815	87,9	0,24				
CAMKK2	10645	816	107,1	0,98				
CAMKK2	10645	110919	100,3	-0,47				

Gene Name	LocusLink ID	siRNA ID	Pass 1		Pass 2		Pass 3	
			CN	z-score	CN	z-score	CN	z-score
CARKL	23729	902	93,0	0,97				
CARKL	23729	903	100,0	0,00				
CARKL	23729	904	102,6	-0,43				
CASK	8573	446	108,3	-0,13				
CASK	8573	447	104,3	-0,19				
CASK	8573	448	88,8	-1,03				
CCRK	23552	875	106,1	-0,86				
CCRK	23552	876	103,4	0,15				
CCRK	23552	111072	107,2	0,84				
CDADC1	81602	1196	92,4	-0,59				
CDADC1	81602	1291	98,8	0,49				
CDADC1	81602	103612	103,7	-0,48				
CDC2	983	1625	87,3	0,05				
CDC2	983	42819	91,3	-0,21				
CDC2	983	103821	103,8	0,49				
CDC2L1	984	41656	118,5	2,66	120,4	9,27	121,8	0,17
CDC2L1	984	214537	100,5	-0,24	100,1	6,78	130,9	-1,13
CDC2L1	984	214538	61,9	-0,65	95,3	4,55	125,5	-1,65
CDC2L5	8621	1231	92,1	-0,27				
CDC2L5	8621	1326	90,5	-0,61				
CDC2L5	8621	1421	108,9	0,49				
CDC42BPA	8476	430	81,2	1,95	74,8	2,76		
CDC42BPA	8476	22066	62,2	2,24	75,8	4,23		
CDC42BPA	8476	103791	63,8	-2,07	80,4	-4,01		
CDC42BPA	8476	242428			73,9	3,20		
CDC42BPA	8476	242429			94,6	3,11		
CDC42BPB	9578	746	97,1	0,67				
CDC42BPB	9578	747	85,7	-0,31				
CDC42BPB	9578	103598	108,2	-0,75				
CDC42SE2	56990	42453	113,8	0,46				
CDC42SE2	56990	42532	103,1	-0,21				
CDC42SE2	56990	127235	94,4	-0,23				
CDC7	8317	410	108,7	0,42				
CDC7	8317	411	104,6	-0,53				
CDC7	8317	412	114,4	-0,26				
CDK10	8558	1420	88,4	-1,16				
CDK10	8558	111225	94,7	-0,31				
CDK10	8558	111229	102,5	0,44				
CDK11	23097	103723	81,9	-1,88	84,9	1,50		
CDK11	23097	103731	89,0	-1,50	103,6	0,64		
CDK11	23097	103739	87,5	-2,14	132,1	1,44		
CDK11	23097	214337			105,7	2,94		
CDK2	1017	1314	70,0	1,32				
CDK2	1017	1409	107,2	-0,21				
CDK2	1017	42820	100,0	-0,01				
CDK3	1018	94	113,5	1,76				
CDK3	1018	95	108,7	-0,25				
CDK3	1018	96	111,8	1,16				
CDK4	1019	44756	90,4	0,91				
CDK4	1019	44852	97,9	-1,17				
CDK4	1019	213350	97,6	-0,73				
CDK5	1020	1466	78,8	-0,23				
CDK5	1020	1560	82,7	-1,52				
CDK5	1020	1650	103,6	-0,96				
CDK5R1	8851	459	96,2	-1,03				
CDK5R1	8851	46067	101,6	0,07				
CDK5R1	8851	110814	96,7	-0,69				
CDK6	1021	98	98,1	1,40				
CDK6	1021	99	101,8	-0,12				
CDK6	1021	42822	73,2	-0,49				
CDK7	1022	166	101,6	-0,50				
CDK7	1022	167	76,0	-0,37				
CDK7	1022	168	114,2	0,18				
CDK8	1024	100	98,7	-1,78				
CDK8	1024	101	93,5	1,45				
CDK8	1024	102	112,5	-0,30				
CDK9	1025	103	89,8	0,11				
CDK9	1025	104	102,1	0,96				
CDK9	1025	103567	79,8	-0,05				
CDKL1	8814	1459	94,7	-0,36				
CDKL1	8814	1554	84,8	2,00				
CDKL1	8814	1644	98,1	0,09				
CDKL2	8999	466	96,0	-1,22				
CDKL2	8999	467	113,2	0,31				
CDKL2	8999	468	110,8	-0,34				

Gene Name	LocusLink ID	siRNA ID	Pass 1 CN	Pass 1 z-score	Pass 2 CN	Pass 2 z-score	Pass 3 CN	Pass 3 z-score
CDKL3	51265	1154	95,9	3,38	105,2	3,56		
CDKL3	51265	1249	95,3	-1,06				
CDKL3	51265	1344	90,5	0,53				
CDKL3	51265	242438			88,6	2,57		
CDKL3	51265	242439			100,8	3,84		
CDKL5	6792	374	101,4	-0,24				
CDKL5	6792	375	114,4	-0,31				
CDKL5	6792	376	109,8	0,70				
CDKN1A	1026	1436	119,3	3,73	121,2	6,75		
CDKN1A	1026	1531	89,5	-1,91				
CDKN1A	1026	1621	115,1	0,05				
CDKN1A	1026	242474			89,1	0,34		
CDKN1A	1026	242475			78,7	3,91		
CDKN1B	1027	118712	83,4	-2,71	94,5	-7,14	115,7	1,78
CDKN1B	1027	118713	108,7	-0,85				
CDKN1B	1027	118714	107,1	-1,01				
CDKN1B	1027	242378			116,0	-3,63	95,2	-1,60
CDKN1B	1027	242379			104,0	2,24		
CDKN1C	1028	2789	94,0	-0,07				
CDKN1C	1028	118537	110,0	-0,08				
CDKN1C	1028	119424	100,6	-2,16	104,9	-4,80		
CDKN1C	1028	242331			125,8	3,83		
CDKN1C	1028	242332			90,8	-0,15		
CDKN2A	1029	118858	89,4	-0,69				
CDKN2A	1029	119508	99,2	-0,28				
CDKN2A	1029	119509	117,8	-0,09				
CDKN2B	1030	118739	70,5	-1,61				
CDKN2B	1030	118740	92,7	-0,42				
CDKN2B	1030	118741	119,0	0,43				
CDKN2C	1031	118622	86,6	-1,54				
CDKN2C	1031	118623	88,8	0,11				
CDKN2C	1031	119531	110,3	-0,89				
CDKN2D	1032	118644	81,6	0,09				
CDKN2D	1032	118645	98,1	0,71				
CDKN2D	1032	118646	97,2	-0,21				
CDKN3	1033	637	109,0	-0,18				
CDKN3	1033	45016	107,5	-0,94				
CDKN3	1033	45105	85,9	-0,47				
CERK	64781	110997	130,6	2,99				
CERK	64781	111000	82,9	-0,16				
CERK	64781	111002	96,1	-0,30				
CHEK1	1111	106	108,4	0,35				
CHEK1	1111	107	104,9	0,41				
CHEK1	1111	108	103,8	0,23				
CHEK2	11200	1477	93,7	-0,27				
CHEK2	11200	118278	123,6	-0,13				
CHEK2	11200	118298	100,3	0,37				
CHKA	1119	109	62,8	-2,34	68,5	-6,51		
CHKA	1119	110	105,8	0,31				
CHKA	1119	111	122,9	0,02				
CHKA	1119	242492			109,4	-2,63		
CHKA	1119	242493			112,5	0,15		
CHKB	1120	202425	95,4	1,08				
CHKB	1120	202426	107,2	0,32				
CHKB	1120	202427	106,2	0,43				
CHUK	1147	112	104,0	0,84				
CHUK	1147	113	108,6	-0,23				
CHUK	1147	114	101,3	-1,10				
CIB2	10518	135730	118,6	1,57				
CIB2	10518	135731	97,0	-0,12				
CIB2	10518	135732	75,2	0,84				
CIB3	117286	147982	113,1	-0,16				
CIB3	117286	147983	68,5	-1,46				
CIB3	117286	147984	95,8	0,53				
CIT	11113	103721	106,5	-0,23				
CIT	11113	103729	89,5	0,60				
CIT	11113	103737	95,3	-1,32				
CKB	1152	170	105,7	-0,66				
CKB	1152	118235	100,2	1,02				
CKB	1152	118236	76,3	-0,45				
CKM	1158	172	113,9	1,34				
CKM	1158	173	45,9	-0,97				
CKM	1158	110772	102,2	1,44				
CKMT1	1159	1178	109,4	-0,53				
CKMT1	1159	1273	101,0	-1,11				
CKMT1	1159	1368	122,4	1,94				

Gene Name	LocusLink ID	siRNA ID	Pass 1		Pass 2		Pass 3	
			CN	z-score	CN	z-score	CN	z-score
CKMT2	1160	175	101,1	-0,73				
CKMT2	1160	176	92,8	-1,03				
CKMT2	1160	177	110,2	-0,06				
CLK1	1195	479	95,0	-1,15				
CLK1	1195	480	93,8	0,26				
CLK1	1195	110837	108,2	-0,44				
CLK2	1196	43833	89,1	0,32				
CLK2	1196	43929	99,2	-0,60				
CLK2	1196	44017	109,5	1,11				
CLK3	1198	1233	98,0	1,28				
CLK3	1198	1328	116,6	-0,22				
CLK3	1198	1423	107,2	0,13				
CLK4	57396	1048	95,4	-0,04	115,0	-0,15		
CLK4	57396	1049	48,1	-1,44	55,3	-6,19		
CLK4	57396	110988	94,9	-0,86	93,8	-3,36		
CLK4	57396	212808			109,4	-1,64		
CNKSR1	10256	122592	76,5	0,11				
CNKSR1	10256	122593	111,2	-0,65				
CNKSR1	10256	122594	88,6	0,92				
COASY	80347	1195	106,2	-0,68				
COASY	80347	1290	108,0	0,20				
COASY	80347	111026	96,9	-0,33				
CRIM1	51232	135025	97,6	-1,22				
CRIM1	51232	135026	102,9	-0,27				
CRIM1	51232	135027	109,9	0,50				
CRK7	51755	1153	109,1	-0,56				
CRK7	51755	1248	76,1	-2,84	79,2	-6,42		
CRK7	51755	1343	111,0	-0,29				
CRK7	51755	242436			107,5	2,43		
CRK7	51755	242437			97,2	1,78		
CSF1R	1436	641	102,5	-0,84				
CSF1R	1436	642	102,9	0,23				
CSF1R	1436	643	89,7	-0,80				
CSK	1445	511	110,8	0,92				
CSK	1445	512	107,1	1,06				
CSK	1445	513	110,9	0,95				
CSNK1A1	1452	178	102,1	-0,48				
CSNK1A1	1452	179	98,7	-0,42				
CSNK1A1	1452	180	107,2	-0,02				
CSNK1A1L	122011	1520	124,5	-0,78				
CSNK1A1L	122011	1611	102,1	0,93				
CSNK1A1L	122011	1700	91,4	-1,32				
CSNK1D	1453	1220	101,7	1,37				
CSNK1D	1453	1315	100,1	0,62				
CSNK1D	1453	1410	95,8	-0,87				
CSNK1E	1454	1441	124,3	0,79				
CSNK1E	1454	1536	106,7	-0,08				
CSNK1E	1454	1626	89,3	-0,30				
CSNK1G1	53944	1062	115,6	1,81				
CSNK1G1	53944	1064	123,5	-0,10				
CSNK1G1	53944	110992	89,8	-0,03				
CSNK1G2	1455	115	98,9	-0,63				
CSNK1G2	1455	117	91,5	1,45				
CSNK1G2	1455	118258	106,0	-0,34				
CSNK1G3	1456	103320	98,9	-1,28				
CSNK1G3	1456	103320	102,6	-1,08				
CSNK1G3	1456	103404	88,2	-1,08				
CSNK1G3	1456	103486	75,5	-1,01				
CSNK2A1	1457	1146	107,4	1,96				
CSNK2A1	1457	1242	106,1	-0,11				
CSNK2A1	1457	1337	107,1	0,90				
CSNK2A2	1459	181	101,9	0,43				
CSNK2A2	1459	183	106,0	-0,11				
CSNK2A2	1459	103306	98,9	1,80				
CSNK2B	1460	9710	67,3	-0,30				
CSNK2B	1460	9806	106,2	-0,27				
CSNK2B	1460	9896	81,1	0,06				
DAPK1	1612	598	98,2	0,89				
DAPK1	1612	599	91,9	-0,44				
DAPK1	1612	600	104,6	0,20				
DAPK2	23604	930	109,7	-0,48				
DAPK2	23604	932	107,3	0,83				
DAPK2	23604	110948	71,4	-1,32				

Gene Name	LocusLink ID	siRNA ID	Pass 1		Pass 2		Pass 3	
			CN	z-score	CN	z-score	CN	z-score
DAPK3	1613	129	110,6	-0,31				
DAPK3	1613	103790	103,7	0,47				
DAPK3	1613	103795	111,3	-0,42				
DCAMKL1	9201	576	105,9	0,25				
DCAMKL1	9201	103591	85,4	0,69				
DCAMKL1	9201	110854	102,4	-0,20				
DCK	1633	68	104,5	-0,22				
DCK	1633	69	93,5	0,14				
DCK	1633	103564	96,8	1,51				
DDR1	780	1221	113,5	1,57				
DDR1	780	1316	100,8	0,10				
DDR1	780	1411	92,6	0,70				
DDR2	4921	757	116,3	1,61				
DDR2	4921	118276	99,4	-1,02				
DDR2	4921	118277	101,4	0,41				
DGKA	1606	118	103,9	0,19				
DGKA	1606	119	97,2	-0,59				
DGKA	1606	120	68,0	-1,92				
DGKB	1607	1235	109,0	0,10				
DGKB	1607	1330	101,2	-1,53				
DGKB	1607	1425	105,9	0,52				
DGKD	8527	1228	104,4	0,84				
DGKD	8527	1323	74,9	0,21				
DGKD	8527	1418	103,4	2,33				
DGKE	8526	434	97,3	0,15				
DGKE	8526	435	83,0	0,17				
DGKE	8526	436	103,2	0,85				
DGKG	1608	121	102,6	0,38				
DGKG	1608	122	104,9	-0,89				
DGKG	1608	123	96,9	0,38				
DGKH	160851	129947	103,4	-0,41				
DGKH	160851	129948	87,1	-0,60				
DGKH	160851	129949	102,8	1,27				
DGKI	9162	571	91,9	-0,17				
DGKI	9162	572	100,4	2,41				
DGKI	9162	573	95,2	-0,22				
DGKQ	1609	124	126,0	1,56				
DGKQ	1609	125	82,8	-0,45				
DGKQ	1609	126	104,6	-1,48				
DGKZ	8525	432	85,9	-0,93				
DGKZ	8525	433	105,0	0,25				
DGKZ	8525	111093	92,4	0,08				
DGUOK	1716	1514	81,6	-0,08				
DGUOK	1716	1606	97,3	0,69				
DGUOK	1716	1695	103,7	-1,10				
DKFZP586B1621	26007	981	107,0	0,92				
DKFZP586B1621	26007	982	98,4	0,68				
DKFZP586B1621	26007	983	106,7	-0,74				
DLG1	1739	146326	111,9	-1,03				
DLG1	1739	146327	116,2	0,60				
DLG1	1739	146328	106,0	0,37				
DLG3	1741	146766	77,7	-0,36				
DLG3	1741	146767	108,7	-0,26				
DLG3	1741	146768	79,9	-0,30				
DLG4	1742	145816	118,7	0,30				
DLG4	1742	145817	97,7	0,75				
DLG4	1742	145818	79,2	0,73				
DMPK	1760	517	94,4	-0,29				
DMPK	1760	518	92,6	0,60				
DMPK	1760	519	60,5	-1,65				
DOK1	1796	145825	125,8	0,07				
DOK1	1796	145826	81,4	-1,19				
DOK1	1796	145827	80,9	-1,21				
DTYMK	1841	1152	99,7	-0,08				
DTYMK	1841	45179	106,6	0,34				
DTYMK	1841	45274	76,3	-0,51				
DYRK1A	1859	1439	93,7	-0,39				
DYRK1A	1859	1534	132,1	1,17				
DYRK1A	1859	1624	124,3	-1,61				
DYRK1B	9149	1239	95,0	-0,33				
DYRK1B	9149	1334	92,4	-1,67				
DYRK1B	9149	1429	100,5	2,04				

Gene Name	LocusLink ID	siRNA ID	Pass 1 CN	Pass 1 z-score	Pass 2 CN	Pass 2 z-score	Pass 3 CN	Pass 3 z-score
DYRK2	8445	103339	78,1	-1,88				
DYRK2	8445	103423	106,6	1,08				
DYRK2	8445	103504	116,5	0,27				
DYRK3	8444	103315	112,9	1,96				
DYRK3	8444	103400	120,5	-0,04				
DYRK4	8798	43832	77,2	-1,28				
DYRK4	8798	43928	106,6	1,11				
DYRK4	8798	44016	83,2	-1,35				
EEF2K	29904	905	120,5	0,17				
EEF2K	29904	906	120,4	0,55				
EEF2K	29904	907	112,3	-0,14				
EGFR	1956	644	102,2	1,84				
EGFR	1956	42833	107,9	-0,33				
EGFR	1956	110895	106,8	1,15				
EIF2AK3	9451	592	98,3	-1,03				
EIF2AK3	9451	594	106,9	-0,05				
EIF2AK3	9451	103593	115,1	-1,03				
EPHA1	2041	647	106,0	-0,57				
EPHA1	2041	648	101,4	0,23				
EPHA1	2041	649	100,5	0,59				
EPHA2	1969	520	94,9	2,79	104,0	3,27		
EPHA2	1969	521	114,6	-0,14				
EPHA2	1969	110852	87,5	0,43				
EPHA2	1969	242384			106,9	2,33		
EPHA2	1969	242385			85,2	2,67		
EPHA3	2042	652	102,4	-1,85				
EPHA3	2042	103330	83,5	-1,78	73,4	-5,07	85,4	-4,49
EPHA3	2042	103414	105,7	1,08	82,9	-4,45	93,4	-2,56
EPHA4	2043	523	102,4	0,80				
EPHA4	2043	524	104,6	-0,87				
EPHA4	2043	525	79,5	-0,41				
EPHA5	2044	1460	109,2	0,57				
EPHA5	2044	1555	84,5	0,83				
EPHA5	2044	1645	111,4	0,21				
EPHA7	2045	527	93,9	-1,67				
EPHA7	2045	528	107,2	0,44				
EPHA7	2045	103321	110,9	0,99				
EPHA8	2046	1041	95,3	-1,76				
EPHA8	2046	1042	97,4	0,55				
EPHA8	2046	118289	102,5	-0,13				
EPHB1	2047	1149	111,9	1,08				
EPHB1	2047	1245	106,1	0,39				
EPHB1	2047	1340	101,7	-0,94				
EPHB2	2048	1487	102,2	0,00				
EPHB2	2048	1581	97,0	0,18				
EPHB2	2048	1671	106,5	0,63				
EPHB3	2049	529	112,8	-0,13				
EPHB3	2049	530	102,3	0,66				
EPHB3	2049	531	108,7	-0,08				
EPHB4	2050	533	104,9	-0,41				
EPHB4	2050	103322	108,9	0,91				
EPHB4	2050	103406	101,7	-0,21				
EPHB6	2051	535	100,7	-0,02				
EPHB6	2051	536	97,3	-0,40				
EPHB6	2051	537	107,3	0,66				
ERBB2	2064	42836	98,2	-0,60				
ERBB2	2064	103546	105,1	0,81				
ERBB2	2064	103552	93,3	1,26				
ERBB3	2065	184	87,6	-0,82				
ERBB3	2065	185	84,8	-1,24				
ERBB3	2065	186	104,5	0,52				
ERBB4	2066	103331	112,2	1,96				
ERBB4	2066	103415	84,0	-1,32				
ERBB4	2066	103497	112,0	0,35				
ERF	2077	3387	131,3	0,41				
ERF	2077	3483	105,9	-1,06				
ERF	2077	115797	92,4	-0,07				
ERK8	225689	1205	108,6	0,16				
ERK8	225689	1300	98,0	-0,63				
ERK8	225689	1395	90,4	-0,53				
ERN1	2081	130	104,4	0,86				
ERN1	2081	131	91,7	1,29				
ERN1	2081	132	80,4	-1,14				

Gene Name	LocusLink ID	siRNA ID	Pass 1 CN	Pass 1 z-score	Pass 2 CN	Pass 2 z-score	Pass 3 CN	Pass 3 z-score
ERN2	10595	1201	88,8	-1,01				
ERN2	10595	1296	90,1	-1,81				
ERN2	10595	1391	94,6	0,59				
ETNK1	55500	1172	99,4	-0,05				
ETNK1	55500	1267	115,8	0,90				
ETNK1	55500	103362	102,4	2,72				
ETNK2	55224	1165	116,8	0,26				
ETNK2	55224	1260	97,0	-2,26				
ETNK2	55224	1355	95,5	-1,53				
FASTK	10922	44224	102,3	-1,00				
FASTK	10922	44317	112,3	0,60				
FASTK	10922	44407	115,5	0,57				
FBXW3	26226	213333	105,0	-0,22				
FBXW3	26226	213334	46,4	-0,24				
FBXW3	26226	213335	99,2	-0,02				
FER	2241	657	98,4	-0,12				
FER	2241	658	100,3	-0,67				
FER	2241	103594	100,7	-0,22				
FES	2242	187	99,0	-0,79				
FES	2242	188	85,9	-0,31				
FES	2242	189	93,1	1,30				
FGFR1	2260	1216	109,0	-0,16				
FGFR1	2260	1311	103,4	-0,56				
FGFR1	2260	118296	116,8	1,62				
FGFR2	2263	1215	108,8	-1,19				
FGFR2	2263	118291	101,2	0,03				
FGFR2	2263	118292	104,2	-0,93				
FGFR3	2261	103815	86,3	-0,45				
FGFR3	2261	110725	107,4	0,33				
FGFR3	2261	110728	105,1	-0,72				
FGFR4	2264	103307	71,8	0,07				
FGFR4	2264	103394	103,3	-0,17				
FGFR4	2264	103816	113,1	0,69				
FGR	2268	1468	89,9	1,40				
FGR	2268	1562	95,7	-0,03				
FGR	2268	1652	93,1	-0,70				
FLJ10074	55681	110966	102,6	-0,44				
FLJ10074	55681	110968	94,5	0,24				
FLJ10074	55681	110969	99,5	-0,40				
FLJ10842	55750	1166	77,2	-2,53	87,1	-4,36		
FLJ10842	55750	1261	100,7	0,05				
FLJ10842	55750	1356	101,0	0,85				
FLJ10842	55750	242444			135,5	2,10		
FLJ10842	55750	242445			73,5	0,75		
FLJ12476	64799	1188	87,6	0,17				
FLJ12476	64799	1283	107,7	-0,60				
FLJ12476	64799	1378	92,5	0,04				
FLJ13052	65220	1189	107,4	0,58				
FLJ13052	65220	1284	92,3	0,85				
FLJ13052	65220	1379	100,0	0,81				
FLJ20574	54986	1257	75,1	0,02				
FLJ20574	54986	103361	104,9	-0,48	131,0	-0,79		
FLJ20574	54986	103445	65,9	2,59	62,1	5,46		
FLJ23074	80122	1071	87,9	-0,65				
FLJ23074	80122	1072	97,1	-0,40				
FLJ23074	80122	1073	94,2	-0,26				
FLJ23356	84197	1098	96,0	1,59				
FLJ23356	84197	1099	111,4	0,87				
FLJ23356	84197	1100	104,8	0,11				
FLJ25006	124923	1207	116,8	-0,40				
FLJ25006	124923	1397	100,9	2,18				
FLJ25006	124923	103615	97,3	0,74				
FLJ32685	152110	103550	109,4	0,94				
FLJ32685	152110	103556	109,1	-1,68				
FLJ32685	152110	111053	103,3	0,92				
FLJ34389	197259	42942	94,9	1,60				
FLJ34389	197259	43032	95,6	-0,78				
FLJ34389	197259	43109	99,9	-0,06				
FLT1	2321	190	109,9	-0,38				
FLT1	2321	191	94,3	-0,65				
FLT1	2321	192	112,0	0,37				
FLT3	2322	487	70,9	-0,54				
FLT3	2322	488	105,4	1,28				
FLT3	2322	489	85,8	0,24				

Gene Name	LocusLink ID	siRNA ID	Pass 1 CN	Pass 1 z-score	Pass 2 CN	Pass 2 z-score	Pass 3 CN	Pass 3 z-score
FLT3LG	2323	44790	106,1	-0,55				
FLT3LG	2323	44886	102,1	0,16				
FLT3LG	2323	144682	111,6	-0,03				
FLT4	2324	193	75,4	-1,74				
FLT4	2324	194	100,9	0,09				
FLT4	2324	195	96,2	-1,42				
FN3K	64122	1183	100,9	-0,18				
FN3K	64122	1278	94,5	0,79				
FN3K	64122	1373	85,9	-0,76				
FN3KRP	79672	1190	111,2	-0,31				
FN3KRP	79672	1285	105,0	1,36				
FN3KRP	79672	1380	75,0	1,64				
FRAP1	2475	601	87,5	1,01				
FRAP1	2475	602	126,6	0,66				
FRAP1	2475	603	68,9	-2,84	85,9	-6,23		
FRAP1	2475	242386			87,4	-1,24		
FRAP1	2475	242387			71,4	1,22		
FRK	2444	1147	86,1	0,50				
FRK	2444	1243	99,9	-0,26				
FRK	2444	1338	99,0	-1,05				
FUK	197258	111047	111,8	0,04				
FUK	197258	111049	95,3	2,57				
FUK	197258	111051	85,0	-1,61				
FYN	2534	1442	113,6	-0,60				
FYN	2534	1537	99,7	-0,86				
FYN	2534	1627	103,5	0,42				
GAK	2580	661	104,7	-0,88				
GAK	2580	103332	94,8	-1,06				
GAK	2580	103416	108,9	0,86				
GALK1	2584	20	101,8	0,45				
GALK1	2584	21	103,2	1,58				
GALK1	2584	22	92,3	0,40				
GALK2	2585	198	102,5	-0,22				
GALK2	2585	103570	93,0	-1,05				
GALK2	2585	103624	103,5	-0,98				
GCK	2645	1507	104,8	-0,28				
GCK	2645	1600	106,8	-0,04				
GCK	2645	1690	99,1	2,50				
GCKR	2646	144707	73,8	-1,78				
GCKR	2646	144708	101,0	1,73				
GCKR	2646	144709	108,5	2,71				
GK	2710	24	81,4	-0,81				
GK	2710	25	100,4	0,61				
GK	2710	103562	111,6	-0,73				
GK2	2712	1200	96,1	1,23				
GK2	2712	1295	103,8	1,37				
GK2	2712	1390	92,9	-0,31				
GKAP1	80318	1384	95,1	1,00				
GKAP1	80318	127538	121,4	1,93				
GKAP1	80318	127539	108,2	0,31				
GNE	10020	698	77,4	-0,38				
GNE	10020	699	88,4	-1,92				
GNE	10020	700	88,8	-0,84				
GRK1	6011	349	87,1	-2,36	97,4	-5,29		
GRK1	6011	111213	94,4	1,63				
GRK1	6011	111215	94,6	2,40	102,2	6,47		
GRK1	6011	242366			106,8	3,60		
GRK1	6011	242367			108,9	3,75		
GRK4	2868	662	86,2	-0,50				
GRK4	2868	663	84,3	0,24				
GRK4	2868	664	101,1	0,67				
GRK5	2869	666	106,9	1,24				
GRK5	2869	667	99,4	-0,89				
GRK5	2869	110898	126,4	-0,68				
GRK6	2870	199	104,8	0,99				
GRK6	2870	200	108,8	-0,24				
GRK6	2870	201	97,5	0,49				
GRK7	131890	1206	96,5	0,58	89,0	6,37		
GRK7	131890	1301	100,0	2,08	99,9	4,68		
GRK7	131890	1396	84,0	0,37	93,5	0,56		
GSG2	83903	1093	105,8	0,13				
GSG2	83903	1094	81,7	-0,31				
GSG2	83903	103371	98,2	2,55	95,6	0,48		
GSG2	83903	242466			118,1	-0,63		
GSG2	83903	242467			120,3	0,63		

Gene Name	LocusLink ID	siRNA ID	Pass 1		Pass 2		Pass 3	
			CN	z-score	CN	z-score	CN	z-score
GSK3A	2931	1492	83,1	0,43				
GSK3A	2931	1586	116,6	2,10				
GSK3A	2931	1676	104,2	-0,63				
GSK3B	2932	202	101,9	0,36				
GSK3B	2932	203	89,2	-1,30				
GSK3B	2932	204	92,4	0,14				
GUCY2C	2984	604	103,8	-1,23				
GUCY2C	2984	605	110,7	0,10				
GUCY2C	2984	606	115,9	0,50				
GUCY2D	3000	26	85,1	-0,23				
GUCY2D	3000	27	85,4	-0,37				
GUCY2D	3000	28	111,5	1,35				
GUCY2F	2986	133	122,1	-1,84				
GUCY2F	2986	134	98,3	-0,49				
GUCY2F	2986	135	112,9	-0,83				
GUK1	2987	71	99,0	3,04	104,3	6,26	135,9	2,72
GUK1	2987	72	106,4	2,62	112,1	6,91	129,8	4,29
GUK1	2987	103786	103,2	1,37				
GUK1	2987	242335			60,6	-0,86		
GUK1	2987	242336			99,6	0,77		
HAK	115701	1128	103,9	-1,23				
HAK	115701	1130	78,1	-2,47	98,9	-6,92		
HAK	115701	111041	87,0	-0,61				
HAK	115701	242470			98,1	-2,94		
HAK	115701	242471			103,5	4,62		
HCK	3055	205	82,0	-1,43	86,5	-7,23	88,8	-4,69
HCK	3055	206	93,1	0,61	105,6	0,19		
HCK	3055	207	83,0	-2,16	107,7	-6,73	83,6	-6,65
HGS	9146	137616	68,4	-0,72				
HGS	9146	137617	83,9	-0,21				
HGS	9146	137618	54,4	-1,15				
HIPK1	204851	39638	106,1	-1,29				
HIPK1	204851	111081	90,4	0,41				
HIPK1	204851	111083	101,7	0,08				
HIPK2	28996	1375	96,2	-0,66				
HIPK2	28996	116346	74,5	-0,69				
HIPK2	28996	116347	90,3	-0,55				
HIPK3	10114	713	82,6	-1,14				
HIPK3	10114	714	108,1	-0,18				
HIPK3	10114	715	110,6	-0,27				
HIPK4	147746	1143	119,0	1,59				
HIPK4	147746	1144	115,0	0,65				
HIPK4	147746	1145	98,3	-0,69				
HK1	3098	1506	98,4	0,34				
HK1	3098	1599	82,7	0,39				
HK1	3098	1689	84,4	-0,85				
HK2	3099	1433	88,9	0,55				
HK2	3099	1528	55,3	-0,88				
HK2	3099	1618	105,6	-0,12				
HK3	3101	208	95,6	0,70				
HK3	3101	209	90,1	-1,03				
HK3	3101	210	96,7	-0,85				
HRI	27102	942	106,9	0,30				
HRI	27102	943	97,7	0,26				
HRI	27102	944	108,4	1,29				
HSPB8	26353	933	77,2	-1,03				
HSPB8	26353	934	79,9	-0,34				
HSPB8	26353	935	80,7	0,67				
HUNK	30811	951	95,8	-0,55				
HUNK	30811	952	97,3	0,03				
HUNK	30811	953	90,7	-0,49				
ICK	22858	972	106,6	-0,05				
ICK	22858	974	107,5	0,42				
ICK	22858	103352	104,2	-0,55				
IGF1R	3480	74	103,5	0,25				
IGF1R	3480	103301	98,5	-0,16				
IGF1R	3480	110754	100,2	1,79				
IHPK1	9807	1213	82,0	-0,64				
IHPK1	9807	1308	105,6	-1,55				
IHPK1	9807	1403	120,8	1,07				
IHPK2	51447	134946	108,5	1,47				
IHPK2	51447	134947	86,8	-1,35				
IHPK2	51447	134948	89,2	0,12				

Gene Name	LocusLink ID	siRNA ID	Pass 1 CN	Pass 1 z-score	Pass 2 CN	Pass 2 z-score	Pass 3 CN	Pass 3 z-score
IHPK3	117283	1203	104,2	0,58				
IHPK3	117283	1298	105,9	-0,41				
IHPK3	117283	1393	101,4	0,68				
IKBKAP	8518	139263	93,0	0,47				
IKBKAP	8518	139264	65,1	-1,54				
IKBKAP	8518	139265	109,2	-0,25				
IKBKB	3551	147120	100,1	-0,32				
IKBKB	3551	147121	136,8	1,33				
IKBKB	3551	147122	102,7	0,70				
IKBKE	9641	918	97,2	-3,14	99,6	-0,50		
IKBKE	9641	919	56,8	-1,11				
IKBKE	9641	920	110,1	0,44				
IKBKE	9641	242417			128,5	3,02		
IKBKE	9641	242418			98,2	0,72		
IKBKG	8517	139260	97,0	0,56				
IKBKG	8517	139261	108,6	1,06				
IKBKG	8517	139262	98,6	-0,24				
ILK	3611	1461	105,0	0,02				
ILK	3611	1556	104,8	0,79				
ILK	3611	1646	99,7	-0,29				
INSR	3643	103297	106,2	-0,22				
INSR	3643	103384	95,3	-0,57				
INSR	3643	103467	100,1	-1,02				
INSRR	3645	103722	109,4	0,27				
INSRR	3645	103730	91,8	0,00				
INSRR	3645	103738	105,3	0,05				
IPMK	253430	38438	98,2	-0,32				
IPMK	253430	38524	106,2	-0,59				
IPMK	253430	148096	100,8	-0,09				
IRAK1	3654	136	102,3	0,12				
IRAK1	3654	137	100,1	0,21				
IRAK1	3654	138	98,8	-1,23				
IRAK2	3656	139	93,4	-0,03				
IRAK2	3656	140	105,3	1,70				
IRAK2	3656	141	98,9	1,68				
IRAK3	11213	857	109,4	0,20				
IRAK3	11213	858	113,2	-0,54				
IRAK3	11213	859	104,7	0,23				
IRAK4	51135	993	89,0	-0,60				
IRAK4	51135	994	102,4	0,19				
IRAK4	51135	995	107,3	-0,60				
ITK	3702	701	90,2	-0,62				
ITK	3702	702	104,4	-0,15				
ITK	3702	703	107,5	0,13				
ITPK1	3705	921	101,7	0,71				
ITPK1	3705	922	99,4	1,99				
ITPK1	3705	923	100,7	1,13				
ITPKA	3706	211	94,2	0,24				
ITPKA	3706	212	70,2	-0,11				
ITPKA	3706	213	97,5	-0,41				
ITPKB	3707	214	97,9	0,57				
ITPKB	3707	215	86,5	-1,16				
ITPKB	3707	216	109,0	0,20				
ITPKC	80271	1193	99,9	-0,24				
ITPKC	80271	1288	93,0	-0,61				
ITPKC	80271	1383	112,6	0,51				
JAK1	3716	217	85,6	0,15				
JAK1	3716	218	113,6	-1,95				
JAK1	3716	219	107,6	0,06				
JAK2	3717	607	90,6	-0,70				
JAK2	3717	608	66,4	-1,59				
JAK2	3717	609	109,8	0,64				
JAK3	3718	32	99,8	-0,71				
JAK3	3718	33	104,3	-0,19				
JAK3	3718	34	100,0	1,58				
JIK	51347	999	97,7	-0,20				
JIK	51347	1000	101,0	-0,43				
JIK	51347	1001	102,5	-0,04				
KDR	3791	220	97,0	-1,62				
KDR	3791	221	94,4	-0,05				
KDR	3791	222	111,7	-0,08				
KHK	3795	103298	111,3	0,37				
KHK	3795	103385	96,4	1,45				
KHK	3795	103468	107,0	1,08				

Gene Name	LocusLink ID	siRNA ID	Pass 1 CN	Pass 1 z-score	Pass 2 CN	Pass 2 z-score	Pass 3 CN	Pass 3 z-score
KIAA0551	23043	202509	98,8	0,04				
KIAA0551	23043	202510	110,6	-0,68				
KIAA0551	23043	202511	89,9	-1,26				
KIAA0626	9848	1180	93,9	-0,64				
KIAA0626	9848	1275	88,6	-1,58				
KIAA0626	9848	1370	84,0	-0,84				
KIAA0999	23387	1591	104,4	0,55				
KIAA0999	23387	1681	93,2	-0,07				
KIAA0999	23387	103611	108,7	1,00				
KIAA1446	57596	140672	88,1	-0,03				
KIAA1446	57596	140673	97,4	-0,92				
KIAA1446	57596	140674	80,8	-0,77				
KIAA1765	85443	202523	110,4	2,24				
KIAA1765	85443	202524	128,8	0,79				
KIAA1765	85443	202525	85,4	-0,41				
KIAA1804	84451	1503	95,0	-1,96				
KIAA1804	84451	103373	97,4	-0,61				
KIAA1804	84451	103457	105,9	0,33				
KIAA1811	84446	1502	110,1	-0,92				
KIAA1811	84446	1595	101,4	0,59				
KIAA1811	84446	1685	103,0	-0,48				
KIS	127933	1208	83,8	-0,16				
KIS	127933	1303	94,9	0,47				
KIS	127933	1398	93,9	-2,58	96,4	4,44		
KIS	127933	242484			92,7	-0,10		
KIS	127933	242485			112,0	3,96		
KIT	3815	35	94,7	0,95				
KIT	3815	36	101,0	-0,49				
KIT	3815	37	102,6	-0,03				
Ksr	16706	71029	101,9	-1,46				
Ksr	16706	71124	99,2	-0,98				
Ksr	16706	71216	106,4	0,14				
KSR2	283455	41069	90,0	-1,06				
KSR2	283455	41165	89,4	-0,64				
KSR2	283455	111068	103,0	0,17				
LAK	80216	1075	123,1	1,32				
LAK	80216	1076	78,3	-1,21				
LAK	80216	103610	81,6	-1,81				
LATS1	9113	565	105,4	0,18				
LATS1	9113	566	104,7	0,08				
LATS1	9113	567	100,5	-0,18				
LATS2	26524	948	96,3	0,18				
LATS2	26524	949	94,5	0,26				
LCK	3932	668	110,4	-1,02				
LCK	3932	669	111,9	1,28				
LCK	3932	670	114,1	1,14				
LIM	10611	126673	92,4	0,20				
LIM	10611	126674	105,5	-2,57	102,7	-7,56		
LIM	10611	126675	83,5	-0,75				
LIM	10611	242404			93,7	4,93		
LIM	10611	242405			98,1	2,77		
LIMK1	3984	1223	92,9	-0,75				
LIMK1	3984	1318	102,2	0,85				
LIMK1	3984	1413	107,4	-0,52				
LIMK2	3985	1469	120,8	0,49				
LIMK2	3985	1563	101,6	0,37				
LIMK2	3985	1653	105,0	-0,99				
LMTK2	22853	969	106,1	1,07				
LMTK2	22853	970	109,8	0,02				
LMTK2	22853	971	109,3	-0,12				
LOC149420	149420	1526	105,0	0,04				
LOC149420	149420	1616	96,4	-0,48				
LOC149420	149420	1706	103,9	-2,67	98,5	2,90		
LOC149420	149420	242480			102,9	1,37		
LOC149420	149420	242481			106,7	-2,62		
LOC283846	283846	202497	94,1	-0,37				
LOC283846	283846	202498	107,7	-0,87				
LOC283846	283846	202499	109,8	-0,23				
LOC375133	375133	202463	90,1	0,35				
LOC375133	375133	202630	105,9	-0,43				
LOC375133	375133	202631	110,7	-0,61				
LOC375449	375449	202348	110,9	-1,49				
LOC375449	375449	202364	100,3	0,49				
LOC375449	375449	202379	104,5	0,23				

Gene Name	LocusLink ID	siRNA ID	Pass 1 CN	Pass 1 z-score	Pass 2 CN	Pass 2 z-score	Pass 3 CN	Pass 3 z-score
LOC91807	91807	103772	93,8	2,65	97,2	-1,91		
LOC91807	91807	103775	99,2	0,55				
LOC91807	91807	103778	97,5	0,28	108,0	1,64		
LRRK1	79705	1497	96,6	1,09				
LRRK1	79705	1590	91,4	0,53				
LRRK1	79705	1680	107,5	0,46				
LTK	4058	223	92,5	0,70				
LTK	4058	224	96,5	-0,77				
LTK	4058	111201	111,0	-0,20				
LY6G5B	58496	42389	98,1	-0,18				
LY6G5B	58496	42474	106,9	-1,34				
LY6G5B	58496	42549	94,8	0,44				
LYK5	92335	42950	59,4	0,38				
LYK5	92335	43040	105,0	0,63				
LYK5	92335	43116	113,5	0,95				
LYN	4067	226	114,5	-0,56				
LYN	4067	227	86,9	-0,58				
LYN	4067	103571	89,2	0,57				
MADD	8567	139308	96,0	-0,13				
MADD	8567	139309	105,6	1,38				
MADD	8567	139310	122,0	1,82				
MAGI1	154043	129762	95,9	0,02				
MAGI1	154043	129763	108,4	-0,06				
MAGI1	154043	129764	106,5	0,54				
MAGI-3	260425	123257	93,7	-0,73				
MAGI-3	260425	123258	114,8	-0,20				
MAGI-3	260425	123259	103,6	-1,07				
MAK	4117	734	103,2	-0,42				
MAK	4117	735	109,1	0,72				
MAK	4117	736	88,3	-0,51				
MAP2K1	5604	324	66,7	-1,56				
MAP2K1	5604	325	119,6	-0,61				
MAP2K1	5604	326	97,3	2,59				
MAP2K1IP1	8649	138573	67,6	-2,26	86,7	-1,55		
MAP2K1IP1	8649	138574	96,8	-1,46				
MAP2K1IP1	8649	138575	95,7	-0,92				
MAP2K1IP1	8649	242456			103,0	3,74		
MAP2K1IP1	8649	242457			109,1	2,12		
MAP2K2	5605	1080	79,6	-0,15				
MAP2K2	5605	1081	96,7	1,69				
MAP2K2	5605	1082	102,8	1,41				
MAP2K3	5606	1518	107,5	1,24				
MAP2K3	5606	1609	105,3	0,77				
MAP2K3	5606	1698	117,9	-0,09				
MAP2K4	6416	363	101,6	0,16				
MAP2K4	6416	103580	92,6	-0,36				
MAP2K4	6416	103634	103,7	-1,47				
MAP2K5	5607	1519	89,4	-0,94				
MAP2K5	5607	1699	101,3	1,78				
MAP2K5	5607	118252	95,7	0,38				
MAP2K6	5608	1226	76,4	-0,26				
MAP2K6	5608	1321	94,8	1,06				
MAP2K6	5608	103576	79,4	-1,74				
MAP2K7	5609	103744	104,3	-0,62				
MAP2K7	5609	103745	98,7	1,14				
MAP2K7	5609	103746	116,5	0,11				
MAP3K1	4214	202512	101,9	-0,07				
MAP3K1	4214	202513	110,7	2,62				
MAP3K1	4214	202514	103,4	0,31				
MAP3K10	4294	1444	110,8	-1,19				
MAP3K10	4294	1539	97,4	-0,61				
MAP3K10	4294	1629	100,4	-0,09				
MAP3K11	4296	235	112,4	0,95				
MAP3K11	4296	236	108,0	1,71				
MAP3K11	4296	103787	87,0	0,45				
MAP3K12	7786	103338	103,9	0,57				
MAP3K12	7786	103422	106,6	-0,16				
MAP3K12	7786	103503	103,8	0,25				
MAP3K13	9175	103327	92,4	-0,63				
MAP3K13	9175	103411	102,1	-0,30				
MAP3K13	9175	103493	106,5	0,06				
MAP3K14	9020	474	110,1	0,20				
MAP3K14	9020	118262	93,6	0,82				
MAP3K14	9020	118263	98,1	0,50				

Gene Name	LocusLink ID	siRNA ID	Pass 1 CN	Pass 1 z-score	Pass 2 CN	Pass 2 z-score	Pass 3 CN	Pass 3 z-score
MAP3K2	10746	824	104,9	-0,90				
MAP3K2	10746	825	104,6	1,42				
MAP3K2	10746	103340	97,4	-0,39				
MAP3K3	4215	234	120,9	0,47				
MAP3K3	4215	103572	94,5	-0,45				
MAP3K3	4215	103626	99,0	-0,84				
MAP3K4	4216	1470	98,3	1,40				
MAP3K4	4216	1564	99,1	-0,21				
MAP3K4	4216	1654	103,7	0,79				
MAP3K5	4217	737	102,8	-0,64				
MAP3K5	4217	738	114,6	1,82				
MAP3K5	4217	739	105,6	1,77				
MAP3K6	9064	1558	109,9	0,00				
MAP3K6	9064	103325	98,8	0,78				
MAP3K6	9064	103491	99,1	1,57				
MAP3K7	6885	1522	90,1	-0,94				
MAP3K7	6885	1613	96,7	-0,39				
MAP3K7	6885	1702	102,6	0,41				
MAP3K7IP1	10454	17476	103,1	-0,66				
MAP3K7IP1	10454	17569	102,1	-1,01				
MAP3K7IP1	10454	135631	108,7	-0,58				
MAP3K7IP2	23118	136739	104,8	0,22				
MAP3K7IP2	23118	136740	79,0	-2,00				
MAP3K7IP2	23118	136741	100,8	0,39				
MAP3K8	1326	118272	110,4	-1,27				
MAP3K8	1326	118273	95,4	-0,92				
MAP3K8	1326	118274	101,1	-0,24				
MAP3K9	4293	202330	124,1	1,18				
MAP3K9	4293	202394	85,3	-2,26				
MAP3K9	4293	202395	67,8	-0,05				
MAP4K1	11184	854	126,3	0,11				
MAP4K1	11184	855	97,8	-0,11				
MAP4K1	11184	856	126,5	0,52				
MAP4K2	5871	550	100,4	0,15				
MAP4K2	5871	103590	98,3	-0,59				
MAP4K2	5871	103643	108,5	0,17				
MAP4K3	8491	1455	94,8	-0,73				
MAP4K3	8491	1550	99,3	1,82				
MAP4K3	8491	1640	103,2	-0,30				
MAP4K4	9448	1240	131,0	-0,87				
MAP4K4	9448	1335	116,2	0,08				
MAP4K4	9448	1430	90,3	-0,41				
MAP4K5	11183	822	90,5	-0,21				
MAP4K5	11183	103603	101,6	0,28				
MAP4K5	11183	103656	100,5	-1,63				
MAPK1	5594	1449	70,6	0,89				
MAPK1	5594	1544	108,8	-0,22				
MAPK1	5594	1634	110,2	-0,14				
MAPK10	5602	1517	85,2	0,14				
MAPK10	5602	1608	96,2	0,44				
MAPK10	5602	103378	101,3	2,47				
MAPK11	5600	103311	113,7	0,85				
MAPK11	5600	103807	94,7	0,06				
MAPK11	5600	103810	114,2	1,49				
MAPK12	6300	360	102,8	0,27				
MAPK12	6300	44843	120,4	1,92				
MAPK12	6300	44935	90,5	-1,69				
MAPK13	5603	321	99,5	0,34				
MAPK13	5603	322	86,5	1,15				
MAPK13	5603	323	99,4	1,52				
MAPK14	1432	1217	89,5	-0,23				
MAPK14	1432	1312	110,8	-1,04				
MAPK14	1432	111198	102,0	-0,60				
MAPK3	5595	142304	100,3	-0,48				
MAPK3	5595	142305	81,0	1,98				
MAPK3	5595	202320	96,7	-0,75				
MAPK4	5596	318	102,7	-0,31				
MAPK4	5596	319	91,9	-1,05				
MAPK4	5596	320	60,8	-1,67				
MAPK6	5597	1450	118,1	1,52				
MAPK6	5597	1545	88,2	-3,22	71,4	-6,15		
MAPK6	5597	1635	111,2	0,78				
MAPK6	5597	242364			88,1	-0,15		
MAPK6	5597	242365			78,2	-1,23		

Gene Name	LocusLink ID	siRNA ID	Pass 1 CN	Pass 1 z-score	Pass 2 CN	Pass 2 z-score	Pass 3 CN	Pass 3 z-score
MAPK7	5598	1414	107,0	-0,01				
MAPK7	5598	110787	105,3	-1,01				
MAPK7	5598	110792	102,8	0,48				
MAPK8	5599	1225	97,5	0,02				
MAPK8	5599	1320	83,5	0,04				
MAPK8	5599	1415	107,0	-0,62				
MAPK8IP1	9479	16356	133,9	-0,05				
MAPK8IP1	9479	16447	107,6	0,06				
MAPK8IP1	9479	137929	98,1	-0,42				
MAPK8IP2	23542	19889	100,9	-0,31				
MAPK8IP2	23542	19983	111,4	1,19				
MAPK8IP2	23542	20076	107,5	-0,26				
MAPK8IP3	23162	136770	133,1	0,95				
MAPK8IP3	23162	136771	99,5	2,15				
MAPK8IP3	23162	136772	112,5	-0,34				
MAPK9	5601	1452	109,9	0,38				
MAPK9	5601	1547	86,8	1,20				
MAPK9	5601	1637	115,3	-1,51				
MAPKAP1	79109	130548	77,9	-0,45				
MAPKAP1	79109	130549	100,9	0,41				
MAPKAP1	79109	130550	79,6	0,43				
MAPKAPK2	9261	1598	97,5	-0,38				
MAPKAPK2	9261	1688	98,2	0,80				
MAPKAPK2	9261	103613	88,6	0,14				
MAPKAPK3	7867	562	87,8	-1,58				
MAPKAPK3	7867	563	89,6	0,07				
MAPKAPK3	7867	564	102,3	-0,45				
MAPKAPK5	8550	1229	88,6	-0,70				
MAPKAPK5	8550	1419	100,9	-1,05				
MAPKAPK5	8550	103379	105,3	-0,75				
MARCKS	4082	11454	122,8	0,66				
MARCKS	4082	143479	102,0	0,54				
MARCKS	4082	143480	103,0	-0,47				
MARK1	4139	1173	97,8	-0,97				
MARK1	4139	1268	97,1	0,27				
MARK1	4139	1363	106,1	-0,68				
MARK2	2011	1582	90,4	-1,11				
MARK2	2011	103359	103,0	-2,08	127,2	-5,39	80,1	-6,07
MARK2	2011	103443	94,8	-0,41	109,3	-4,04	120,0	1,78
MARK2	2011	242440			95,7	-1,80		
MARK2	2011	242441			97,3	1,13		
MARK3	4140	229	110,4	0,87				
MARK3	4140	230	92,9	-0,19				
MARK3	4140	231	97,9	-0,38				
MARK4	57787	1089	98,1	-0,11				
MARK4	57787	1090	108,1	2,21				
MARK4	57787	111028	80,8	-0,06				
MAST2	23139	975	82,2	-1,19				
MAST2	23139	977	84,8	-0,65				
MAST2	23139	103353	85,7	1,22				
MAST3	23031	202506	140,5	0,37				
MAST3	23031	202507	103,8	-0,01				
MAST3	23031	202508	116,6	-0,14				
MASTL	84930	1108	104,8	-0,17				
MASTL	84930	1109	102,8	-0,50				
MASTL	84930	111039	88,6	-1,16				
MATK	4145	1443	103,4	-0,10				
MATK	4145	1538	94,3	0,44				
MATK	4145	1628	106,2	1,18				
MELK	9833	960	93,9	-0,22				
MELK	9833	961	90,8	-0,50				
MELK	9833	103349	98,4	0,21				
MERTK	10461	810	108,1	0,93				
MERTK	10461	811	108,6	-1,38				
MET	4233	42825	100,0	-0,84				
MET	4233	242542	85,0	-2,12	80,7	-4,22	61,2	-4,16
MET	4233	242543	66,2	-2,31	77,9	-4,89	77,5	-3,99
MET	4233	242544			97,8	0,33		
MFHAS1	9258	14331	94,7	-0,81				
MFHAS1	9258	14426	87,5	-1,23				
MFHAS1	9258	14519	101,6	0,53				
MGC16169	93627	1110	101,1	-0,24				
MGC16169	93627	1111	86,3	1,05				
MGC16169	93627	103374	98,1	-0,88				

			Pass 1		Pass 2		Pass 3	
**Gene Name**	**LocusLink ID**	**siRNA ID**	**CN**	**z-score**	**CN**	**z-score**	**CN**	**z-score**
MGC26597	206426	103381	99,5	0,70				
MGC26597	206426	103808	111,4	1,15				
MGC26597	206426	103811	101,5	0,33				
MGC42105	167359	42951	99,2	1,25				
MGC42105	167359	43041	100,9	-0,36				
MGC42105	167359	43117	87,8	-1,37				
MGC45428	166614	39203	94,7	0,48				
MGC45428	166614	39298	75,9	0,09				
MGC45428	166614	39391	95,3	-0,20				
MGC4796	83931	103725	92,7	0,40				
MGC4796	83931	103733	93,8	0,26				
MGC4796	83931	103741	100,5	0,05				
MGC8407	79012	1065	119,0	0,43				
MGC8407	79012	1066	66,8	-0,69				
MGC8407	79012	1067	65,9	-1,21				
MIDORI	57538	1495	92,3	-0,36				
MIDORI	57538	1588	76,5	-0,78				
MIDORI	57538	1678	90,6	-0,41				
MINK	50488	985	126,3	0,89				
MINK	50488	986	97,6	0,28				
MINK	50488	103382	116,6	0,17				
MKNK1	8569	445	99,2	-0,05				
MKNK1	8569	111234	100,9	-1,47				
MKNK1	8569	111236	68,0	0,82				
MKNK2	2872	1157	106,4	1,56				
MKNK2	2872	1252	104,5	0,01				
MKNK2	2872	1347	109,9	-0,35				
MLL2	8085	107160	79,2	-1,24				
MLL2	8085	107161	105,0	-0,18				
MLL2	8085	115436	109,8	0,32				
MOS	4342	671	101,9	0,49				
MOS	4342	672	106,5	-2,05	121,9	0,15		
MOS	4342	673	104,6	-0,37				
MOS	4342	242388			100,2	-0,40		
MOS	4342	242389			108,5	1,86		
MRC2	9902	749	98,5	0,94				
MRC2	9902	750	102,3	-0,50				
MRC2	9902	751	94,2	-0,72				
MST1R	4486	237	112,3	0,17				
MST1R	4486	103308	102,7	0,23				
MST1R	4486	103395	91,3	0,34				
MST4	51765	1155	88,3	-0,91				
MST4	51765	1250	99,1	-0,09				
MST4	51765	1345	79,0	-1,84				
MUSK	4593	704	82,7	-0,87				
MUSK	4593	705	104,0	0,60				
MUSK	4593	706	106,2	-0,79				
MVK	4598	56	85,2	0,61				
MVK	4598	57	101,7	-1,08				
MVK	4598	58	89,0	-0,71				
MYLK	4638	1603	97,6	-0,37				
MYLK	4638	1692	96,7	0,64				
MYLK	4638	103376	108,8	-0,75				
MYLK2	85366	103375	97,7	-0,04				
MYLK2	85366	103459	94,2	2,42	107,2	-0,84		
MYLK2	85366	103539	105,6	-0,17				
MYLK2	85366	242468			111,7	0,27		
MYLK2	85366	242469			94,3	0,24		
MYO3A	53904	1486	104,9	0,03				
MYO3A	53904	1580	95,5	-0,28				
MYO3A	53904	1670	103,4	0,28				
MYO3B	140469	1137	105,2	0,63				
MYO3B	140469	1138	112,1	-0,04				
MYO3B	140469	1139	75,1	-1,59				
NAGK	55577	1156	97,8	-0,91				
NAGK	55577	1251	95,6	1,34				
NAGK	55577	1346	103,0	1,00				
NEK1	4750	147471	105,0	-1,14				
NEK1	4750	147472	127,4	0,45				
NEK1	4750	147473	114,2	0,15				
NEK11	79858	1192	96,6	-0,32				
NEK11	79858	1287	106,7	0,54				
NEK11	79858	1382	113,7	0,26				

			Pass 1		Pass 2		Pass 3	
Gene Name	LocusLink ID	siRNA ID	CN	z-score	CN	z-score	CN	z-score
NEK2	4751	240	87,3	-2,44	90,8	-5,10		
NEK2	4751	241	54,4	-1,65				
NEK2	4751	242	112,8	0,04				
NEK2	4751	242354			106,8	1,83		
NEK2	4751	242355			107,8	2,69		
NEK3	4752	1445	96,0	-0,47				
NEK3	4752	1540	109,0	-2,33	117,4	-3,16		
NEK3	4752	1630	110,8	-1,24				
NEK3	4752	1630	108,6	-1,85				
NEK3	4752	242356			107,3	4,44		
NEK3	4752	242357			95,5	-0,70		
NEK4	6787	371	128,0	-1,44				
NEK4	6787	373	75,0	-0,33				
NEK4	6787	103581	118,1	1,73				
NEK6	10783	939	109,9	2,68				
NEK6	10783	940	102,7	-0,26				
NEK6	10783	941	106,0	0,20				
NEK7	140609	45587	98,1	-0,03				
NEK7	140609	45677	112,5	-0,14				
NEK7	140609	103794	107,1	1,01				
NEK8	284086	103726	113,6	-1,26				
NEK8	284086	103734	76,7	-1,12				
NEK8	284086	103742	93,4	1,32				
NEK9	91754	1113	118,5	0,73				
NEK9	91754	1114	98,3	-0,18				
NEK9	91754	1115	92,7	0,46				
NIPA	51530	126921	106,0	-0,58				
NIPA	51530	126922	104,0	0,78				
NIPA	51530	126923	95,2	-1,25				
NJMU-R1	64149	140704	115,3	-1,05				
NJMU-R1	64149	140705	80,6	0,21				
NJMU-R1	64149	140706	85,0	3,99	85,6	10,22	57,5	0,31
NJMU-R1	64149	242458			101,5	4,23	95,4	-1,26
NJMU-R1	64149	242459			106,2	4,80		
NLK	51701	996	84,8	-1,15				
NLK	51701	997	112,7	0,36				
NLK	51701	998	89,4	-1,19				
NME1	4830	41	97,1	1,06				
NME1	4830	42	93,2	0,03				
NME1	4830	43	85,2	-0,89				
NME2	4831	244	93,0	-0,34				
NME2	4831	245	106,2	0,36				
NME2	4831	111203	104,3	0,27				
NME3	4832	42872	89,6	-1,44				
NME3	4832	42968	89,4	0,97				
NME3	4832	118241	82,6	-0,18				
NME4	4833	610	126,2	-0,70				
NME4	4833	612	97,0	0,51				
NME4	4833	110871	102,4	0,66				
NME5	8382	13281	111,9	-0,78				
NME5	8382	13442	97,2	-0,74				
NME5	8382	139132	104,2	0,78				
NME6	10201	722	100,6	-0,46				
NME6	10201	723	111,1	0,02				
NME6	10201	724	106,7	0,71				
NME7	29922	908	104,9	1,58				
NME7	29922	45196	118,9	0,89				
NME7	29922	45290	108,0	1,08				
NPR1	4881	1437	116,0	0,37				
NPR1	4881	1532	108,3	0,11				
NPR1	4881	1622	113,8	0,41				
NPR2	4882	1234	109,5	-0,13				
NPR2	4882	1424	95,1	1,12				
NPR2	4882	110833	87,6	3,31	84,9	-1,48		
NPR2	4882	242337			109,5	3,07		
NPR2	4882	242338			111,7	5,60		
NRBP	29959	913	93,3	0,49				
NRBP	29959	914	80,6	-1,03				
NRBP	29959	103605	101,0	-1,07				
NRGN	4900	17411	103,6	-0,31				
NRGN	4900	17506	93,1	-0,89				
NRGN	4900	144200	107,5	0,39				
NTRK1	4914	42873	103,2	0,35				
NTRK1	4914	42969	103,4	0,59				
NTRK1	4914	43056	101,8	-0,25				
NTRK2	4915	752	97,8	-0,38				
NTRK2	4915	753	110,0	0,15				
NTRK2	4915	754	108,6	-0,55				

Gene Name	LocusLink ID	siRNA ID	Pass 1		Pass 2		Pass 3	
			CN	z-score	CN	z-score	CN	z-score
NTRK3	4916	246	78,9	-0,98				
NTRK3	4916	247	91,3	-1,29				
NTRK3	4916	248	106,7	0,45				
NYD-SP25	89882	1508	109,9	2,16				
NYD-SP25	89882	1601	94,4	-1,36				
NYD-SP25	89882	1691	82,8	-0,65				
OSR1	9943	625	99,3	-0,95				
OSR1	9943	626	119,0	3,06				
OSR1	9943	627	114,2	0,69				
OSRF	23548	126713	91,8	-0,75				
OSRF	23548	126714	128,4	-0,68				
OSRF	23548	126715	93,9	-0,03				
P101-PI3K	23533	140138	93,8	0,84				
P101-PI3K	23533	140139	139,5	-0,32				
P101-PI3K	23533	140140	108,5	1,08				
P15RS	55197	1164	102,3	1,91				
P15RS	55197	1259	81,5	-0,02				
P15RS	55197	1354	96,9	0,89				
PACE-1	57147	103780	93,1	-0,97				
PACE-1	57147	103782	93,7	-0,19				
PACE-1	57147	111074	107,1	-0,58				
PACSIN1	29993	1496	85,5	0,49				
PACSIN1	29993	1589	124,9	-0,10				
PACSIN1	29993	1679	108,7	-1,91				
PACSIN2	11252	136338	96,8	0,31				
PACSIN2	11252	136339	98,8	-1,61				
PACSIN2	11252	136340	105,5	-0,64				
PACSIN3	29763	24054	91,1	-2,01				
PACSIN3	29763	24241	77,0	-1,43				
PACSIN3	29763	134898	110,1	0,01				
PAK1	5058	249	70,2	-0,80				
PAK1	5058	250	117,0	-0,07				
PAK1	5058	251	103,5	0,02				
PAK2	5062	252	96,9	-0,12				
PAK2	5062	110776	109,3	-0,46				
PAK2	5062	110779	101,7	-0,03				
PAK3	5063	255	98,3	-2,31	103,6	-5,50		
PAK3	5063	256	97,0	-0,28				
PAK3	5063	257	117,0	-0,24				
PAK3	5063	242358			87,0	-2,12		
PAK3	5063	242359			106,6	3,42		
PAK4	10298	731	95,0	-0,93				
PAK4	10298	732	109,2	-0,32				
PAK4	10298	733	94,0	0,57				
PAK6	56924	1029	101,0	0,11				
PAK6	56924	1030	114,1	0,72				
PAK6	56924	1031	57,9	1,62				
PAK7	57144	42902	113,8	-0,19				
PAK7	57144	42997	101,3	0,09				
PAK7	57144	43083	103,7	-0,74				
PANK1	53354	1614	72,9	-0,09				
PANK1	53354	1704	93,8	-0,11				
PANK1	53354	103380	89,6	-1,07				
PANK2	80025	131832	76,8	-1,19				
PANK2	80025	131833	112,0	-0,31				
PANK2	80025	131834	107,7	0,28				
PANK3	79646	130622	103,9	-0,15				
PANK3	79646	130623	140,2	2,05				
PANK3	79646	130624	101,9	0,61				
PANK4	55229	132362	90,5	0,75				
PANK4	55229	132363	98,1	-0,87				
PANK4	55229	132364	85,2	0,01				
PAPSS1	9061	693	100,3	0,42				
PAPSS1	9061	694	94,1	-1,28				
PAPSS1	9061	103595	88,8	1,05				
PAPSS2	9060	1463	111,3	-0,32				
PAPSS2	9060	1557	86,0	0,28				
PAPSS2	9060	1647	95,2	-0,64				
PASK	23178	978	61,0	1,48				
PASK	23178	103354	98,3	0,44				
PASK	23178	110953	102,7	-0,82				
PCM1	5108	144220	123,1	-0,61				
PCM1	5108	144221	99,4	-0,43				
PCM1	5108	144222	98,8	0,35				

			Pass 1		Pass 2		Pass 3	
**Gene Name**	**LocusLink ID**	**siRNA ID**	**CN**	**z-score**	**CN**	**z-score**	**CN**	**z-score**
PCTK1	5127	1472	104,6	-0,33				
PCTK1	5127	1566	102,6	0,03				
PCTK1	5127	1656	76,3	-1,82				
PCTK2	5128	258	101,7	-1,87				
PCTK2	5128	259	99,8	-0,83				
PCTK2	5128	260	101,3	0,11				
PCTK3	5129	202294	90,0	-0,47				
PCTK3	5129	202295	99,0	0,63				
PCTK3	5129	202296	92,8	-0,79				
PDGFRA	5156	758	101,2	0,38				
PDGFRA	5156	759	107,2	1,72				
PDGFRA	5156	760	107,8	0,74				
PDGFRB	5159	1446	96,8	0,25				
PDGFRB	5159	1541	104,8	-0,48				
PDGFRB	5159	1631	100,7	-0,55				
PDK1	5163	261	115,8	0,42				
PDK1	5163	103573	76,1	-1,43				
PDK1	5163	103627	85,4	-2,17				
PDK2	5164	264	113,0	-0,96				
PDK2	5164	265	82,1	-0,58				
PDK2	5164	266	119,1	-0,39				
PDK3	5165	674	86,7	-0,46				
PDK3	5165	675	96,5	-0,04				
PDK3	5165	676	117,2	-0,21				
PDK4	5166	267	116,5	1,51				
PDK4	5166	268	98,9	-0,95				
PDK4	5166	269	85,6	0,37				
PDPK1	5170	270	100,2	0,15				
PDPK1	5170	271	92,6	0,35				
PDPK1	5170	272	101,6	0,43				
PDXK	8566	440	110,8	0,37				
PDXK	8566	441	95,2	-0,79				
PDXK	8566	442	99,8	-0,08				
PFKFB1	5207	273	95,9	-0,63				
PFKFB1	5207	274	101,9	-0,56				
PFKFB1	5207	275	95,9	-0,59				
PFKFB2	5208	761	83,6	-0,06				
PFKFB2	5208	762	108,4	-0,27				
PFKFB2	5208	763	108,4	-0,67				
PFKFB3	5209	103323	89,9	-0,43				
PFKFB3	5209	103407	80,4	0,48				
PFKFB3	5209	103489	111,5	0,36				
PFKFB4	5210	547	100,2	-0,30				
PFKFB4	5210	548	122,2	0,19				
PFKFB4	5210	549	112,4	0,70				
PFKL	5211	276	92,9	0,40				
PFKL	5211	277	99,8	-0,65				
PFKL	5211	278	99,2	-0,40				
PFKM	5213	44	68,3	0,61				
PFKM	5213	46	108,0	-0,73				
PFKM	5213	103563	90,1	0,23				
PFKP	5214	279	89,6	-1,22				
PFKP	5214	280	88,7	-0,97				
PFKP	5214	281	113,4	-0,66				
PFTK1	5218	118282	110,7	1,00				
PFTK1	5218	118283	103,7	-0,42				
PFTK1	5218	118284	120,7	0,57				
PGK1	5230	48	115,9	0,26				
PGK1	5230	103299	105,3	2,16				
PGK1	5230	110731	106,9	0,48				
PGK2	5232	1204	92,4	-0,47				
PGK2	5232	1299	106,3	-0,35				
PGK2	5232	1394	92,6	-1,77				
PHKA1	5255	143791	96,4	-1,24				
PHKA1	5255	143792	102,3	0,12				
PHKA1	5255	143793	89,4	-1,04				
PHKA2	5256	143277	109,2	-0,02				
PHKA2	5256	143278	72,4	-1,30				
PHKA2	5256	143279	103,3	-1,69				
PHKB	5257	143280	98,0	-1,44				
PHKB	5257	143281	73,0	-1,89				
PHKB	5257	143282	95,0	-1,04				

Gene Name	LocusLink ID	siRNA ID	Pass 1		Pass 2		Pass 3	
			CN	z-score	CN	z-score	CN	z-score
PHKG1	5260	764	100,6	0,32				
PHKG1	5260	765	93,0	3,07	103,9	5,74		
PHKG1	5260	766	92,9	-0,46				
PHKG1	5260	242394			106,0	-1,88		
PHKG1	5260	242395			115,6	0,19		
PHKG2	5261	110737	68,1	-0,28				
PHKG2	5261	118254	91,5	-0,51				
PHKG2	5261	118293	97,7	-0,28				
PI4K2B	55300	1167	87,5	-0,35				
PI4K2B	55300	1262	53,0	-1,92				
PI4K2B	55300	1357	101,1	-0,56				
PI4KII	55361	1170	103,9	0,19				
PI4KII	55361	1265	116,3	-1,32				
PI4KII	55361	1360	108,3	0,54				
PIK3AP1	118788	129419	102,6	-0,37				
PIK3AP1	118788	129420	78,7	-0,80				
PIK3AP1	118788	129421	103,2	0,30				
PIK3C2A	5286	143796	95,9	0,31				
PIK3C2A	5286	143797	98,9	0,69				
PIK3C2A	5286	143798	105,8	0,45				
PIK3C2B	5287	143799	100,8	-0,65				
PIK3C2B	5287	143800	109,0	1,15				
PIK3C2B	5287	143801	110,3	-0,96				
PIK3C2G	5288	143925	105,5	0,14				
PIK3C2G	5288	143926	102,6	-0,75				
PIK3C2G	5288	143927	109,0	0,75				
PIK3C3	5289	143802	104,8	0,25				
PIK3C3	5289	143803	102,5	1,50				
PIK3C3	5289	143804	110,7	0,68				
PIK3CA	5290	144250	106,6	0,16				
PIK3CA	5290	144251	85,7	0,76				
PIK3CA	5290	144252	98,7	0,75				
PIK3CB	5291	144253	104,7	-0,27				
PIK3CB	5291	144254	121,9	-0,38				
PIK3CB	5291	144255	96,9	2,05				
PIK3CD	5293	143975	98,5	-0,76				
PIK3CD	5293	143976	72,2	-0,91				
PIK3CD	5293	143977	103,6	-1,03				
PIK3CG	5294	143807	100,4	0,34				
PIK3CG	5294	143808	69,6	-0,61				
PIK3CG	5294	143809	96,0	-0,25				
PIK3R1	5295	118085	96,8	-0,71				
PIK3R1	5295	118086	113,1	0,38				
PIK3R1	5295	118087	132,7	0,37				
PIK3R2	5296	143978	91,1	1,27				
PIK3R2	5296	143979	113,8	0,71				
PIK3R2	5296	143980	106,0	0,58				
PIK3R3	8503	139246	100,9	-0,53				
PIK3R3	8503	139247	133,6	0,06				
PIK3R3	8503	139248	111,1	0,91				
PIK3R4	30849	1480	112,9	0,11				
PIK3R4	30849	1574	100,7	0,32				
PIK3R4	30849	103606	105,5	0,17				
PIK4CA	5297	1511	98,7	2,38				
PIK4CA	5297	1604	120,5	0,98				
PIK4CA	5297	1693	114,5	0,14				
PIK4CB	5298	282	80,4	-0,04				
PIK4CB	5298	283	79,0	-0,87				
PIK4CB	5298	284	122,2	-0,81				
PIM1	5292	1447	102,0	0,71				
PIM1	5292	1542	93,9	-0,46				
PIM1	5292	1632	88,9	-1,49				
PIM2	11040	839	104,7	-0,30				
PIM2	11040	840	94,4	0,64				
PIM2	11040	841	105,7	2,60	110,0	0,40		
PIM2	11040	242406			92,0	-0,72		
PIM2	11040	242407			71,3	2,12		
PINK1	65018	1199	111,0	-0,05				
PINK1	65018	1294	50,7	-2,87				
PINK1	65018	103372	111,9	0,08				
PIP5K1A	8394	415	122,0	0,90				
PIP5K1A	8394	103583	114,0	-0,21				
PIP5K1A	8394	118245	81,8	0,47				

Gene Name	LocusLink ID	siRNA ID	Pass 1		Pass 2		Pass 3	
			CN	z-score	CN	z-score	CN	z-score
PIP5K1B	8395	139134	101,3	0,47				
PIP5K1B	8395	139135	110,5	0,88				
PIP5K1B	8395	139136	94,2	0,11				
PIP5K1C	23396	136520	97,6	-0,97				
PIP5K1C	23396	136521	102,2	0,64				
PIP5K1C	23396	136522	94,8	-0,55				
PIP5K2A	5305	616	92,7	0,32				
PIP5K2A	5305	617	125,7	2,00				
PIP5K2A	5305	618	98,8	0,04				
PIP5K2B	8396	1227	106,0	-0,65				
PIP5K2B	8396	1322	119,5	0,73				
PIP5K2B	8396	110806	121,9	0,00				
PIP5K2C	79837	1191	105,0	-0,06				
PIP5K2C	79837	1286	82,3	1,39				
PIP5K2C	79837	1381	69,9	-1,00				
PIP5KL1	138429	40615	93,9	0,23				
PIP5KL1	138429	40705	100,5	-0,28				
PIP5KL1	138429	46550	110,0	0,58				
PKIA	5569	143005	99,8	0,41				
PKIA	5569	143006	88,4	-0,28				
PKIA	5569	143007	125,6	1,22				
PKIB	5570	34178	103,1	-0,40				
PKIB	5570	34263	74,2	-1,28				
PKIB	5570	143137	104,9	0,26				
PKIG	11142	19143	108,2	-0,09				
PKIG	11142	19235	65,2	0,02				
PKIG	11142	136230	100,3	-1,17				
PKLR	5313	53	101,8	0,74				
PKLR	5313	54	93,5	0,51				
PKLR	5313	55	100,6	0,03				
PKM2	5315	285	100,6	-0,08				
PKM2	5315	286	85,1	1,04				
PKM2	5315	287	109,8	1,54				
PKMYT1	9088	41898	108,9	2,25				
PKMYT1	9088	41988	81,9	-1,27				
PKMYT1	9088	118297	115,2	-0,66				
PKN1	5585	312	105,6	0,26				
PKN1	5585	314	101,4	0,29				
PKN1	5585	111207	97,3	0,39				
PKN2	5586	779	97,1	0,15				
PKN2	5586	780	99,6	0,40				
PKN2	5586	781	92,0	-0,37				
PKN3	29941	909	120,8	0,89				
PKN3	29941	910	91,9	1,61				
PKN3	29941	911	97,3	-0,55				
PLK1	5347	1341	89,3	0,17				
PLK1	5347	42856	102,4	1,09				
PLK1	5347	103548	81,5	-0,47				
PLK2	10769	827	98,1	-0,04				
PLK2	10769	828	107,6	1,25				
PLK2	10769	829	114,5	1,62				
PLK3	1263	103318	113,5	2,08				
PLK3	1263	103806	93,6	-0,34				
PLK3	1263	103809	94,7	-2,65	112,2	-2,91		
PLK3	1263	242380			85,8	-5,13		
PLK3	1263	242381			102,6	-1,38		
PMVK	10654	818	91,7	1,58				
PMVK	10654	819	90,3	-0,49				
PMVK	10654	820	112,1	-0,03				
PNCK	139728	124469	79,0	-0,11				
PNCK	139728	202319	90,0	-0,69				
PNCK	139728	202347	90,2	0,52				
PNKP	11284	860	118,0	2,88				
PNKP	11284	861	109,1	0,07				
PNKP	11284	103345	112,1	0,67				
PRKAA1	5562	767	99,6	-2,71	112,7	-0,12		
PRKAA1	5562	768	97,0	0,03				
PRKAA1	5562	769	115,6	0,50				
PRKAA1	5562	242396			121,0	2,17		
PRKAA1	5562	242397			89,6	0,42		
PRKAA2	5563	772	100,4	-0,35				
PRKAA2	5563	103599	92,7	-0,88				
PRKAA2	5563	103652	81,8	-0,43				

Gene Name	LocusLink ID	siRNA ID	Pass 1		Pass 2		Pass 3	
			CN	z-score	CN	z-score	CN	z-score
PRKAB1	5564	142927	98,5	1,51				
PRKAB1	5564	142928	120,2	1,58				
PRKAB1	5564	142929	127,0	1,60				
PRKAB2	5565	142837	97,7	0,40				
PRKAB2	5565	142838	122,1	0,22				
PRKAB2	5565	142839	108,2	1,61				
PRKACA	5566	292	109,1	-0,04				
PRKACA	5566	293	101,7	-0,57				
PRKACA	5566	103574	86,8	-0,89				
PRKACB	5567	294	92,7	-0,49				
PRKACB	5567	295	73,5	-1,87				
PRKACB	5567	118253	131,4	-0,05				
PRKACG	5568	297	119,4	0,64				
PRKACG	5568	298	95,1	-0,84				
PRKACG	5568	299	92,2	0,00				
PRKAG1	5571	11916	98,1	0,03				
PRKAG1	5571	12010	70,8	0,22				
PRKAG1	5571	12098	84,6	-0,43				
PRKAG2	51422	134877	93,8	-0,36				
PRKAG2	51422	134878	97,3	2,66	81,5	6,90		
PRKAG2	51422	134879	99,9	-1,08				
PRKAG2	51422	242434			104,3	1,49		
PRKAG2	51422	242435			88,9	0,31		
PRKAG3	53632	24967	92,5	0,81				
PRKAG3	53632	25060	98,0	-0,63				
PRKAG3	53632	131929	121,7	-0,26				
PRKAR1A	5573	42857	103,8	-0,58				
PRKAR1A	5573	44836	138,8	-0,39				
PRKAR1A	5573	44930	121,1	-0,49				
PRKAR1B	5575	142277	100,8	0,52				
PRKAR1B	5575	142278	153,6	1,54				
PRKAR1B	5575	142279	112,0	0,72				
PRKAR2A	5576	490	87,3	-0,81				
PRKAR2A	5576	491	94,0	-0,66				
PRKAR2A	5576	492	109,4	-1,02				
PRKAR2B	5577	142280	101,9	-0,72				
PRKAR2B	5577	142281	95,8	-0,27				
PRKAR2B	5577	142282	100,3	-0,13				
PRKCA	5578	300	105,8	-0,24				
PRKCA	5578	301	105,6	1,19				
PRKCA	5578	302	107,3	-0,49				
PRKCABP	9463	138239	100,5	0,84				
PRKCABP	9463	138240	108,0	0,93				
PRKCABP	9463	138241	99,6	0,48				
PRKCB1	5579	304	129,0	-0,28				
PRKCB1	5579	103309	87,4	0,52				
PRKCB1	5579	103396	97,2	-0,32				
PRKCBP1	23613	135457	110,5	-0,69				
PRKCBP1	23613	135458	143,4	1,05				
PRKCBP1	23613	135459	117,0	0,57				
PRKCD	5580	774	115,4	-0,28				
PRKCD	5580	775	52,2	1,21				
PRKCD	5580	103600	86,9	-1,09				
PRKCDBP	112464	45706	97,7	1,04				
PRKCDBP	112464	129307	93,8	-0,40				
PRKCDBP	112464	129308	60,1	-2,01				
PRKCE	5581	677	88,8	0,18				
PRKCE	5581	678	113,4	0,62				
PRKCE	5581	679	109,8	0,40				
PRKCG	5582	306	96,8	-0,38				
PRKCG	5582	307	115,6	-0,20				
PRKCG	5582	308	103,1	-0,93				
PRKCH	5583	777	98,4	1,99				
PRKCH	5583	778	100,4	-0,49				
PRKCH	5583	103822	97,8	-1,26				
PRKCI	5584	309	106,3	2,41	103,0	0,54		
PRKCI	5584	311	88,1	2,12	116,2	7,90	116,5	2,18
PRKCI	5584	110781	88,9	0,77				
PRKCI	5584	242360			102,2	6,98	89,2	2,55
PRKCI	5584	242361			109,0	2,17		
PRKCM	5587	103310	96,7	0,26				
PRKCM	5587	103397	103,6	0,22				
PRKCM	5587	103479	99,8	-0,28				

Gene Name	LocusLink ID	siRNA ID	Pass 1 CN	Pass 1 z-score	Pass 2 CN	Pass 2 z-score	Pass 3 CN	Pass 3 z-score
PRKCN	23683	727	98,0	-0,67				
PRKCN	23683	103336	108,4	-0,33				
PRKCN	23683	103420	96,8	0,94				
PRKCQ	5588	782	101,4	-0,58				
PRKCQ	5588	783	111,3	-0,42				
PRKCQ	5588	784	106,9	1,52				
PRKCSH	5589	11918	103,0	0,51				
PRKCSH	5589	12012	123,8	0,85				
PRKCSH	5589	12100	102,0	2,34				
PRKCZ	5590	103575	68,1	-3,08	100,9	-5,44	88,6	-6,60
PRKCZ	5590	103629	99,1	-0,82				
PRKCZ	5590	103679	97,4	0,32				
PRKCZ	5590	242362			120,2	-1,22	112,6	-3,55
PRKCZ	5590	242363			112,2	0,08		
PRKD2	25865	1011	93,7	0,35				
PRKD2	25865	1013	92,6	-0,12				
PRKD2	25865	111257	92,0	-0,10				
PRKDC	5591	842	99,8	-1,30				
PRKDC	5591	844	86,6	-0,13				
PRKDC	5591	103604	111,0	0,73				
PRKG1	5592	785	95,5	0,74				
PRKG1	5592	786	98,4	0,71				
PRKG1	5592	787	102,0	0,73				
PRKG2	5593	790	92,7	-0,20				
PRKG2	5593	103337	106,4	0,36				
PRKG2	5593	103421	97,1	-0,96				
PRKR	5610	328	94,0	-0,43				
PRKR	5610	329	92,9	-0,63				
PRKR	5610	42855	101,6	0,17				
PRKRA	8575	449	95,0	0,07				
PRKRA	8575	450	100,0	0,46				
PRKRA	8575	451	100,9	-0,76				
PRKRIR	5612	142801	101,0	0,06				
PRKRIR	5612	142802	114,7	0,14				
PRKRIR	5612	142803	64,8	-1,45				
PRKWNK1	65125	1174	86,5	-0,64				
PRKWNK1	65125	1269	98,0	-2,08	115,9	-4,54	110,5	-4,28
PRKWNK1	65125	1364	108,6	0,14				
PRKWNK1	65125	242450			104,9	-3,77	74,0	-5,39
PRKWNK1	65125	242451			114,6	-1,33		
PRKWNK2	65268	130209	103,8	0,75				
PRKWNK2	65268	130210	78,4	0,98				
PRKWNK2	65268	130211	114,9	0,63				
PRKWNK3	65267	103724	122,9	0,19				
PRKWNK3	65267	103732	109,4	-0,30				
PRKWNK3	65267	103740	101,5	-0,66				
PRKWNK4	65266	1104	102,0	0,01				
PRKWNK4	65266	1105	104,4	-1,24				
PRKWNK4	65266	1106	74,6	-0,85				
PRKX	5613	1151	102,2	0,82				
PRKX	5613	1247	90,0	-0,06				
PRKX	5613	1342	102,9	-0,53				
PRKY	5616	332	106,0	-0,14				
PRKY	5616	103312	103,2	-0,05				
PRKY	5616	103805	106,5	0,10				
PRPF4B	8899	460	98,6	-1,62				
PRPF4B	8899	461	91,8	1,36				
PRPF4B	8899	462	100,8	1,65				
PRPS1	5631	335	89,4	-0,66				
PRPS1	5631	111211	107,7	-1,69				
PRPS1	5631	118242	100,9	0,22				
PRPS2	5634	336	78,1	-0,55				
PRPS2	5634	337	80,0	-0,88				
PRPS2	5634	338	81,4	-1,78				
PSKH1	5681	1476	95,5	-0,06				
PSKH1	5681	1570	105,3	-0,30				
PSKH1	5681	1660	105,6	-0,47				
PSKH2	85481	1119	102,8	-0,52				
PSKH2	85481	1120	76,5	-0,68				
PSKH2	85481	1121	97,4	0,38				
PTK2	5747	708	94,2	-0,34				
PTK2	5747	103596	102,2	0,96				
PTK2	5747	103649	102,7	-0,68				

Gene Name	LocusLink ID	siRNA ID	Pass 1		Pass 2		Pass 3	
			CN	z-score	CN	z-score	CN	z-score
PTK2B	2185	484	109,4	-0,90				
PTK2B	2185	103319	93,1	-0,64				
PTK2B	2185	103403	99,4	0,60				
PTK6	5753	740	99,2	-0,96	93,5	-0,21		
PTK6	5753	741	112,3	2,52	96,4	5,29		
PTK6	5753	742	97,6	1,77	103,1	7,74		
PTK7	5754	339	99,9	-0,40				
PTK7	5754	340	106,3	-0,56				
PTK7	5754	341	82,7	-0,27				
PTK9	5756	342	95,1	1,02				
PTK9	5756	343	71,2	-1,15				
PTK9	5756	344	98,0	-0,02				
PTK9L	11344	868	112,4	0,30				
PTK9L	11344	118279	108,1	0,00				
PTK9L	11344	118281	106,1	-0,46				
PXK	54899	1585	88,8	0,71				
PXK	54899	1675	95,2	0,62				
PXK	54899	110964	106,4	0,36				
RAF1	5894	1548	103,1	1,48				
RAF1	5894	1638	112,4	-0,11				
RAF1	5894	42858	110,5	1,81				
RAGE	5891	924	89,3	3,28	71,3	2,37		
RAGE	5891	925	89,1	0,48				
RAGE	5891	926	84,4	-0,20				
RAGE	5891	242421			73,7	2,67		
RAGE	5891	242422			121,8	1,03		
RBKS	64080	1182	98,2	-1,34				
RBKS	64080	1277	126,6	0,10				
RBKS	64080	1372	116,2	0,84				
RET	5979	1435	76,1	0,39				
RET	5979	1530	88,6	-0,05				
RET	5979	1620	94,0	-0,50				
RFK	55312	1168	127,4	0,37				
RFK	55312	1358	126,7	0,43				
RFK	55312	110971	96,7	0,40				
RIOK1	83732	44297	69,1	-0,32				
RIOK1	83732	44389	81,7	0,26				
RIOK1	83732	44477	101,5	-0,10				
RIOK2	55781	1022	77,3	0,68				
RIOK2	55781	110975	106,3	0,59				
RIOK2	55781	110977	74,1	-1,51				
RIOK3	8780	1232	97,7	-1,42				
RIOK3	8780	1327	86,4	0,17				
RIOK3	8780	1422	102,5	0,48				
RIPK1	8737	452	110,0	0,56				
RIPK1	8737	453	108,5	-1,62				
RIPK1	8737	454	103,9	-0,06				
RIPK2	8767	455	97,5	-0,55				
RIPK2	8767	456	92,8	-1,20				
RIPK2	8767	103585	103,0	-0,10				
RIPK3	11035	836	99,9	0,28				
RIPK3	11035	837	106,7	1,44				
RIPK3	11035	110923	108,3	-0,17				
RIPK4	54101	1177	91,6	-0,59				
RIPK4	54101	1272	107,7	-0,52				
RIPK4	54101	113613	86,9	1,19				
RNASEL	6041	1179	108,0	0,91				
RNASEL	6041	1274	80,2	-1,04				
RNASEL	6041	1369	107,4	-1,21				
ROCK1	6093	680	84,7	-0,16				
ROCK1	6093	681	98,4	0,52				
ROCK1	6093	682	100,4	0,25				
ROCK2	9475	595	101,9	0,27				
ROCK2	9475	596	106,4	0,05				
ROCK2	9475	110867	112,9	0,08				
ROR1	4919	613	103,3	0,44				
ROR1	4919	614	103,9	1,19				
ROR1	4919	615	86,6	-2,01				
ROR2	4920	541	100,6	0,58				
ROR2	4920	542	106,1	-0,16				
ROR2	4920	543	106,0	0,37				
ROS1	6098	353	91,0	-0,66				
ROS1	6098	110795	106,8	0,51				
ROS1	6098	110797	101,0	1,19				

Gene Name	LocusLink ID	siRNA ID	Pass 1 CN	Pass 1 z-score	Pass 2 CN	Pass 2 z-score	Pass 3 CN	Pass 3 z-score
RPS6KA1	6195	354	103,5	1,33				
RPS6KA1	6195	355	107,9	0,11				
RPS6KA1	6195	356	104,7	-0,80				
RPS6KA2	6196	1053	102,5	0,00				
RPS6KA2	6196	1054	67,9	-1,48				
RPS6KA2	6196	1055	105,4	0,64				
RPS6KA3	6197	553	98,4	-0,19				
RPS6KA3	6197	554	107,4	0,01				
RPS6KA3	6197	555	104,7	0,60				
RPS6KA4	8986	463	90,4	0,49				
RPS6KA4	8986	464	105,3	0,01				
RPS6KA4	8986	103586	100,2	0,43				
RPS6KA5	9252	580	94,7	-1,02				
RPS6KA5	9252	581	103,8	0,15				
RPS6KA5	9252	103592	107,0	-0,52				
RPS6KA6	27330	945	97,4	-0,12				
RPS6KA6	27330	946	111,6	2,11				
RPS6KA6	27330	947	97,6	0,13				
RPS6KB1	6198	1454	100,4	0,53				
RPS6KB1	6198	1639	105,4	0,02				
RPS6KB1	6198	110802	101,5	0,57				
RPS6KB2	6199	469	107,2	1,01				
RPS6KB2	6199	471	102,4	-0,32				
RPS6KC1	26750	890	95,6	-0,23				
RPS6KC1	26750	891	99,2	-0,11				
RPS6KC1	26750	892	110,4	-0,39				
RPS6KL1	83694	1198	99,2	0,53				
RPS6KL1	83694	1293	105,8	-1,05				
RPS6KL1	83694	1388	103,8	0,18				
RYK	6259	357	92,4	-1,09				
RYK	6259	358	94,6	0,14				
RYK	6259	103579	80,7	0,01				
SCAP1	8631	139355	89,8	-0,41				
SCAP1	8631	139356	127,1	-0,56				
SCAP1	8631	139357	89,9	0,02				
SCGB2A1	4246	44821	88,0	-0,31				
SCGB2A1	4246	143538	114,4	-0,40				
SCGB2A1	4246	143539	96,7	2,61	103,8	6,26	126,1	1,11
SCGB2A1	4246	242352			97,1	7,90	97,6	2,92
SCGB2A1	4246	242353			117,2	5,87	108,6	-0,55
SCYL1	57410	1050	96,0	-0,99				
SCYL1	57410	1051	98,4	0,92				
SCYL1	57410	1052	105,7	-0,69				
SEPHS1	22929	878	97,6	-1,77				
SEPHS1	22929	879	82,0	0,05				
SEPHS1	22929	880	104,2	0,06				
SEPHS2	22928	881	94,6	0,35				
SEPHS2	22928	882	107,9	-0,01				
SEPHS2	22928	883	115,4	-0,24				
SGK	6446	711	96,5	-0,44				
SGK	6446	103597	110,2	1,19				
SGK	6446	103650	101,2	-0,24				
SGK2	10110	1485	104,8	-2,08	100,0	-5,64	97,3	-6,82
SGK2	10110	1579	106,7	-0,98	109,4	-2,81	119,5	-5,15
SGK2	10110	1669	97,5	-0,08	89,8	-7,57	76,4	-6,32
SGK2	10110	242482			97,4	0,57		
SGK2	10110	242483			99,4	0,94		
SGKL	23678	1479	97,8	0,29				
SGKL	23678	1573	108,6	0,09				
SGKL	23678	1663	110,8	0,66				
SH3KBP1	30011	135342	99,1	1,28				
SH3KBP1	30011	135343	95,8	-0,01				
SH3KBP1	30011	135344	57,2	-1,40				
SIK2	23235	122759	106,5	-0,54				
SIK2	23235	122760	107,1	0,96				
SIK2	23235	122761	102,0	-1,56				
SKIV2L	6499	143038	105,4	0,26				
SKIV2L	6499	143039	109,9	-0,46				
SKIV2L	6499	143040	124,6	0,88				
SKP2	6502	142899	100,7	-0,66				
SKP2	6502	142900	108,5	0,51				
SKP2	6502	142901	112,6	-0,43				
SLK	9748	957	99,7	-0,83				
SLK	9748	958	105,8	0,04				
SLK	9748	959	75,3	-0,64				

Gene Name	LocusLink ID	siRNA ID	Pass 1 CN	Pass 1 z-score	Pass 2 CN	Pass 2 z-score	Pass 3 CN	Pass 3 z-score
SMG1	23049	44241	88,6	0,99				
SMG1	23049	44334	104,5	-0,61				
SMG1	23049	44424	104,9	0,08				
SNARK	81788	1083	106,5	0,77				
SNARK	81788	1084	101,8	3,14	110,9	3,08		
SNARK	81788	103368	102,3	0,20				
SNARK	81788	242464			96,8	0,27		
SNARK	81788	242465			95,0	5,88		
SNF1LK	150094	40587	136,3	0,43				
SNF1LK	150094	40679	104,5	1,72				
SNF1LK	150094	40762	110,6	0,85				
SNRK	54861	1159	102,1	0,87				
SNRK	54861	1349	103,5	0,30				
SNRK	54861	103607	108,1	-1,47				
SPHK1	8877	1181	97,2	-0,02				
SPHK1	8877	1276	86,0	-0,40				
SPHK1	8877	1371	42,2	-1,48				
SPHK2	56848	1587	87,0	-0,98				
SPHK2	56848	1677	92,0	-0,10				
SPHK2	56848	103792	97,4	1,02				
SRC	6714	683	104,2	-0,80				
SRC	6714	684	97,6	-0,41				
SRC	6714	103333	104,7	0,91				
SRMS	6725	1131	91,8	0,81				
SRMS	6725	1132	99,9	-0,98				
SRMS	6725	1133	98,1	0,80				
SRPK1	6732	365	113,2	0,68				
SRPK1	6732	366	113,8	-0,03				
SRPK1	6732	367	68,8	0,57				
SRPK2	6733	103743	111,4	-0,05				
SRPK2	6733	103749	84,1	1,70				
SRPK2	6733	103752	139,6	1,18				
SSTK	83983	42598	102,2	0,35				
SSTK	83983	42691	99,7	-0,98				
SSTK	83983	42768	102,3	0,10				
STK10	6793	743	69,5	-1,29				
STK10	6793	744	100,3	-0,19				
STK10	6793	745	60,9	0,62				
STK11	6794	59	111,7	0,65				
STK11	6794	60	106,4	0,35				
STK11	6794	61	100,3	-0,84				
STK11IP	114790	128863	108,0	-1,02				
STK11IP	114790	128864	136,3	-0,38				
STK11IP	114790	128865	122,8	0,31				
STK16	8576	1457	88,1	-3,26	92,8	-5,32		
STK16	8576	1552	117,2	0,58				
STK16	8576	103584	71,4	-1,33				
STK16	8576	242342			83,9	-1,09		
STK16	8576	242343			100,0	3,11		
STK17A	9263	585	94,2	1,04				
STK17A	9263	110861	127,4	2,08				
STK17A	9263	110863	61,7	-0,95				
STK17B	9262	498	104,9	-0,41				
STK17B	9262	103588	95,5	-0,03				
STK17B	9262	118265	107,9	-0,03				
STK18	10733	103348	109,0	-0,22				
STK18	10733	103432	94,6	3,03	91,7	4,49		
STK18	10733	103512	117,4	0,85				
STK18	10733	242423			138,3	-0,92		
STK18	10733	242424			84,3	1,72		
STK19	8859	1504	95,2	-0,92				
STK19	8859	1597	100,3	1,15				
STK19	8859	1687	110,8	0,39				
STK22B	23617	46442	97,7	-1,25				
STK22B	23617	103793	107,3	-0,46				
STK22B	23617	103798	89,9	1,49				
STK22C	81629	1122	74,9	-1,01				
STK22C	81629	1123	95,5	0,03				
STK22C	81629	1124	102,3	-1,20				
STK22D	83942	1097	102,0	-0,26				
STK22D	83942	111259	87,4	-1,19				
STK22D	83942	118249	90,3	-1,75				

Gene Name	LocusLink ID	siRNA ID	Pass 1		Pass 2		Pass 3	
			CN	z-score	CN	z-score	CN	z-score
STK23	26576	936	84,6	-0,21				
STK23	26576	937	80,6	-1,30				
STK23	26576	938	122,4	3,83	112,4	1,55		
STK23	26576	242425			89,6	0,06		
STK23	26576	242426			101,7	0,55		
STK24	8428	419	96,8	0,21				
STK24	8428	420	106,6	0,50				
STK24	8428	421	69,4	-0,73				
STK25	10494	812	105,7	-0,47				
STK25	10494	813	86,5	-0,19				
STK25	10494	814	95,1	0,36				
STK29	9024	13849	100,1	-0,24				
STK29	9024	13942	106,5	0,20				
STK29	9024	14032	92,9	-0,83				
STK3	6788	791	103,0	-0,35				
STK3	6788	792	99,6	0,43				
STK3	6788	103601	90,1	-1,20				
STK31	56164	1594	116,5	0,20				
STK31	56164	1684	109,4	0,55				
STK31	56164	103369	91,4	1,90				
STK32B	55351	1023	90,5	-2,64				
STK32B	55351	1024	111,2	-0,14				
STK32B	55351	1025	101,5	-0,30				
STK32C	282974	40863	104,8	0,13				
STK32C	282974	45762	94,8	-1,39				
STK32C	282974	45854	96,4	0,58				
STK33	65975	1683	95,7	-0,56				
STK33	65975	103367	103,1	0,33				
STK33	65975	103451	106,4	0,51				
STK35	140901	1135	109,8	-1,03	110,5	-3,59	88,5	-5,36
STK35	140901	103377	101,9	0,72	89,9	-4,05	89,2	-3,95
STK35	140901	103461	98,0	-0,21	84,9	-3,61	102,8	-3,70
STK36	27148	1482	101,6	-1,35				
STK36	27148	1576	99,1	1,13				
STK36	27148	1666	113,9	2,13				
STK38	11329	863	68,1	0,78				
STK38	11329	864	107,7	-0,30				
STK38	11329	865	99,7	-0,37				
STK38L	23012	1481	95,8	-0,47				
STK38L	23012	1575	102,8	-0,59				
STK38L	23012	1665	96,6	0,34				
STK39	27347	896	105,2	-0,60				
STK39	27347	897	102,7	1,09				
STK39	27347	897	106,0	0,14				
STK39	27347	898	99,6	0,15				
STK4	6789	794	92,8	0,93	110,6	3,53		
STK4	6789	795	77,1	1,81	115,9	1,93		
STK4	6789	103602	97,7	-1,50				
STK4	6789	242398			94,7	-1,19		
STK4	6789	242399			98,8	1,01		
STK6	6790	425	113,3	1,34				
STK6	6790	426	111,2	-0,48				
STK6	6790	427	66,8	-1,20				
STYK1	55359	1169	107,7	0,98				
STYK1	55359	1264	92,2	-0,67				
STYK1	55359	1359	97,3	-2,23	127,5	4,05		
STYK1	55359	242448			122,6	7,39		
STYK1	55359	242449			109,1	-0,27		
SYK	6850	380	109,7	0,18				
SYK	6850	381	121,7	1,42				
SYK	6850	382	97,9	-1,01				
T3JAM	80342	123566	93,2	-0,32				
T3JAM	80342	123567	102,7	1,60				
T3JAM	80342	123568	114,9	1,43				
TAF1	6872	103547	110,6	0,39				
TAF1	6872	103553	62,7	-1,91				
TAF1	6872	103559	103,4	0,65				
TAF1L	138474	40303	117,4	0,70				
TAF1L	138474	40394	101,0	-0,64				
TAF1L	138474	40482	113,0	-0,73				
TAO1	9344	118285	107,8	1,21				
TAO1	9344	118286	101,2	0,64				
TAO1	9344	118287	130,3	0,92				

Gene Name	LocusLink ID	siRNA ID	Pass 1 CN	Pass 1 z-score	Pass 2 CN	Pass 2 z-score	Pass 3 CN	Pass 3 z-score
TBK1	29110	899	101,8	0,61				
TBK1	29110	900	89,0	-0,63				
TBK1	29110	901	94,4	-0,71				
TEC	7006	383	107,8	0,71				
TEC	7006	384	96,6	0,02				
TEC	7006	385	97,0	-1,65				
TEK	7010	64	101,1	-0,22				
TEK	7010	103300	92,1	-0,09				
TEK	7010	103387	107,2	1,11				
TESK1	7016	797	98,9	-0,71				
TESK1	7016	798	114,1	0,42				
TESK1	7016	799	97,5	3,05	128,8	6,87		
TESK1	7016	242400			73,2	-4,12		
TESK1	7016	242401			112,9	-1,94		
TESK2	10420	852	91,5	0,11				
TESK2	10420	853	90,3	2,68	82,3	1,18		
TESK2	10420	110925	87,5	-1,35				
TESK2	10420	242408			95,3	-2,22		
TEX14	56155	1086	104,0	-0,06				
TEX14	56155	1087	113,7	0,02				
TEX14	56155	1088	94,6	-1,96				
TGFBR1	7046	556	83,8	-1,88				
TGFBR1	7046	557	98,1	-0,98				
TGFBR1	7046	103324	107,7	-0,06				
TGFBR2	7048	386	92,3	-0,21				
TGFBR2	7048	387	104,0	-0,44				
TGFBR2	7048	388	105,4	1,39				
TIE	7075	686	111,1	0,97				
TIE	7075	687	103,2	-0,90				
TIE	7075	688	98,3	0,21				
TIF1	8805	103317	59,6	-2,52				
TIF1	8805	103402	79,0	-1,40				
TIF1	8805	103484	96,3	0,49				
TK1	7083	389	106,9	-0,20				
TK1	7083	390	111,7	-0,03				
TK1	7083	391	83,5	0,40				
TK2	7084	559	91,0	0,72				
TK2	7084	560	76,6	1,49				
TK2	7084	561	107,0	-0,61				
TLK1	9874	884	81,6	-2,82	91,8	-5,82		
TLK1	9874	885	105,0	2,91				
TLK1	9874	886	110,3	-0,17				
TLK1	9874	242415			115,0	-0,89		
TLK1	9874	242416			82,1	-2,05		
TLK2	11011	103342	87,9	0,85				
TLK2	11011	103426	107,5	0,24				
TLK2	11011	103507	73,7	0,46				
TNK1	8711	477	102,9	0,19				
TNK1	8711	110823	112,8	0,66				
TNK1	8711	110827	99,5	0,13				
TNNI3K	51086	987	96,3	-0,04				
TNNI3K	51086	988	98,8	2,89	102,2	8,39		
TNNI3K	51086	989	73,7	-0,28				
TNNI3K	51086	242432			90,4	4,93		
TNNI3K	51086	242433			79,2	1,68		
TOPK	55872	1026	108,2	0,62				
TOPK	55872	1027	99,9	0,70				
TOPK	55872	1028	91,1	-0,62				
TP53RK	112858	1202	102,7	0,33				
TP53RK	112858	1297	99,5	-0,49				
TP53RK	112858	1392	118,2	-0,41				
TPK1	27010	1184	108,2	0,15				
TPK1	27010	1279	90,1	1,36				
TPK1	27010	1374	93,5	0,21				
TRAD	11139	845	106,7	0,29				
TRAD	11139	846	95,7	-0,64				
TRAD	11139	847	109,0	-0,91				
TRIB1	10221	103365	121,8	0,29				
TRIB1	10221	103449	105,9	1,29				
TRIB1	10221	103529	98,8	-0,27				
TRIB2	28951	1061	101,3	1,54				
TRIB2	28951	103364	97,8	-0,22				
TRIB2	28951	103448	102,6	-0,47				

Gene Name	LocusLink ID	siRNA ID	Pass 1 CN	Pass 1 z-score	Pass 2 CN	Pass 2 z-score	Pass 3 CN	Pass 3 z-score
TRIB3	57761	1056	100,1	-0,43				
TRIB3	57761	1057	102,4	0,39				
TRIB3	57761	1058	97,7	-0,25				
TRIM28	10155	716	108,5	-0,94				
TRIM28	10155	717	106,6	1,70				
TRIM28	10155	718	90,4	1,32				
TRIM33	51592	103355	108,8	0,43				
TRIM33	51592	103439	106,3	1,16				
TRIM33	51592	103519	124,2	0,07				
TRIO	7204	849	100,7	-0,66				
TRIO	7204	103343	96,5	0,07				
TRIO	7204	103427	104,2	1,15				
TRPM6	140803	1158	96,5	-0,62				
TRPM6	140803	1253	103,9	1,26				
TRPM6	140803	1348	87,4	-1,14				
TRPM7	54822	1490	104,5	1,22				
TRPM7	54822	103360	71,5	-0,92				
TRPM7	54822	103444	79,2	-1,15				
TRRAP	8295	408	93,8	0,10				
TRRAP	8295	103314	80,3	-2,66	90,2	-7,02		
TRRAP	8295	103399	64,3	-1,27				
TRRAP	8295	242374			77,2	-2,65		
TRRAP	8295	242375			99,2	0,69		
TSKS	60385	125326	101,9	0,95				
TSKS	60385	125327	107,8	1,29				
TSKS	60385	125328	80,6	1,19				
TTBK2	146057	40791	97,3	-0,57				
TTBK2	146057	40887	94,1	-0,41				
TTBK2	146057	40980	87,0	-0,32				
TTK	7272	392	89,3	1,03				
TTK	7272	393	110,2	0,86				
TTK	7272	394	117,1	-0,67				
TTN	7273	1515	101,8	0,21				
TTN	7273	1607	98,0	-0,73				
TTN	7273	1696	102,5	2,89	95,3	3,54		
TTN	7273	242370			116,0	2,25		
TTN	7273	242371			87,8	-4,44		
TXK	7294	395	94,8	-0,94				
TXK	7294	396	103,4	0,43				
TXK	7294	397	102,3	-0,60				
TYK2	7297	398	104,4	-0,17				
TYK2	7297	399	105,4	0,27				
TYK2	7297	400	81,7	1,26				
TYRO3	7301	800	99,7	0,29				
TYRO3	7301	801	101,5	-0,30				
TYRO3	7301	802	94,5	-1,08				
UCK1	83549	103370	98,6	-0,02				
UCK1	83549	103454	106,0	0,18				
UCK1	83549	103534	66,0	-0,82				
UCK2	7371	893	98,3	-0,01				
UCK2	7371	894	93,6	-0,92				
UCK2	7371	895	103,9	-0,54				
UCKL1	54963	1161	100,8	-0,21				
UCKL1	54963	1256	94,4	0,05				
UCKL1	54963	1351	105,7	-1,84				
UGP2	7360	830	106,5	0,33				
UGP2	7360	831	96,1	0,96				
UGP2	7360	832	111,8	-0,19				
ULK1	8408	118259	84,6	-1,34				
ULK1	8408	118260	105,7	0,31				
ULK1	8408	118261	80,4	2,21	101,6	8,45		
ULK1	8408	242376			89,1	-1,93		
ULK1	8408	242377			111,5	1,18		
ULK2	9706	954	74,0	0,60				
ULK2	9706	955	109,7	1,08				
ULK2	9706	956	89,1	2,87	84,0	2,52		
ULK2	9706	242427			100,2	0,21		
UMP-CMPK	51727	1005	104,9	-0,55				
UMP-CMPK	51727	1006	101,7	-0,67				
UMP-CMPK	51727	1007	95,2	0,04				
VRK1	7443	401	91,3	0,40				
VRK1	7443	402	95,3	1,15				
VRK1	7443	403	98,7	-0,88				

Gene Name	LocusLink ID	siRNA ID	Pass 1		Pass 2		Pass 3	
			CN	z-score	CN	z-score	CN	z-score
VRK2	7444	803	93,5	-0,63				
VRK2	7444	804	104,5	0,56				
VRK2	7444	805	97,0	2,83	114,3	8,39		
VRK2	7444	242402			86,3	-2,84		
VRK2	7444	242403			102,3	0,56		
VRK3	51231	1008	96,5	-0,20				
VRK3	51231	1009	92,7	-0,83				
VRK3	51231	1010	102,2	0,15				
WEE1	7465	405	107,2	-0,63				
WEE1	7465	103582	94,6	0,08				
WEE1	7465	103636	53,5	2,98				
XYLB	9942	103329	92,1	0,14				
XYLB	9942	103413	109,6	1,25				
XYLB	9942	103495	115,8	-0,94				
YES1	7525	690	113,3	-0,02				
YES1	7525	103334	108,9	0,27				
YES1	7525	103418	91,1	-0,88				
ZAK	51776	1016	91,4	1,23				
ZAK	51776	103358	100,0	1,45				
ZAK	51776	110959	102,1	-1,11				
ZAP70	7535	103736	93,6	-0,37				
ZAP70	7535	110756	105,1	-0,60				
ZAP70	7535	110759	104,4	1,02				
