# Supplementary material for: Kinome-Wide RNAi Screen Implicates at Least 5 Host Hepatocyte Kinases in Plasmodium Sporozoite Infection
Source: PLoS Pathog. 2008 Nov 7;4(11):e1000201. doi: 10.1371/journal.ppat.1000201 (PMC2574010; doi:10.1371/journal.ppat.1000201)
Supplement: Figure S1 — Infected cells following knock-down of hit genes identified in the RNAi screen. Representative pictures of cells transfected with siRNAs targeting MET, PRKWNK1, SGK2, STK35, PKCζ and a Negative control siRNA, 24 hours after infection with P. berghei sporozoites. The pictures depict nuclei (in blue), actin (in red) and EEFs (in green) and show that cell confluency and morphology are not affected by gene knock-down whereas infection is decreased in all cases. (0.68 MB PDF) [file ppat.1000201.s002.pdf]

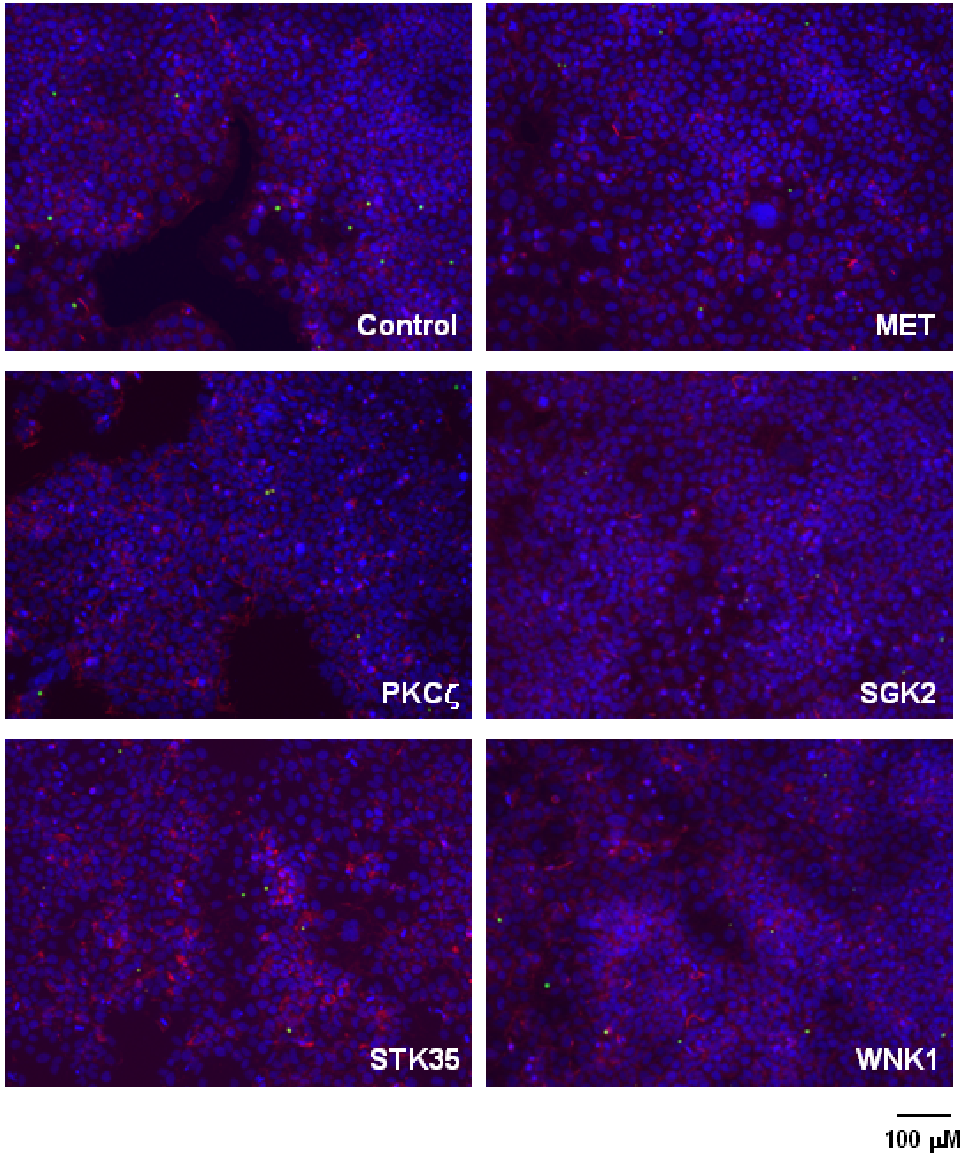

Figure S1. Infected cells following knock-down of hit genes identified in the RNAi screen. Representative pictures of cells transfected with siRNAs targeting MET, PRKWNK1, SGK2, STK35, PKCζ and a Negative control siRNA, 24 hours after infection with *P. berghei* sporozoites. The pictures depict nuclei (in blue), actin (in red) and EEFs (in green) and show that cell confluency and morphology are not affected by gene knock-down whereas infection is decreased in all cases.
