# Supplementary material for: Kinome-Wide RNAi Screen Implicates at Least 5 Host Hepatocyte Kinases in Plasmodium Sporozoite Infection
Source: PLoS Pathog. 2008 Nov 7;4(11):e1000201. doi: 10.1371/journal.ppat.1000201 (PMC2574010; doi:10.1371/journal.ppat.1000201)
Supplement: Figure S2 — RNAi screen data analysed in terms of gene classification and gene ontology. (A) Data analysis according to distribution through kinase and kinase-related families. (B) Data analysis according to gene ontology and molecular function. (C) Gene ontology enrichment analysis of 3 RNAi screens showing only significantly enriched kinases (p<0.05). Spheres on Pass 3 pie plots denote the 5 kinases identified in the RNAi screen. (0.13 MB PDF) [file ppat.1000201.s003.pdf]

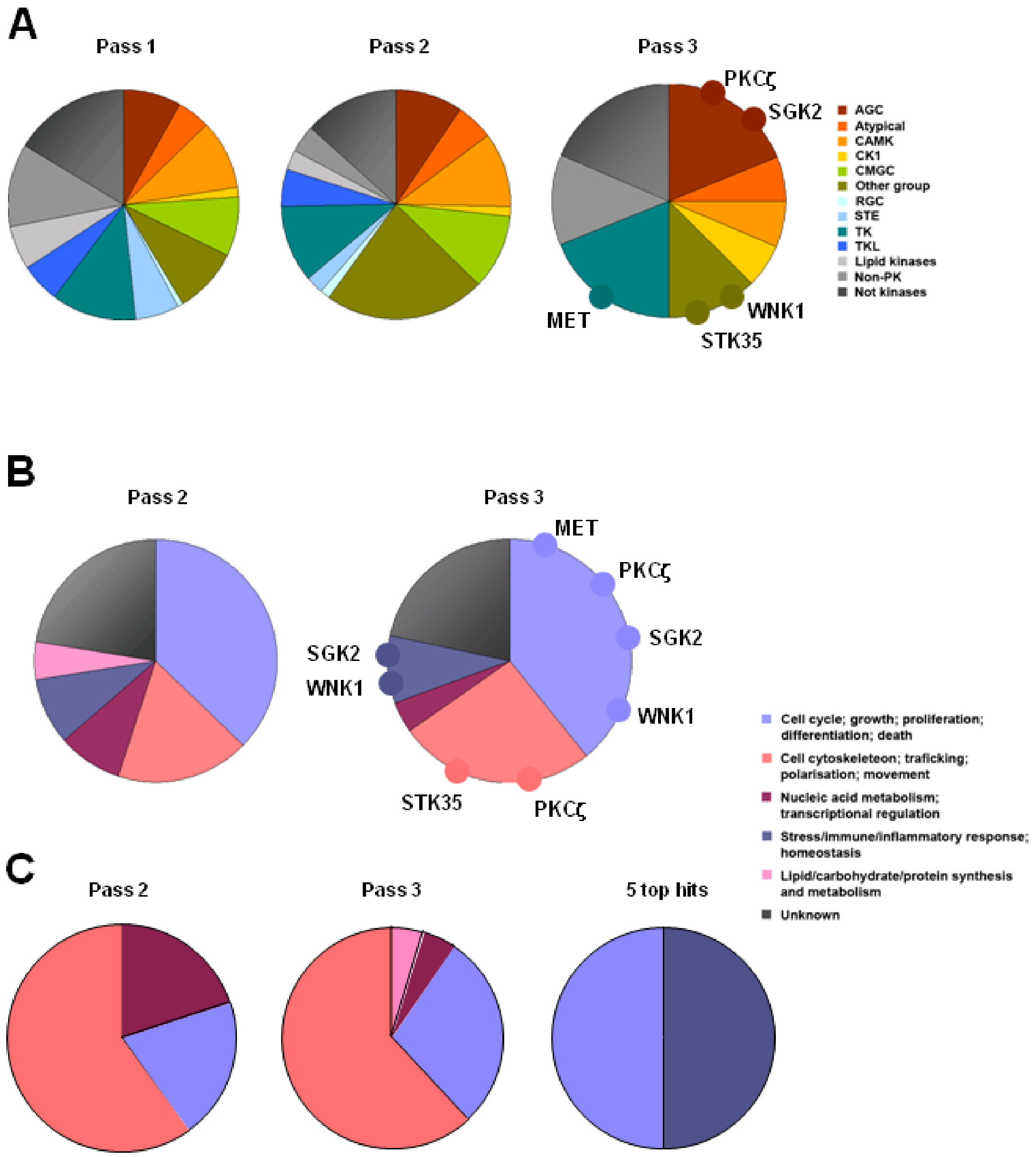

Figure S2. RNAi screen data analysed in terms of gene classification and gene ontology.
(A) Data analysis according to distribution through kinase and kinase-related families.
(B) Data analysis according to gene ontology and molecular function.
(C) Gene ontology enrichment analysis of 3 RNAi screens showing only significantly enriched kinases ($p < 0.05$).
Spheres on Pass 3 pie plots denote the 5 kinases identified in the RNAi screen.
