# Supplementary material for: Kinome-Wide RNAi Screen Implicates at Least 5 Host Hepatocyte Kinases in Plasmodium Sporozoite Infection
Source: PLoS Pathog. 2008 Nov 7;4(11):e1000201. doi: 10.1371/journal.ppat.1000201 (PMC2574010; doi:10.1371/journal.ppat.1000201)
Supplement: Figure S3 — Specificity of inhibition by PKCzInh. (A) Specificity of inhibition of atypical PKCs versus conventional and novel PKCs by PKCzInh. Cells treated with PKCzInh or with an equivalent amount of a scrambled peptide control, were probed with an antibody that specifically recognises the autophosphorilated form of atypical PKCs (Thr560 on PKCζ; Thr555 on PKCι) (top). In parallel, treated cells were incubated with phospho-PKC (pan), an antibody that specifically recognises the autophosphorilated form of conventional and novel PKCs (Ser660 on the hidrophobic site) (bottom). When at least 10 µM PKCzInh were used, a clear decrease in the intensity of the band corresponding to the atypical PKCs is observed while no difference is observed in the bands corresponding to the conventional and novel PKCs, showing that PKCzInh specifically inhibits atypical PKCs and has no effect on other PKCs. (B) Specificity of inhibition between atypical PKCs (PKCζ versus PKCι) by PKCzInh. To distinguish between PKCζ and PKCι, Huh7 cells were transfected with plasmids expressing either a tagged version of PKCζ or a tagged version of PKCι. The tag in either construct enables the overexpressed protein to be distinguished from the endogenous ones on SDS-PAGE, and the degree of autophosphorilation of each of the atypical PKCs to be analysed independently. The expression level of PKCζ is approximately 5-fold higher than that of PKCι, as shown by quantification of signal intensity of the two proteins following probing with an antibody that recognises both isoforms (left). Inhibition on those cells was only observed when 60 µM PKCzInh was used. Inhibition levels of PKCζ (middle) and PKCι (right) were assessed by probing with an antibody that specifically recognises the autophosphorilated form of atypical PKCs. The relative specificities of PKCzInh for the two isoforms were determined by calculating the percentage of inhibition of autophosphorilation of each of them and correcting for the difference in the [file ppat.1000201.s004.pdf]

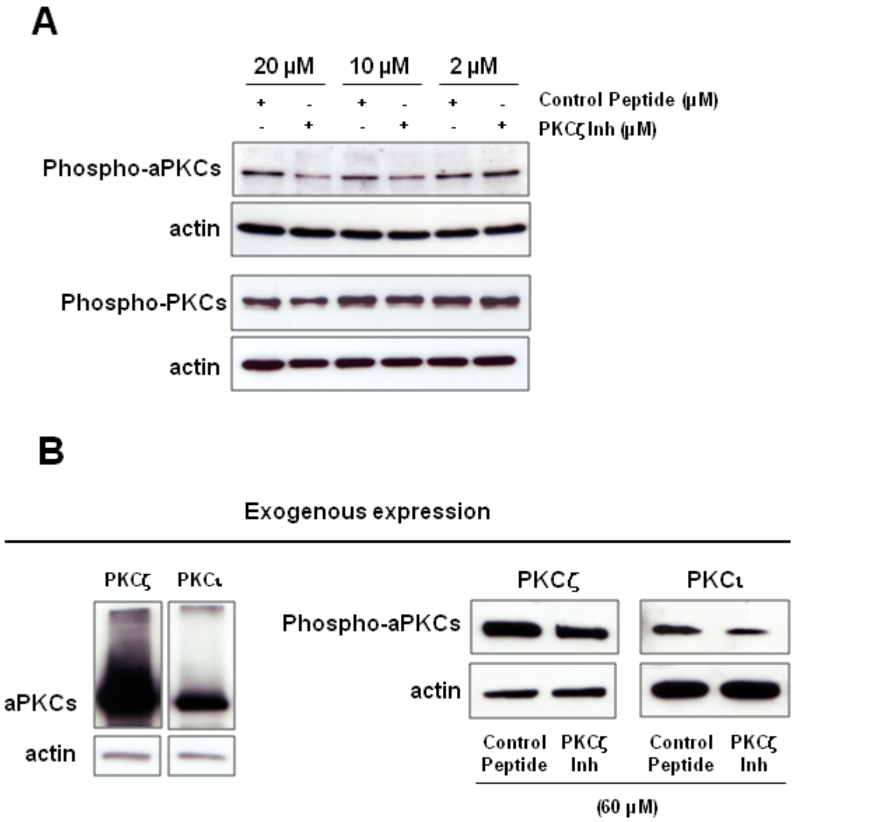


**Figure S3.** Specificity of inhibition by PKCzInh.

(A) Specificity of inhibition of atypical PKCs *versus* conventional and novel PKCs by PKCzInh. Cells treated with PKCzInh or with an equivalent amount of a scrambled peptide control, were probed with an antibody that specifically recognises the autophosphorilated form of atypical PKCs (Thr560 on PKCζ; Thr555 on PKCι) (top). In parallel, treated cells were incubated with phospho-PKC (pan), an antibody that specifically recognises the autophosphorilated form of conventional and novel PKCs (Ser660 on the hidrophobic site) (bottom). When at least 10 µM PKCzInh were used, a clear decrease in the intensity of the band corresponding to the atypical PKCs is observed while no difference is observed in the bands corresponding to the conventional and novel PKCs, showing that PKCzInh specifically inhibits atypical PKCs and has no effect on other PKCs.

(B) Specificity of inhibition between atypical PKCs (PKCζ *versus* PKCι) by PKCzInh.

To distinguish between PKCζ and PKCι, Huh7 cells were transfected with plasmids expressing either a tagged version of PKCζ or a tagged version of PKCι. The tag in either construct enables the overexpressed protein to be distinguished from the endogenous ones on SDS-PAGE, and the degree of autophosphorilation of each of the atypical PKCs to be analysed independently. The expression level of PKCζ is approximately 5-fold higher than that of PKCι, as shown by quantification of signal intensity of the two proteins following probing with an antibody that recognises both isoforms (left). Inhibition on those cells was only observed when 60 µM PKCzInh was used. Inhibition levels of PKCζ (middle) and PKCι (right) were assessed by probing with an antibody that specifically recognises the autophosphorilated form of atypical PKCs. The relative specificities of PKCzInh for the two isoforms were determined by calculating the percentage of inhibition of autophosphorilation of each of them and correcting for the difference in their expression levels. Our results show that the inhibitory effect on PKZζ is approximately 2-fold that observed for PKCι.
