# Supplementary material for: Kinome-Wide RNAi Screen Implicates at Least 5 Host Hepatocyte Kinases in Plasmodium Sporozoite Infection
Source: PLoS Pathog. 2008 Nov 7;4(11):e1000201. doi: 10.1371/journal.ppat.1000201 (PMC2574010; doi:10.1371/journal.ppat.1000201)
Supplement: Figure S4 — PKCζ in vivo knock-down does not cause any detectable or significant signs of Interferon or toxicity response on mouse livers. Effect of siRNA-mediated in vivo knock-down of PKCζ on expression of interferon or toxicity-related genes measured by qRT-PCR. Each individual graph shows expression data for one gene indicated on top. For each siRNA, RNA extracts from liver samples of 3 different mice were tested in duplicate. The same set of samples, taken 48 h after sporozoite i.v. infection was used for the entire data set. Average and SD were normalized to the expression level of Rpl13a as housekeeper. Interferon response markers: Ifna1,interferon alpha1; ifnb1, interferon beta1; Ifi44, interferon-induced protein 44; Ifit1 and 2, interferon-induced protein(s) with tetratricopeptide repeats 1 and 2; Irf7, interferon regulatory factor 7; Mx1, myxovirus resistance 1; Oas2, oligoadenylate synthetase 2; Stat1, signal transducer and activator of transcription 1. Toxicity response markers: Bax, Bcl2-associated X protein; Bcl2l11, BCL2-like 11; Fos, FBJ osteosarcoma oncogene; Fosl1; fos-like antigen 1; Fyb, FYN binding protein; Gadd45a, growth arrest and DNA-damage-inducible 45 alpha; Gapdh, glyceraldehyde-3-phosphate dehydrogenase; Hspa5, heat shock protein 5; Il18, interleukine 18; Jun, Jun oncogene; Mapk3, mitogen activated protein kinase 3; Myc, myelocytomatosis oncogene. (0.07 MB PDF) [file ppat.1000201.s005.pdf]

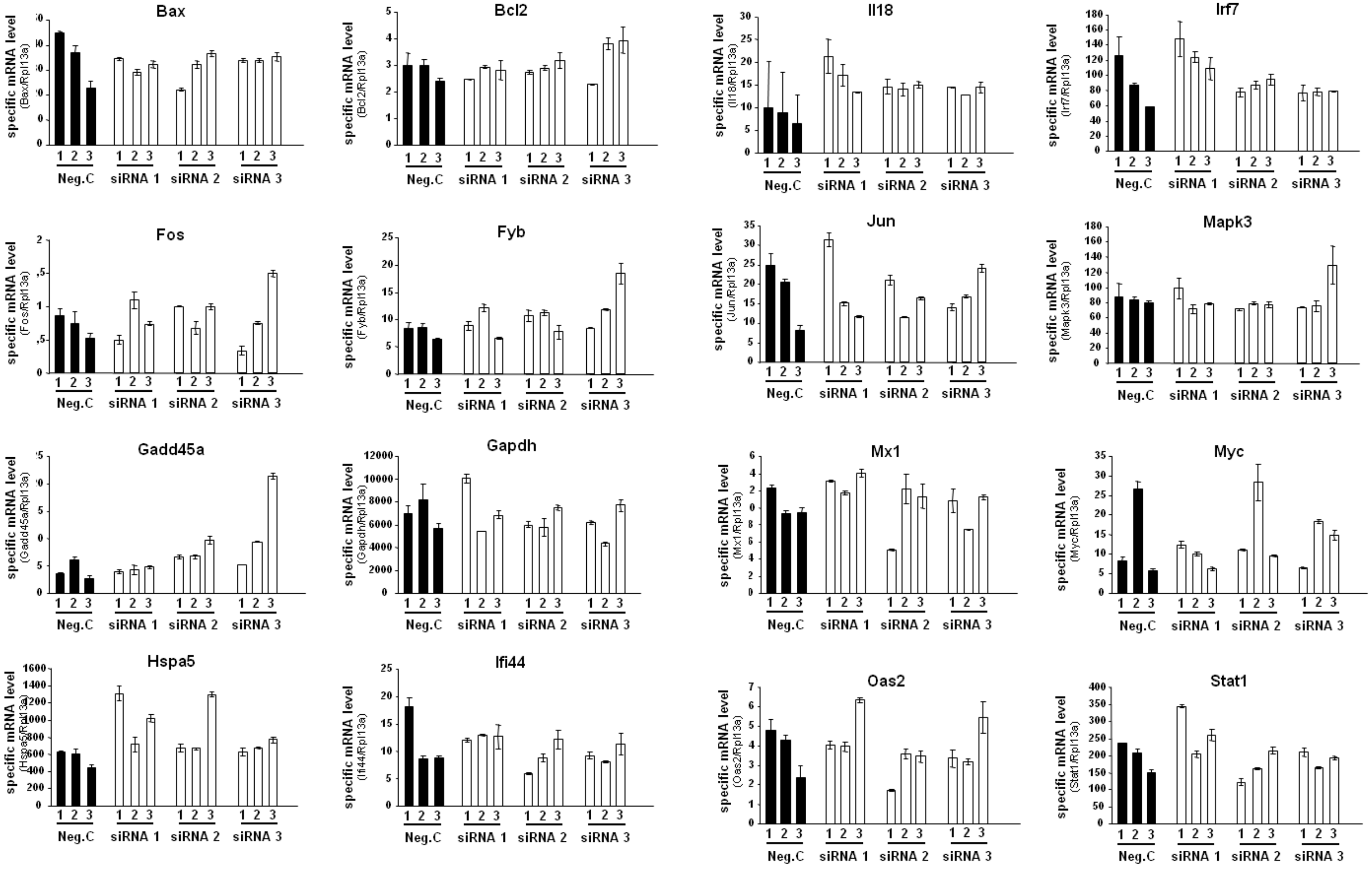

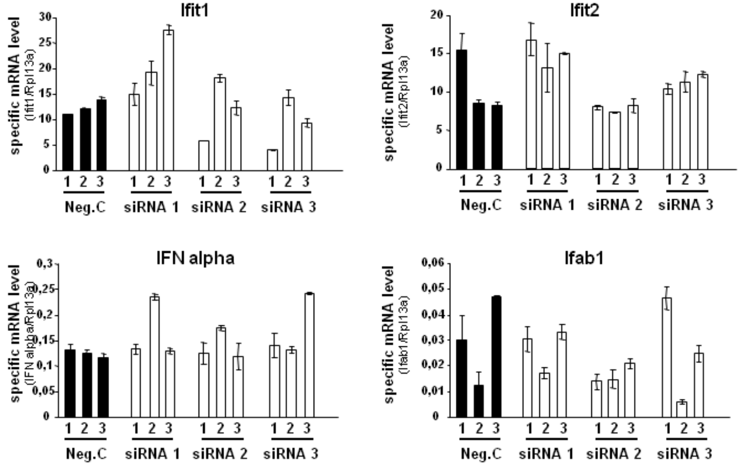

Figure S4. PKCζ *in vivo* knock-down does not cause any detectable or significant signs of Interferon or toxicity response on mouse livers. Effect of siRNA-mediated *in vivo* knock-down of PKCζ on expression of interferon or toxicity-related genes measured by qRT-PCR. Each individual graph shows expression data for one gene indicated on top. For each siRNA, RNA extracts from liver samples of 3 different mice were tested in duplicate. The same set of samples, taken 48h after sporozoite i.v.infection was used for the entire data set. Average and SD were normalized to the expression level of Rpl13a as housekeeper.

Interferon response markers: Ifna1,interferon alpha1; ifnb1, interferon beta1; Ifi44, interferon-induced protein 44; Ifit1 and 2, interferon-induced protein(s) with tetratricopeptide repeats 1 and 2; Irf7, interferon regulatory factor 7; Mx1, myxovirus resistance 1; Oas2, oligoadenylate synthetase 2; Stat1, signal transducer and activator of transcription 1.

Toxicity response markers: Bax, Bcl2-associated X protein; Bcl2l11, BCL2-like 11; Fos, FBJ osteosarcoma oncogene; Fosl1; fos-like antigen 1; Fyb, FYN binding protein; Gadd45a, growth arrest and DNA-damage-inducible 45 alpha; Gapdh, glyceraldehyde-3-phosphate dehydrogenase; Hspa5, heat shock protein 5; Il18, interleukine 18; Jun, Jun oncogene; Mapk3, mitogen activated protein kinase 3; Myc, myelocytomatosis oncogene.
