# Supplementary material for: Kinome-Wide RNAi Screen Implicates at Least 5 Host Hepatocyte Kinases in Plasmodium Sporozoite Infection
Source: PLoS Pathog. 2008 Nov 7;4(11):e1000201. doi: 10.1371/journal.ppat.1000201 (PMC2574010; doi:10.1371/journal.ppat.1000201)
Supplement: Figure S5 — Effect of siRNA-mediated in vivo silencing of PKCζ on PKCζ protein levels. The PKCζ protein levels were measured by Western blot analysis of liver extracts collected 40 h after sporozoite i.v. injection. Mice were infected 36 h after RNAi treatment with siRNA #1. The plot shows the quantification of the amounts of PKCζ normalised to those of actin (used as a housekeeping control protein) in liver samples of mice treated with the siRNA targeting the PKCζ gene, relative to the normalised amounts of PKCζ in control samples. (0.03 MB PDF) [file ppat.1000201.s006.pdf]

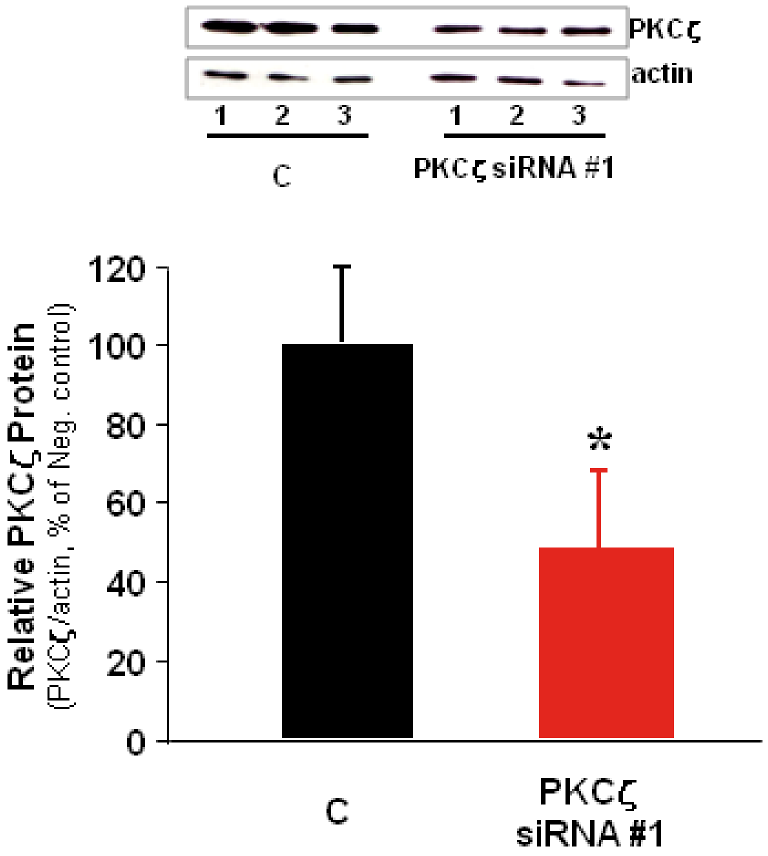

Figure S5. Effect of siRNA-mediated *in vivo* silencing of *PKCζ* on PKCζ protein levels.

The PKCζ protein levels were measured by Western blot analysis of liver extracts collected 40 h after sporozoite i.v. injection. Mice were infected 36 h after RNAi treatment with siRNA #1. The plot shows the quantification of the amounts of PKCζ normalised to those of actin (used as a housekeeping control protein) in liver samples of mice treated with the siRNA targeting the *PKCζ* gene, relative to the normalised amounts of PKCζ in control samples.
